# Extended validation of the mesh integration (MINT) index: a 1-year porcine study

**DOI:** 10.1007/s00464-026-12835-0

**Published:** 2026-05-04

**Authors:** Edward Young, Jean Wong, Alex Karatassas, Peter J. Hewett, John W. Finnie, Guy J. Maddern

**Affiliations:** 1 Department of Surgery, The Queen Elizabeth Hospital, Adelaide University (The University of Adelaide), Woodville South, South Australia Australia; 2 Basil Hetzel Institute, The Queen Elizabeth Hospital, Woodville South, South Australia Australia; 3 School of Biomedicine, Faculty of Health and Medical Sciences, Adelaide University (The University of Adelaide), Adelaide, South Australia Australia

**Keywords:** Hernia, Mesh, Tissue integration, Index, Pig, Translational research

## Abstract

**Background:**

Mesh Integration (MINT) index was previously proposed and validated in the short term as a standardised objective method of evaluating in vivo hernia mesh behaviour. The primary aim was to validate the degradation domain of the mesh integration (MINT) index over a 1-year period using a porcine model, with the secondary aim of determining integration and fibrosis scores after extended implantation.

**Methods:**

Six brands of mesh were implanted into three Landrace-White pigs within the retrorectus space. Post-mortems were performed at 1 year. All mesh-tissue samples were subjected to standardised testing specified by MINT. Previous 3-month study conditions were fully replicated.

**Results:**

Mesh was successfully implanted into all pigs, with an unremarkable 1-year natural history. There were no difficulties at post-mortem. Visually, all meshes were highly integrated. The 1-year degradation scores obtained were consistent with changes expected in absorbable meshes. Since study methodology, study conditions and mesh lot numbers were identical, the current study data were combined with the previous 3-month study for statistical analysis. Multi-level regression analysis with maximum likelihood was performed, and model diagnostics were conducted. Non-linear models achieved better fit to data than linear models, namely asymptotic for integration ($-2LL=76.46, {R}^{2}=0.971, RMSE=0.370$), biexponential for fibrosis ($-2LL=127.57, {R}^{2}=0.941, RMSE=0.517$) and logistic regression for degradation ($-2LL=199.83, {R}^{2}=0.984, RMSE=0.817$). Rationale and limitations to interpretation of the study results were extensively discussed.

**Conclusion:**

The degradation domain of the MINT index has been validated at 1 year. The versatility of the MINT index platform could potentially be used to summarise existing literature evidence on in vivo mesh behaviour.

**Supplementary Information:**

The online version contains supplementary material available at 10.1007/s00464-026-12835-0.

Incisional hernias after major abdominal surgery remain a material risk [1]. Whether performed prophylactically or during abdominal wall reconstructions [1–3], suture closure of defects with mesh reinforcement is the currently accepted method of stabilising a biomechanically unstable abdominal wall [4]. Provided early mechanical fixation is adequate [5], mesh-tissue integration is thought to be a key element necessary for long-term repair stability and recurrence prevention [6, 7]. Insertion of mesh induces an immediate localised inflammatory response, comprising beneficial tissue healing processes and less-desirable foreign body fibrotic reaction [8]. Excessive foreign body reaction with disruption of tissue regeneration is suspected of precipitating mesh shrinkage, seroma formation, chronic pain and eventual hernia recurrence [7, 9]. Much research has been devoted to mesh-tissue integration over recent decades, yet interpretation and comparison across different commercial and experimental mesh products have been difficult due to variations in study methodologies [10, 11]. The mesh integration (MINT) index was proposed to address this problem [12].

The MINT index is a standardised objective ratio-scale system that assesses in vivo hernia mesh behaviour, using a combination of pre-existing readily available testing tools and scoring sheets [12]. Four domains of integration, fibrosis, degradation and adhesions are assessed with each domain receiving a score between 0.0 (minimal effect) and 5.0 (maximal effect), calculated based on test results (Table 1). The MINT Index allows results from different studies to be converted to a standardised score and compared, provided study conditions (e.g. mesh brand, layer insertion, adjuncts) are sufficiently similar. The MINT index was previously validated by a 3-month porcine study, demonstrating the feasibility of using MINT to assess tissue-level changes [13]. The short duration of the 3-month study was a significant limitation, as it provided no indication on how degradation scores might function in the long-term; no indication on the long-term behaviour of integration and fibrosis scores; and no indication on the safety of the porcine model and effects of weight gain in the long term.

**Table 1:** Mesh Integration (MINT) Index [12]

Score	I—Integration	F—Fibrosis	D—Degradation	A—Adhesion
> 4.0 to ≤ 5.0	Maximal	Maximal	Maximal	Maximal
> 3.0 to ≤ 4.0	Extensive	Extensive	Extensive	Extensive
> 2.0 to ≤ 3.0	Moderate	Moderate	Moderate	Moderate
> 1.0 to ≤ 2.0	Mild	Mild	Mild	Mild
0.0 to ≤ 1.0	Minimal	Minimal	Minimal	Minimal

The primary aim of this study was to validate the degradation domain of the mesh integration (MINT) index over a 1-year period using a porcine model, with the secondary aim of determining integration and fibrosis scores after extended implantation.

## Materials and methods

The study was approved by the South Australian Health and Medical Research Institute (SAHMRI) Animal Ethics Committee (AEC) in November 2024 with reference number SAM-24-092. The study was pre-registered with the Animal Study Registry in November 2024, accessible at 10.17590/asr.0000371.

### Overview

The methodology from the 3-month porcine study was used as it is, with some minor modifications based on previous reflections [13]. For conciseness, only pertinent items are described in the following sections.

### Study location

Animal work was conducted at the SAHMRI Preclinical Imaging and Research Laboratories (PIRL). Sample analysis was performed at the Queen Elizabeth Hospital, the Basil Hetzel Institute, the University of Adelaide Histology Services, Adelaide Microscopy, and Adelaide Spectroscopy.

### Study duration, sample size, species

This study had a single time point of 1-year. This was a balance between ensuring the safety of the pigs and being representative of the long-term trend, as recommended by the International Organization of Standardization (ISO) 10993-6:2016 [14]. The long-term effect and behaviour of mesh implantation in pigs were unclear. Previously, dual layer placement (retrorectus and intraperitoneal) in pigs led to the development of ileus and slower recovery. It was postulated that a single layer, such as retrorectus, with avoidance of peritoneal entry may lead to a faster recovery and possibly a safer option over time [13]. Until this was tested, the outcome remained uncertain.

As the MINT index translates test scores to a standardised value, data may be reused if study conditions remain the same. In other words, by implanting the exact same meshes under the exact same experimental conditions as the initial validation study, data prior to 3 months do not need to be recollected, minimising the number of animals required for the study. A minimum of three pigs per time point was still desirable from a statistical point of view, due to better tolerance of any potential outliers.

Three female Landrace × Large White pigs, aged 11–12 weeks, weighing 30–35 kg, were acquired from a SAHMRI-approved piggery. Pig strain and sex were kept the same as the previous study to minimise data variability due to species and gender. The initial study recorded an average weight gain of 3.9 kg/week [13].

### Mesh choice and acquisition

The retrorectus layer was chosen for this study due to its greater clinical relevance and less risk of injury to intra-abdominal organs. Six mesh brands were utilised (Table 2). All meshes were synthetic, two-dimensional and approved for clinical use in the retrorectus layer. All meshes were from the same batch and lot numbers as the initial study [13]. Five out of six meshes were expired at the time of usage.

**Table 2:** Implanted mesh, using the same products from previous study [13]

Location	Expired (months)	Mesh	Company	Primary composition	Secondary composition	Minimum pore size (mm)	Estimated klinge mesh class	Mesh weight (g/m^2^)	Estimated coda mesh class
RR	15	Polypropylene	Bard	Polypropylene	–	0.46	IIa	95	S
RR	18	Soft	Bard	Polypropylene	–	2.5	Ia	44	L
RR	8	Parietene macroporous	Medtronic	Polypropylene	–	2.0	Ia	46	L
RR	7	Progrip	Medtronic	Polyethylene	Poly-l-lactic acid	1.1	Ib	73 (38)	L
RR	2	Phasix	Bard	Poly-4-hydroxybutyrate	–	0.26	IIb	182	H
RR	No	TIGR	Novus scientific	Polyglycolide acid/Poly-l-lactic acid/trimethylene carbonate	–	1.0	Ib	182	H

Table information sourced from the literature and respective manufacturer websites [50–54]

(): indicates change in mesh weight after loss of absorbable components over time

*RR* retrorectus

### Mesh storage and sterility

Unused meshes from the initial study had been sealed within clean labelled Ziplock bags and stored in a cool dry dark environment. Meshes were retrieved and trimmed to 50 × 50 mm size using sterile surgical instruments, before being individually packaged into sterilisation pouches. Non-implanted non-resterilised mesh pieces from the same batch were set aside as controls.

Pouches were resterilised via a standard 14 h ethylene oxide (EO) cycle inside a sterilisation cabinet. Sterility was confirmed by both chemical and biological exposure indicators. All meshes underwent only a single sterilisation cycle to ensure it was the same as the initial study [13]. Multiple sterilisation cycles have been shown to alter structural strength [15]. Non-implanted resterilised meshes were set aside as controls. TIGR was individually packaged and no additional preparation was required.

### Mesh labelling and randomisation

All mesh pieces were assigned a unique alphanumeric code for tracking and grouped by mesh brand. A mesh set was formed by choosing six packets using a random number generator (RNG) [16]. The RNG was configured such that each set contained all six mesh brands. The mesh placement location was also randomly generated and included within each set. At the time of operation, a random set was selected for use.

Mesh positions within the retrorectus layer were identical to those in the previous study, namely a column of three meshes behind each rectus muscle. Each mesh within a column was spaced 10 mm from the midline, and 10 mm from each other. A visual diagram was previously made available as supplementary material [13].

### Mesh dimensions

Photos of mesh were taken with an American Board of Forensic Odontology (ABFO) No. 2 right angle photomacrographic ruler as reference [17]. Ruler graduations were verified with a pristine vernier calliper prior to usage. Photos were taken at the time of package opening, before and after sterilisation if applicable, before implantation, and after implantation.

### Pre-operative

Upon arrival from the piggery, all pigs were given a 2 ml intramuscular inactivated Glaesserella parasuis vaccination against mycoplasma (Respisure® HPS Vaccine, Zoetis Australia Pty Ltd, Australia) as per SAHMRI policy.

For 7 days, pigs were housed in shared pens to socialise and acclimatise to the new environment, the technicians and research personnel. The living area was made comfortable with rubber mats, a radio and enriched with suitable toys for pigs to explore and play. The pigs were given access to ad libitum water and twice-daily pig chow as per standard SAHMRI protocols.

### Peri-operative

At 72 h prior to the operation, all pigs were moved to perioperative pens and assessed for wellness for surgery by PIRL staff. All three pigs were noted to be in good health and suitable for the study.

At 24 h prior to the operation, a 100 µg fentanyl transdermal patch (Duragesic® Patch, Johnson & Johnson, Australia) was applied to the back of an ear on each pig. Pigs were naturally fasted overnight following consumption of evening feeds at 1600 h. Water remained ad libitum throughout, and breakfast was held on the day of surgery.

On day of surgery, pigs were sedated by PIRL animal technicians, using intramuscular xyalazine 1–2 mg/kg (Randlab Xylazine 100 Injection, Randlab Australia, Australia) and intramuscular ketamine 10 mg/kg (Ranlab Ketamine Injection, Randlab Australia, Australia). Induction was achieved using 5% isoflurane/oxygen inhalation mixture, delivered via a snout mask from a Datex-Ohmeda 7100 anaesthetic machine (General Electric Healthcare, Australia) until no jaw tone was present. The pig was intubated using a suitably sized endotracheal tube and anaesthesia was maintained with 3% isoflurane/oxygen mixture.

The existing fentanyl patch was exchanged for a fresh 100 microg fentanyl transdermal patch. Penicillin B/G premix 10 mg/kg was given intramuscularly (Benecillin, Troy Laboratories Pty Ltd, Australia). Once asleep, pigs were moved to the operating theatre, positioned in a supine position on a heated mat, and secured to the operating table with straps.

### Operative

The pig’s abdomen was shaved with removal of loose hair and the surface was washed with 10% povidone-iodine solution (Vetsense, Australia). The operative members scrubbed, gowned and gloved according to established standards [18]. The abdominal wall was covered with sterile drapes and a single layer of iodine film (3M® Ioban® 2, 3M, Australia) was applied to the operative field.

A 30–40 cm midline incision was made using a No. 22 blade. Layers were carefully dissected using monopolar diathermy (Covidien, Australia) and the retrorectus space was entered from the midline. Care was taken not to enter the wrong plane. The retrorectus space was fully developed.

Mesh pieces were placed into their corresponding positions as predetermined by RNG and sutured against the retrorectus muscle at 9 points using size 3/0 polydioxanone sutures with a heavy blunt tapered needle (DemeDIOX, DemeTECH Corp., United States).

The anterior fascia was closed with continuous size 0 glycomer 631 sutures with a blunt needle (Biosyn, Medtronic®, Australia). The skin was closed with subcuticular size 3/0 poliglecaprone 25 sutures with a reverse-cutting needle (DemeCAPRONE, DemeTECH Corp., United States). A double layer of spray-on transparent dressing was applied to cover the midline wound (Opsite® Spray, Smith and Nephew, Australia) for waterproof protection and to facilitate visual inspection.

### Post-operative

Pigs were extubated and monitored using predefined clinical record sheets (CRS) [13]. All pigs were given a 5-day post-operative course of oral amoxicillin with clavulanic acid 12.5–15 mg/kg twice daily (Clavamox, Pharmacor Pty Ltd, Australia). Fentanyl patches were exchanged every 72 h for analgesia, and oral meloxicam 0.4 mg/kg crushed tablet with syrup was provided twice daily (Meloxicam, Troy Laboratories Pty Ltd, Australia) until the pigs’ CRS scores returned to baseline. Lactulose syrup was given as needed (Actilax, Mylan, Australia).

Pigs were returned to their indoor shared pens once sufficiently recovered, with the pens co-located next to each other to facilitate playful behaviour and socialisation. Items of enrichment, such as a musical radio and age-appropriate toys, continued to be provided. Water remained ad libitum and twice-daily pig chow as per standard SAHMRI protocols continued to be provided. With increasing size, greater space was required and the pigs were moved to the outdoor paddocks. A large shed and tree canopy sheltered the pigs from the elements, whilst hay and mud allowed pigs to wallow and play. Pigs were regularly reviewed to monitor wound status and general wellbeing.

### Post-mortem

All three pigs were euthanised at 1 year via a lethal dose of intravenous sodium pentobarbitone 160 mg/kg (Valabarb®, Jurox Pty Ltd, Australia). Death certification was performed by PIRL animal technicians.

Pigs were positioned supine on a large post-mortem table. A transverse incision was made in the lower pelvis, with careful dissection through the layers until the rectus muscles were identified. On each side, a small opening was made along the linea semilunaris to enter the retrorectus space, marked by prominent neurovascular bundles. Care was taken not to enter the preperitoneal layer. Vessels were cauterised with diathermy.

Using the boundary of the linea semilunaris as a guide, the openings were elongated cranially towards the costal margin. From a lateral to medial direction, the posterior rectus sheath was gently separated from the posterior aspect of the rectus muscles until all mesh pieces were confidently identified.

### Tissue extraction

Embedded meshes were photographed with an American Board of Forensic Odontology (ABFO) No. 2 right-angle photomacrographic ruler as a reference. Each sample was divided accordingly [13]. Control samples were taken from the rectus muscle, located away from regions of mesh placement.

All samples were labelled with a pre-generated alphanumeric code to maintain blinding. The tissue block for histology was placed in 10% neutral buffered formalin, and the remaining tissue blocks were kept in 0.9% phosphate buffered saline (PBS). Fresh tissue samples were transported at 4 °C in an ice-water bath, and stored at the same temperature until usage. All waste was disposed of according to local facility policies.

### Pre-test processing

Samples intended for uniaxial tensile testing, scanning electron microscopy and infrared spectroscopy were submerged in 40 ml of 0.1 M sodium bicarbonate solution and 10 ml of > 2.4 U/g protease from Bacillus licheniformis (Alcalase® 2.4L, Sigma-Aldrich/Merck, Germany) [19]. The meshes were incubated at 58 °C for 24 h. Samples were rinsed with deionised water, and clearance of biological tissue was confirmed by the absence of an amide peak between 1630 and 1700 cm^−1^ on FTIR [20]. Samples were stored in 70% isopropyl alcohol and air dried for 1 h in a biosafety cabinet prior to usage.

### Visual assessment

Photos were analysed using FIJI analysis software (v1.54, National Institutes of Health, United States) [21]. Images were corrected for distortion (Interactive Perspective) and calibrated for size (Set Scale). Area of mesh and area of integration were measured three times (Freehand Selection) and the average value was used.

Mesh surfaces were examined and scored using the degradation scoring sheet [12]. Light microscopy was performed using an Olympus BX45 light microscope (Olympus Corporation, Japan) under ×100 magnification. Scanning electron microscopy (SEM) was performed using an FEI Quanta FEG 450 (FEI Company/Thermo Fisher Scientific, United States). Mesh samples were coated with platinum and scanned under high vacuum with a voltage of 5.0 kV at a working distance of 10 mm. Key images observed under ×1000 magnification were recorded.

### Histological assessment

Tissue samples were fixed in 10% neutral buffered formalin, paraffin-embedded, and 5-micron sections cut and stained with haematoxylin and eosin (H&E). Duplicate sections were stained by the Masson’s trichrome technique to demonstrate collagen. Slides were sent to a blinded pathologist for grading, using the histology scoring sheet [12].

### Biomechanical assessment

Testing was conducted using a HUDA HD-B609-S tensiometer, with a 2 kN load cell, calibrated to ASTM E4 standards [22]. Shear testing was conducted within 6 h of post-mortem. Uniaxial tensile testing was performed after completion of protease cleaning.

### Molecular assessment

ATR-FTIR was performed using an IRSpirit spectrometer with QATR-S attachment and diamond prism (Shimadzu Incorporation, Japan). Spectra were obtained from three random spots on each sample, along with background spectra. Spectra were analysed using Spectragryph version 1.2 (Friedrich Menges Software-Entwicklung, Germany). Areas under the curves were obtained for all spectra of each sample and averaged before further calculations.

### Statistical analysis

Statistical analysis was performed using RStudio software (v2024.09.1.394, Posit Team, the United States), [23] running R language (v4.4.2, R Core Team, Austria) [24]. Kendall’s tau correlation tests were performed using R’s base system. Where applicable, multi-level linear model regression analyses, using maximal likelihood, were performed using car and lme4 packages [25, 26]. Likewise, multi-level non-linear model regression analysis, using maximal likelihood, was performed using lme4 package [27]. Contrasts were predefined and centring was not performed. Robust standard errors of cluster robust version 2 (CR2) were not applicable to non-linear modelling [28, 29]. Graphs were produced using ggplot2 package [30]. Models were compared using performance package [31]. The statistical significance level was defined as $p<0.05$.

## Results

Previous study conditions were replicated. Meshes (50 × 50 mm) were all successfully implanted into the retrorectus layer in all pigs with an operative time of 1.5 h. CRS scores required 2–4 days to return to baseline. (Supplementary Table 1). Analgesia requirements were minimal and limited to the initial 3 days as anticipated.

All pigs remained well throughout the 1-year study period. One pig sustained a minor superficial cut on its hoof at 3 months post-operative and was uneventfully treated with a course of antibiotics. There was little weight variation amongst pigs (Supplementary Fig. 1). Average weight gain for the pigs was 2.9 kg/week over the study period.

There were no difficulties with post-mortems. The midline laparotomy scar was near invisible on all pigs. All meshes were located in expected regions, with increased distance noted between adjacent meshes and between the mesh and anatomical landmarks. The majority of meshes were highly integrated with firm tissue strands traversing mesh pores. Surgical sutures were no longer identifiable.

**Fig. 1:**
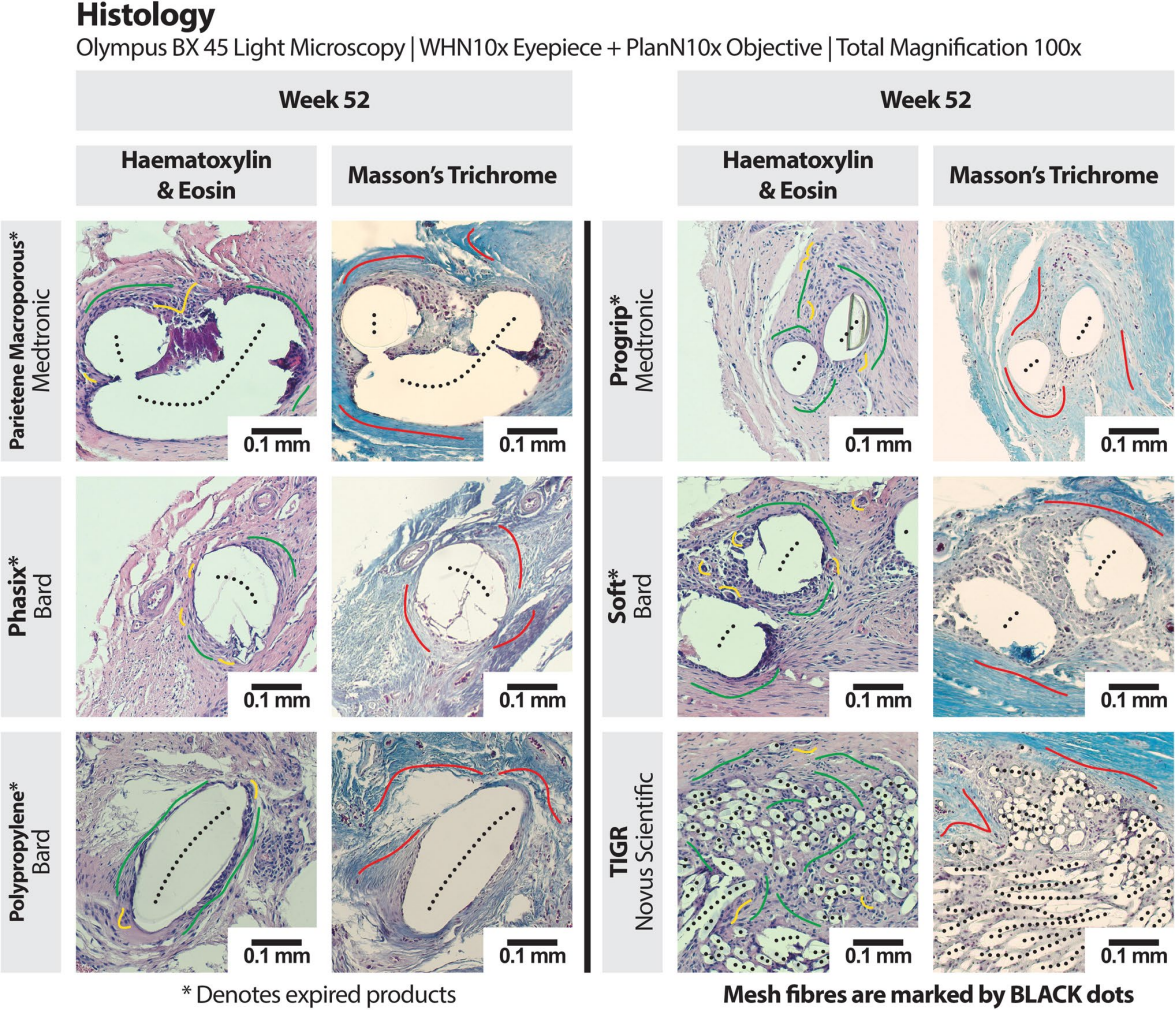
Histology of mesh at 1 year, stained with both haematoxylin and eosin, and Masson’s trichrome. Images have been taken under the same instrument settings and magnification as per previous publication to facilitate visual comparison [13]. Green lines mark macrophages, yellow lines mark giant cells, red lines mark collagen (Color figure online)

**Fig. 2:**
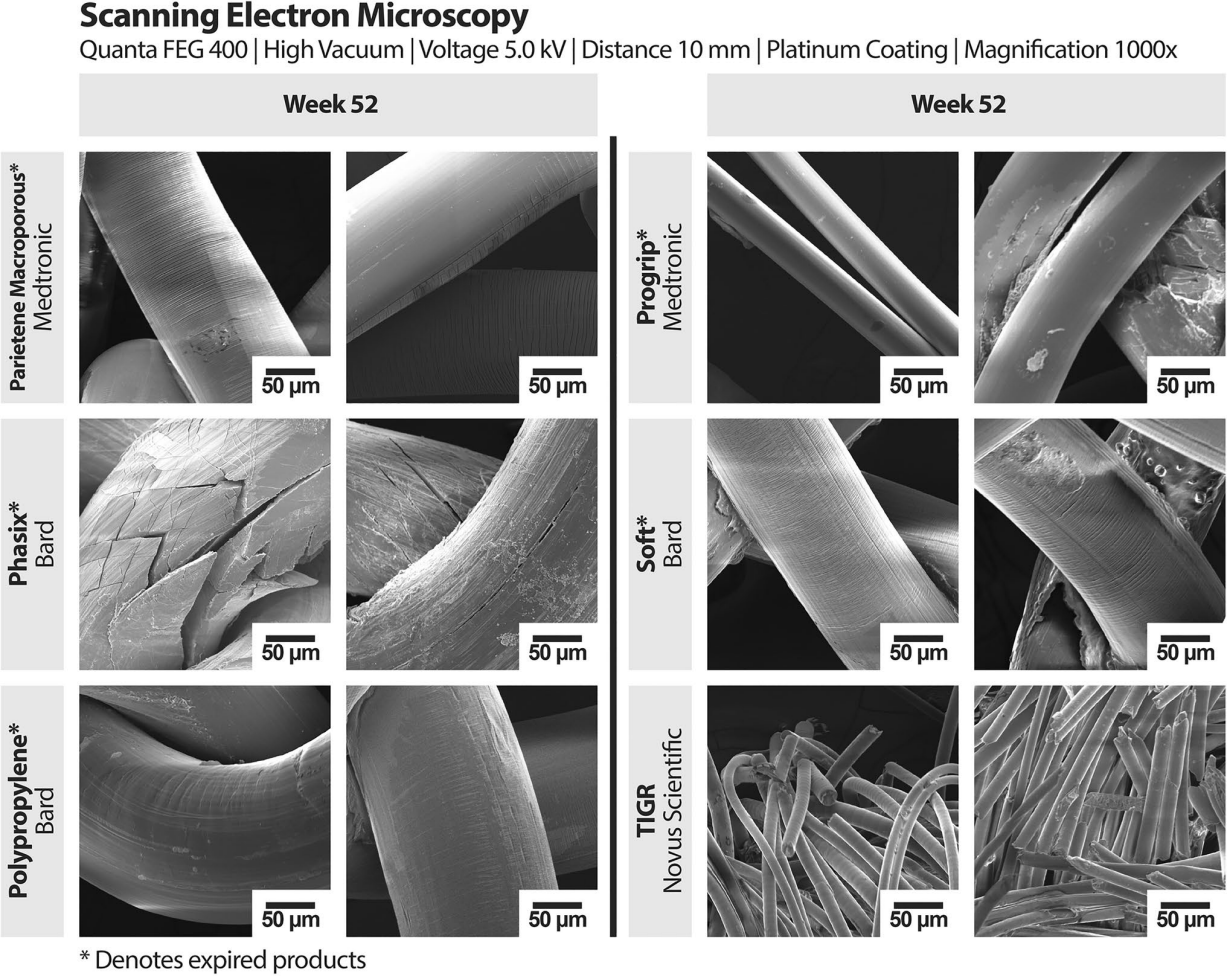
Scanning electron microscopy of mesh fibres at 1 year, demonstrating fracturing and cracking along the surface. Two representative images are shown for each mesh brand. All six meshes are shown. Images have been taken under the same instrument settings and magnification as per the previous publication to facilitate visual comparison [13]

All samples were processed as planned and remained clearly identifiable. Domain assessments were conducted as per MINT protocols [12]. Histological staining observed a chronic granulomatous (foreign body) inflammatory reaction (Scores 2 to 4 for fibrosis), characterised by an epithelioid macrophage and multinucleated giant cell response surrounding the implant material (Scores 1 to 3 for giant cells), with sometimes focal or multifocal mineralisation (Scores 0 to 3 for mineralisation) and variable numbers of infiltrating lymphocytes (Scores 0 to 2 for lymphocytes) (Fig. 1). There was extensive fibroblastic and attendant collagen deposition (Scores 2 to 4 for connective tissue deposition), with invasion into, and encapsulation of, the implant material (Scores 3 to 4 for fibrous encapsulation), along with variable degradation of the implant material, from minimal to almost total (Scores 1 to 4 for implant degradation). Phasix and TIGR were notably more structurally comprised at 1 year compared to their appearance previously recorded in the initial 3-month study (Fig. 2). [13]

**Fig. 3:**
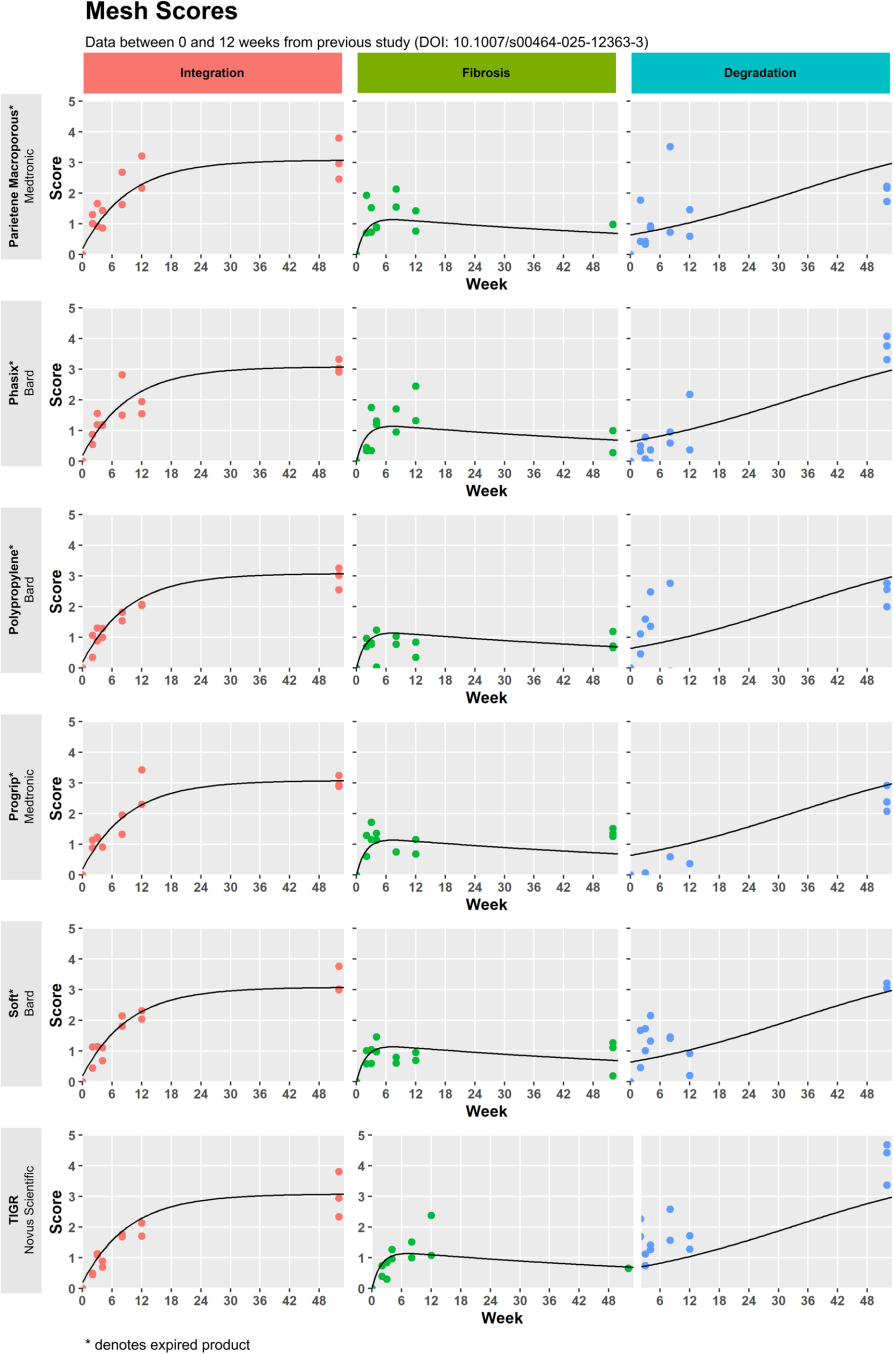
Index scores plotted over time (weeks). Adhesion scores not applicable. Negative values are not shown to avoid distortion of the chart. Scores between 0 and 12 weeks from previous study (https://doi.org/10.1007/s00464-025-12363-3) [13]. Global multi-level non-linear regression models are displayed in black lines within each graph, with asymptotic for integration, biexponential for fibrosis, and logistic for degradation

Index scores were calculated (Table 3, Supplementary Tables 2, 3 and 4). Adhesion scores were not applicable and were omitted. Integration and degradation scores at 1 year were generally high, and fibrosis scores were generally low. When plotting both the current study’s data and the previous 3-month data from healthy pigs, individual trends could be observed (Fig. 3). The relationships appeared to be a logistical growth for integration; an initial peak and long-term plateau for fibrosis; and a linear or curvilinear trend for degradation.

**Table 3:** Index scores of mesh after 1 year placement in retrorectus position, along with mean and standard deviation

The index			Module scores				Mean scores (SD)			
Mesh name	Time (weeks)	Position	Integration	Fibrosis	Degradation	Adhesion	Integration	Fibrosis	Degradation	Adhesion
Parietene macroporous* Medtronic	52	RR	2.65	0.97	1.74	–	2.50 (0.22)	0.50 (0.84)	1.67 (0.27)	–
	52	RR	2.60	0.99	1.37	−				
	52	RR	2.25	− 0.47	1.90	−				
Phasix* Bard	52	RR	1.94	0.28	3.32	−	2.33 (1.51)	0.28 (0.72)	3.71 (0.38)	–
	52	RR	2.13	− 0.43	4.08	−				
	52	RR	2.91	1.00	3.77	−				
Polypropylene* Bard	52	RR	2.24	0.72	2.72	−	2.35 (0.44)	0.86 (0.29)	2.43 (0.42)	–
	52	RR	2.84	0.66	2.61	−				
	52	RR	1.98	1.19	1.95	−				
Progrip* Medtronic	52	RR	2.47	1.52	2.64	−	2.49 (0.23)	1.38 (0.13)	2.26 (0.47)	–
	52	RR	2.73	1.36	2.40	−				
	52	RR	2.28	1.26	1.73	−				
Soft*Bard	52	RR	2.69	1.11	2.88	−	2.82 (0.79)	0.86 (0.58)	2.95 (0.23)	–
	52	RR	3.67	0.19	2.76	–				
	52	RR	2.11	1.27	3.21	–				
TIGR Novus Scientific	52	RR	2.58	− 0.60	4.69	–	2.21 (0.32)	0.24 (0.72)	3.75 (0.82)	–
	52	RR	2.02	0.66	3.37	–				
	52	RR	2.03	0.65	3.18	–				

*RR* retrorectus; *SD* standard deviation

^*^denotes expired mesh; – data not available

Under the assumption of independence and non-parametric small data set, correlation analysis using Kendall’s Tau (${\tau }_{Kendall}$) suggested strong relationships of time with integration ($p<0.001$) and time with degradation ($p<0.001$) (Supplementary Table 5). There was a weak non-significant relationship of time with fibrosis. Mesh was not significantly correlated with any scores ($p>0.05$).

To account for data clustering and interactions, regression analysis using multi-level linear and non-linear modelling was performed (Supplementary Table 6). Three levels were defined, in the order of pig, mesh and domain scores. Duration of implantation (Time) was defined as a fixed factor, with pig and mesh defined as random factors. The purpose of modelling was to demonstrate behaviour of domain scores over time. Standard models were chosen, including linear regression, polynomial regression (2nd, 3rd and 4th orders), logistic regression, asymptotic regression and biexponential regression. There was insufficient data to examine linear models with mesh as a fixed factor or any non-standard custom-made non-linear models.

Fitness of models was assessed with model diagnostics performed for all models, including residue linearity, homogeneity of variance, quantile–quantile plots (Q-Q plot) and residual normality (Supplementary Figs. 2  to 25 ,). The intraclass correlation coefficient (or variance partition coefficient) was not calculated for non-linear multi-level models due to condition-dependent interpretation, as previously explained by Goldstein et al. [32], nor calculated if there were singularity effects. For brevity, the following symbols were used: minus 2 log-likelihoods ($$-2LL$$), root mean square error ($$RMSE$$), intraclass correlation coefficient ($$ICC$$), R squared ($${R}^{2})$$ and Chi-square p-value ($$p)$$. A good fitness of model was defined as the presence of a low $$-2LL$$, a low $$RMSE$$, a high $$ICC$$ (if available) and good distribution of residues on diagnostic plots, whilst still maintaining visual validity with avoidance of overfitting.

For integration, the introduction of time as a fixed factor into a linear model with variable intercept amongst crossed random effects (pig and mesh) led to a significant improvement ($$p<0.001)$$ in fit ($$-2LL=103.56, {R}^{2}=0.662, RMSE=0.331, ICC=0.614$$), compared to the base model ($$-2LL=123.30, {R}^{2}=0.0, RMSE=0.340, ICC=0.863$$) (Supplementary Table 6). The addition of 2nd, 3rd and 4th order polynomial improved fitness to data $$(-2LL=79.76, {R}^{2}=0.846, RMSE=0.347 \left| -2LL=75.86, {R}^{2}=0.856,\,RMSE=0.353 \right|-2LL=75.22, {R}^{2}=0.858,\,RMSE=0.354$$) but at the expense of overfitting and loss of visual validity ($$ICC=0.149 \left| ICC=0.087 \right| ICC=0.078$$). Non-linear models achieved better fit than the original linear model, namely logistical regression ($$-2LL=86.30, {R}^{2}=0.617, RMSE=0.370$$) and asymptotic regression ($$-2LL=76.46, {R}^{2}=0.971, RMSE=0.370$$). The shape of the data was not suitable for a biexponential model. Diagnostic plots of the two non-linear models were similar and did not reveal any significant skew in residuals or heteroscedasticity. With a passage close to the origin, the asymptotic regression model was visually more sensible than the logistic regression model (Supplementary Figs. 8 and 9).

For fibrosis, the same linear model with time as a fixed factor, and with pig and mesh as random factors failed to achieve any improvement ($-2LL=146.34, {R}^{2}=0.013, RMSE=0.521, ICC=NA$) over the base model ($-2LL=146.85, {R}^{2}=0.0, RMSE=0.521, ICC=0.156$). Polynomial regression of 2nd and 3rd orders achieved significantly better fit ($-2LL=137.87, {R}^{2}=0.144, RMSE=0.541, ICC=NA, p=0.004 |-2LL=131.47, {R}^{2}=0.206, RMSE=0.529, ICC=NA, p=0.063$) than the linear model. Increasing the polynomial regression to 4th order achieved a slightly tighter fit ($-2LL=127.07, {R}^{2}=0.247, RMSE=0.516, ICC=NA$) but was not significantly different from the 3rd order model ($p=0.428$). A logistical regression was not possible due to the shape of the data. An asymptotic regression achieved a slightly better fit ($-2LL=134.92, {R}^{2}=0.392, RMSE=0.540$) but was not substantially different from the 2nd order polynomial regression. A substantially better fit was achieved with a biexponential model ($-2LL=127.57, {R}^{2}=0.941, RMSE=0.517$). Diagnostic plots showed good linearity, near-normal distribution of residuals and homoscedasticity (Supplementary Fig. 17). This biexponential relationship could explain why the initial Kendall’s Tau test for linear relationship was non-significant, since the score is both increasing and decreasing with time.

For degradation, a linear model with time as a fixed factor had a significantly worse fit ($$-2LL=254.5, {R}^{2}=0.499, RMSE=0.718, ICC=0.335,p<0.001$$) than base model ($$-2LL=254.5, {R}^{2}=0.0, RMSE=0.870, ICC=0.487$$). Upgrading to a 2nd order polynomial regression made no difference ($$-2LL=235.87, {R}^{2}=0.499, RMSE=0.718, ICC=0.335, p=0.850)$$. A 3rd order polynomial regression achieved a significantly tighter fit ($$-2LL=208.60, {R}^{2}=0.630, RMSE=0.717, ICC=NA, p=0.003)$$. A further increase to 4th order polynomial regression was non-beneficial ($$-2LL=208.57, {R}^{2}=0.632, RMSE=0.714, ICC=NA, p=0.456).$$ Non-linear models had better fit, namely logistical regression ($$-2LL=199.83, {R}^{2}=0.984, RMSE=0.817)$$, and asymptotic regression ($$-2LL=199.28, {R}^{2}=0.855, RMSE=0.815).$$ A biexponential model was not possible due to data shape. Close analysis of diagnostic plots showed better linearity, residual normality and homoscedasticity in the logistical regression model, compared to the asymptotic regression model (Supplementary Figs. 24 and 25).

## Discussion

This study has validated the degradation domain of the MINT index by demonstrating domain score changes that were consistent with the behaviour of selected absorbable meshes after 1-year implantation within an established porcine model. Objective insights into the in vivo behaviour of integration and fibrosis of selected meshes implanted into the retrorectus layer were further extended from previous observations.

The methodology utilised in this study may be considered unorthodox, as it combined results from a prior 3-month study. Combining results from different studies is known to be a challenging process, as systemic biases may be inadvertently introduced. These risks and potential confounders were specifically addressed in the initial proposal of the MINT index, and the subsequent study designs. The MINT index utilised objective standardised tools to measure a broad range of technical specifications pertaining to the interface of the mesh-tissue sample [12]. Provided the measuring tools were calibrated to international standards, it was unlikely that intra- and inter-study measurements would experience significant variations. Objective measurements were prioritised over subjective assessment methods where possible, such as digitally measuring area sizes in calibrated photos as opposed to visual estimations. The limited scoring sheets utilised in the MINT index have been derived from established international standards and existing literature evidence. Inter-variability between measurements was further minimised by taking the average of repeated measurements or having multiple independent scorers. By design, the effect of outliers is reduced within the MINT index as all measurements are converted into a percentage equivalent and the average value is taken to form the domain score. Only a very large outlier has sufficient numerical power to strongly alter the domain score. Such outliers are easily detected and can be checked for errors.

To maintain consistency across the mesh-tissue samples obtained, the two studies were conducted with meticulous care, with careful replication of the study protocols, attention to experimental steps and transparent reporting. Mesh products utilised were the same and were from identical lots and batches. Meshes were sealed in waterproof bags and stored at standard laboratory temperature in a dry dark environment. Meshes were cut and sterilised one to two weeks prior to surgical implantation for each study using the same calibrated gas sterilisation unit with identical sterilisation protocols. The biological and chemical indicators of sterility for all utilised meshes were correctly activated. The effects of sterilisation agents on mesh tensile strength were further accounted for by testing randomly selected meshes as controls, before and after sterilisation, and utilising these tensile values within each respective study as the baseline tensile strength of meshes. This design took into consideration any potential variations in tensile strength due to manufacturing processes, structural alterations over time in expired meshes or how the mesh was cut. More meshes were prepared and sterilised than required for each study, allowing random selection of meshes. No mesh was resterilised a second time in order to eliminate any potential confounders in polymer integrity arising from repeated sterilant exposure [15, 33]. Intraoperatively, meshes were handled in the same fashion by trained surgeons, with the operating team remaining identical across the two studies. Peri-operative care was provided by the same team of veterinarians and animal technicians at the same research facility with no institutional protocol changes occurring during the two studies. All staff remained equally familiar and proficient between the studies, as the 1-year study commenced almost immediately after the conclusion of the 3-month study. Research animals were of the same strain, similar weight and age, and obtained from the same commercial piggery. More pigs were obtained than required by the research facility, and pigs were selected at random for the two projects. Pig husbandry was identical, with the provision of the same type of chow, water, enrichment activities and living environment.

There were two major differences between the two studies. The 1-year study implanted meshes only into the retrorectus layer, whilst the 3-month study implanted into both retrorectus and intraperitoneal layers. This was modified due to the experiences obtained in the 3-month study, both from a technical point of view and general tolerability by pigs in the immediate post-operative period. Additionally, the focus of the 1-year study was degradation, and this was deemed to be of more relevance to a mix of permanent meshes (e.g. bare polypropylene) and fully absorbable meshes implanted in the retrorectus layer, compared to the intraperitoneal layer. The method of anchoring meshes into the retrorectus space was also altered in the 1-year study. Meshes were sutured to the rectus muscles instead of the posterior rectus sheath in order to eliminate any risk of injuring small bowel mucosa, which had been observed to occur with some frequency in the original 3-month study. No gross bowel content spillage or rectus sheath haematoma occurred in either of the studies. A single pig from the 3-month study developed midline wound infection and the data associated with that pig was excluded from the combined analysis. The technical change to how the meshes were secured in the retrorectus layer was unlikely to have altered the tissue infiltrative process into the meshes, since they were still physically sandwiched between the rectus muscles and the posterior rectus sheath. Although there may have been differences in the speed of recovery by pigs between the two studies, the overall health status of pigs, reflected by their weight and growth rate, was not significantly different for the initial 3-month period between the studies [13].

The second major difference was the size of the pigs, due to further growth acquired by the pigs over the 1-year period. This was an expected development of the pig strain utilised and could not be easily avoided. The pigs maintained a steady growth despite a calorie-limited chow diet already stipulated in the standard feeding protocol utilised by the research facility. The growth rate would have been much greater if commercial levels of feeds were given with the intention for food production purposes [34]. Compared to the 3-month study, pigs at the end of the 1-year study had a much larger body habitus, with greater girth and length. These size changes likely would not have altered integration scores, as the MINT index compares size of tissue integration with the area of the mesh on calibrated photographs at the time of post-mortem. There was no evidence of any mesh dislodgement or secondary complications. Stretched mesh may have positively influenced the shrinkage subscores in the fibrosis domain, as the area of mesh at the time of post-mortem was compared to the post-sterilisation pre-implantation mesh area. The positive bias was likely minimal due to the averaging calculation required to obtain the domain score, and the fact that no significant alterations in mesh sizes (e.g. doubling in size) were visualised amongst implanted meshes at the time of post-mortem.

Although there are some differences between the two studies, the potential confounders identified have been largely mitigated by the design of the MINT index and they are unlikely to have biased the results. The analytic process utilised in this study has demonstrated the versatility of the MINT index in analysing big data from multiple studies, provided study parameters and mesh conditions are kept as constants. If a degree of variance is allowed between different studies and is accounted for through judicious application of multi-level regression modelling and consideration of clustering effects, then existing study results available in the literature could potentially be converted into the MINT index and be compared, without the need to repeat prior experiments. The efficient and reduced use of experimental animal numbers would align with the 3R principles of ethical scientific research [35, 36].

Of the data that were analysed, distinct patterns emerged within each domain, which appeared to have achieved better fits by non-linear regression models. It should be noted that only basic models have been utilised here due to the available data and that there is a very long list of advanced regression models that could be further tailored to the situation, depending on the variety of fixed effects, random effects and their interactions [37]. The purpose of modelling was to demonstrate the behaviour of meshes over time, and illustrate how objective measurements could be translated into mathematical modelling. There were insufficient data points to determine individualised models for each mesh brand; thus, mesh was kept as a random factor so that the overall trend could be investigated within the limits of the collected data. It remains a distinct possibility that different meshes may have different in vivo behaviours which may best correspond to different mathematical models. By modelling the in vivo behaviour, different mesh products and their behaviour under specific conditions may be visualised and compared in an objective fashion, thus fulfilling a key purpose of the MINT index.

The following is a discussion of the changes observed in the three domains examined, namely integration, fibrosis and degradation. These observations are to be interpreted within the parameters of the current study, and at this stage may not necessarily translate to all existing meshes, or even in date meshes of the same brand. This is due to several limitations of the study, such as the need to near-fully replicate the methodology of the 3-month study; tissue analysis limited to a single time point due to available research budget; uncertainty of whether pigs would tolerate the study parameters and reach the one-year study endpoint without complications. The mesh pieces were relatively small due to the confines of 12-week old pigs, limiting the amount of mesh-tissue samples available for analysis. Duplicate data within the same time points or alternate time points such as 18 or 24 months would be desirable in the future studies. With the flexibility of multi-level regression models, the underlying mathematical principles do not change and could be easily tailored to new data, including those that are derived from in date mesh products, along with the inclusion of new factors or clustering effects.

Integration scores across all six meshes showed substantial improvement over their scores at 3 months, though none scored 4.0 or above. The relationship appears to correspond to an asymptotic model. It was unclear whether this was a limitation of the data collected or whether this was due to an actual physical limit of integration being reached. Within the scoring system, integration score losses were predominantly due to persistent presence of immune cells, non-maximal cellular infiltration or neovascularisation and biomechanical shear testing being proportionally less than estimated physiological limits (Supplementary Table 2). All mesh samples were observed to have dense infiltration of tissue at the time of post-mortem. This seemed to suggest that macroscopic or optical observation of mesh-tissue infiltration may not necessarily reflect in situ biomechanical stability, highlighting the value of direct mechanical measurements. It is possible that tissue reorganisation may be required to establish biomechanical strength, though it is uncertain at this stage how this could be directly verified in an objective fashion.

An alternate theory is that the mechanical shear test only assessed one interface, yet a retrorectus mesh or any mesh sandwiched within layers would have the benefit of two surfaces. Shear forces along the mesh in the abdominal wall would normally need to overcome attachments on both sides to disrupt it, and perhaps the in vivo structural resistance may be better estimated by applying a doubling adjustment to the %shear ratio elastic and %shear ratio ultimate with the assumption that tissue integration on both sides of the mesh would be equivocal. Mathematically, the integration score would, at most, increase by 1.25 in value in its current configuration, and a slight upshift in results may be observed (Supplementary Fig. 26). This adjustment would naturally favour meshes sandwiched within layers, as opposed to intraperitoneal mesh or meshes bridging a defect that only has a single-sided contact. From a clinical utility perspective, integration scores should still continue to be calculated with single-sided shear to maintain objectivity, since mesh placement is dependent on the clinical scenario. In practice, the reference value could be ad hoc adjusted by a value of + 1 (decimals truncated) to account for the double-sided adhesive-like composite structure.

In healthy young pigs, fibrosis scores appear to be collectively lower in the long term, compared to the initial pronounced fibrotic activity. Only limited foreign body reactions took place, as all meshes were observed to have fibrous encapsulation with variable fibrosis and mineralisation at one year. A biexponential model achieved the best fit of the available data, compared to other basic models that were tested. This observation would be consistent with the change in intensity of fibrosis observed during typical wound healing, with initial molecular, biological and mechanical factors being highly fibrosis-inducing, which eventually trend towards fibrosis attenuating as the healing process transitions to completion [38]. The plateau observed could be a truncated tail that has not fallen to zero or an indication that a low level of fibrosis may persist in the presence of mesh as a foreign body and immunological stimulant. This could also be a reflection of the immune capabilities of young, healthy adult pigs, and may not necessarily translate to adult humans. Extended studies may shed further light on this phenomenon.

Degradation scores behaved as designed, scoring extremely high for the degradable Phasix and TIGR meshes at one year. In controlled testing environments, Phasix mesh has been reported to resorb at 12 to 18 months, whilst TIGR partially resorbs at 4 months and fully resorbs at 36 months [39]. A logistic regression achieved the best fit for the overall data, though a mesh-specific model would likely be more meaningful in delineating the specific differences between non-absorbable, partially absorbable and totally absorbable meshes, particularly with the addition of data at 18 and 24 months after implantation. Visually, all meshes at 1 year exhibited more fracturing and cracking compared to their initial pristine appearances seen under SEM [13]. The presence of biological tissue significantly interfered with electron microscopy imaging of absorbable meshes and hindered visual observations.

The degradation score identified changes within non-absorbable meshes. It was unclear if these were intended changes after prolonged implantation, artefacts from the heated enzymatic-based tissue removal process or expected changes in expired meshes when exposed to an oxidative environment. Due to the nature of the included assessments, the degradation domain score likely functions as a spectrum, with higher scores being more representative of polymer degradation. One could argue, perhaps, that the tools utilised in the current iteration of the degradation score in the MINT index may be too sensitive, and any damage to the mesh from external factors may be misattributed to in vivo degradation. In the current and previous studies, extreme care was taken when handling the meshes to minimise any possible external damage to the mesh. Repeating the study with in-date meshes could clarify this aspect. Alternatively, other tools that may be used to assess polymer degradation, such as thermogravimetric analysis or differential scanning calorimetry, could be investigated and introduced into the degradation domain score [40, 41].

The growth of pigs was substantial over the 1-year study period. Pig weights increased from mid-30 kg to almost 200 kg, with a craniocaudal distance lengthening from 70 cm to over 150 cm. These rates of growth and weight gain over a year typically do not occur in healthy humans. The closest equivalent would be the total height and weight changes observed between age 2 and age 18 on standard growth charts for children [42]. The growth demonstrated in the porcine model may have a role in providing further insights into the changes experienced by the abdominal wall of paediatric patients and in investigating the in vivo behaviour of hernia meshes, which may be required in special cases of abdominal wall reconstruction [43]. Likewise, the rapid growth in the 1-year porcine model may also be beneficial in investigating the biomechanical effects of post-operative weight gain following ventral hernia repairs, with the literature suggesting worse outcomes in patients who experience post-operative weight gain following midline abdominal incisions [44]. Weight gain has been previously shown to linearly correlate with intra-abdominal and subcutaneous fat gain, leading to an increased intra-abdominal volume, total visceral volume and subcutaneous volume [45]. If the anterior abdominal wall is approximated to a thin-wall cylindrical vessel in a static state, increasing intra-abdominal pressure and increased transverse distance (cavity diameter) from weight gain will lead to higher transverse stresses (hoop stress) within the abdominal wall as per pressure vessel stress mechanics, placing hernia repairs at greater risk of failure if post-operative weight gain does occur [46].

Even with these significant macroscopic changes, there were minimal changes observed at the time of the post-mortem, with no evidence of mesh dislodgement or migration within the products utilised. As explained earlier, any changes in mesh area would likely only affect the shrinkage subscore of the fibrosis domain and would be averaged out when calculating the domain score. For example, in the current iteration of the MINT index, expansion of the mesh area by an extra 20% would only translate to a raw score reduction of 0.5 in the overall fibrosis score. Excessive mesh elongation could theoretically reduce the fibrosis score to 0; however, elasticity of over 30% is considered undesirable in ventral hernia repairs [47].

To minimise effects of body growth, long-term studies should consider using pigs that are at least 1-year-old, where the growth rate appears to begin plateauing. Larger pigs are additionally advantageous in offering a much greater operative field, with the retrorectus width approaching 7–8 cm and the longitudinal distance reaching 50–60 cm, allowing more or larger pieces of mesh to be placed. Mesh placement within the retrorectus layer was very well tolerated in this study, compared to the dual layer placement in the initial study [13]. Pigs overall were highly resilient to the environmental elements and of relatively low maintenance over the 1-year study period. Drawbacks of using large pigs were the space required for housing and the need for dedicated animal research facilities with agricultural veterinarian expertise.

The clinical interpretation of the MINT index will need to be coupled with a clinical quality registry or equivalent, such that what is observed at the bench side can be correlated with clinical outcomes [13]. This is a compromise, since we cannot conduct human studies using the MINT index due to its destructive assessments and ethical reasons. Clinical quality registries require time and effort to set up, which is hopefully beginning to be streamlined and automated with the rise in neural network artificial intelligence in recent times [48, 49]. An immediately available utility of the MINT index is to review and quantify the existing studies that have examined mesh-tissue integration, degradation or peritoneal adhesions so far. By summarising the current data landscape, areas of deficiency could be identified to better inform the direction of future research into hernia mesh and the next generation of implants in abdominal wall reconstruction [13].

## Supplementary Information

Below is the link to the electronic supplementary material.

Supplementary file1 (PDF 118 KB)

Supplementary file2 (DOCX 8586 KB)

Supplementary file3 (PDF 60 KB)

Supplementary file4 (PDF 114 KB)

Supplementary file5 (DOCX 52 KB)

Supplementary file6 (PDF 99 KB)

## Data Availability

Data have been provided in the manuscript and supplementary materials.
