# Supplementary material for: Extended validation of the mesh integration (MINT) index: a 1-year porcine study
Source: Surg Endosc. 2026 May 4;40(7):5917–32. doi: 10.1007/s00464-026-12835-0 (PMC13369702; doi:10.1007/s00464-026-12835-0)

# Supplementary Figure Legend

**Supplementary Figure 1** – Pig weight over time

**Supplementary Figure 2** – Residual diagnostic plots for Integration Score. Model: Score ~ 1 + (1|Pig)

**Supplementary Figure 3** – Residual diagnostic plots for Integration Score. Model: Score ~ 1 + (1|Mesh) + (1|Pig)

**Supplementary Figure 4** – Residual diagnostic plots for Integration Score. Model: Score ~ Time + (1|Mesh) + (1|Pig)

**Supplementary Figure 5** – Residual diagnostic plots for Integration Score. Model: Score ~ poly(Time, 2) + (1|Mesh) + (1|Pig)

**Supplementary Figure 6** – Residual diagnostic plots for Integration Score. Model: Score ~ poly(Time, 3) + (1|Mesh) + (1|Pig)

**Supplementary Figure 7** – Residual diagnostic plots for Integration Score. Model: Score ~ poly(Time, 4) + (1|Mesh) + (1|Pig)

**Supplementary Figure 8** – Residual diagnostic plots for Integration Score. Model: Score ~ SSlogis(Weeks, Asym, xmid, scal) ~ (Asym|Mesh) + (xmid|Pig)

**Supplementary Figure 9** – Residual diagnostic plots for Integration Score. Model: Score ~ SSasymp(Weeks, Asym, R0, lrc) ~ (Asym|Mesh) + (R0|Pig)

**Supplementary Figure 10** – Residual diagnostic plots for Fibrosis Score. Model: Score ~ 1 + (1|Pig)

**Supplementary Figure 11** – Residual diagnostic plots for Fibrosis Score. Model: Score ~ 1 + (1|Mesh) + (1|Pig)

**Supplementary Figure 12** – Residual diagnostic plots for Fibrosis Score. Model: Score ~ Time + (1|Mesh) + (1|Pig)

**Supplementary Figure 13** – Residual diagnostic plots for Fibrosis Score. Model: Score ~ poly(Time, 2) + (1|Mesh) + (1|Pig)

**Supplementary Figure 14** – Residual diagnostic plots for Fibrosis Score. Model: Score ~ poly(Time, 3) + (1|Mesh) + (1|Pig)

**Supplementary Figure 15** – Residual diagnostic plots for Fibrosis Score. Model: Score ~ poly(Time, 4) + (1|Mesh) + (1|Pig)

**Supplementary Figure 16** – Residual diagnostic plots for Fibrosis Score. Model: Score ~ SSasymp(Weeks, Asym, R0, lrc) ~ (Asym|Mesh) + (R0|Pig)

**Supplementary Figure 17** – Residual diagnostic plots for Fibrosis Score. Model: Score ~ SSbiexp(Time, A1, lrc1, A2, lrc2) ~ (A1|Mesh) + (A2|Pig)

**Supplementary Figure 18** – Residual diagnostic plots for Degradation Score. Model: Score ~ 1 + (1|Pig)

**Supplementary Figure 19** – Residual diagnostic plots for Degradation Score. Model: Score ~ 1 + (1|Mesh) + (1|Pig)

**Supplementary Figure 20** – Residual diagnostic plots for Degradation Score. Model: Score ~ Time + (1|Mesh) + (1|Pig)

**Supplementary Figure 21** – Residual diagnostic plots for Degradation Score. Model: Score ~ poly(Time, 2) + (1|Mesh) + (1|Pig)

**Supplementary Figure 22** – Residual diagnostic plots for Degradation Score. Model: Score ~ poly(Time, 3) + (1|Mesh) + (1|Pig)

**Supplementary Figure 23** – Residual diagnostic plots for Degradation Score. Model: Score ~ poly(Time, 4) + (1|Mesh) + (1|Pig)

**Supplementary Figure 24** – Residual diagnostic plots for Degradation Score. Model: Score ~ SSlogis(Weeks, Asym, xmid, scal) ~ (Asym|Mesh) + (xmid|Pig)

**Supplementary Figure 25** – Residual diagnostic plots for Degradation Score. Model: Score ~ SSasymp(Weeks, Asym, R0, lrc) ~ (Asym|Mesh) + (R0|Pig)

**Supplementary Figure 26** – Hypothetical integration scores if biomechanical shear stress values are adjusted to account for double-sided tissue contact. Note the upshift of plots from original positions (Red Circles), to the adjusted values (Green Triangles)

# Supplementary Figure 1

Pig weight over time


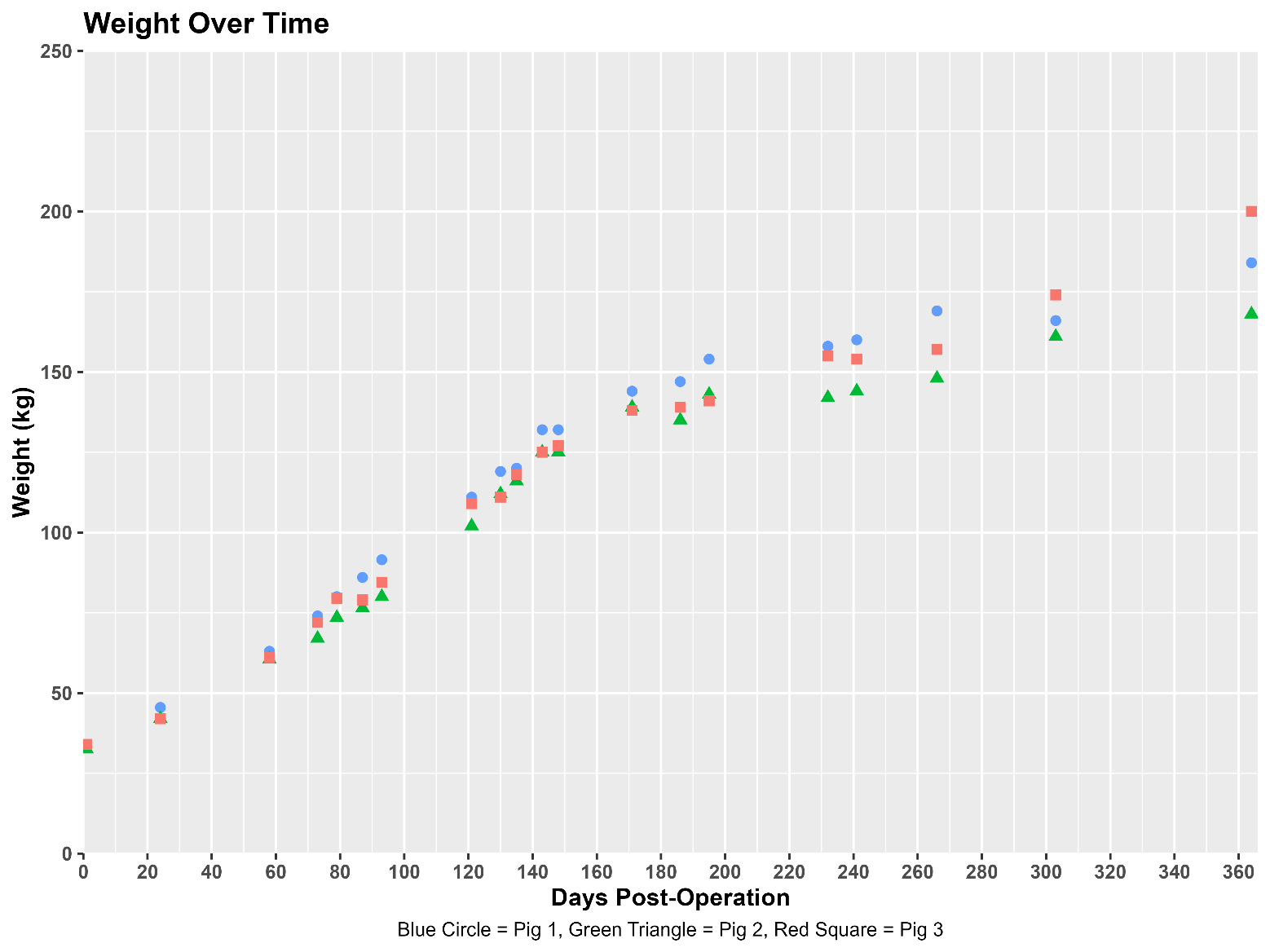


# Supplementary Figure 2

Residual diagnostic plots for Integration Score. Model: Score ~ 1 + (1|Pig)


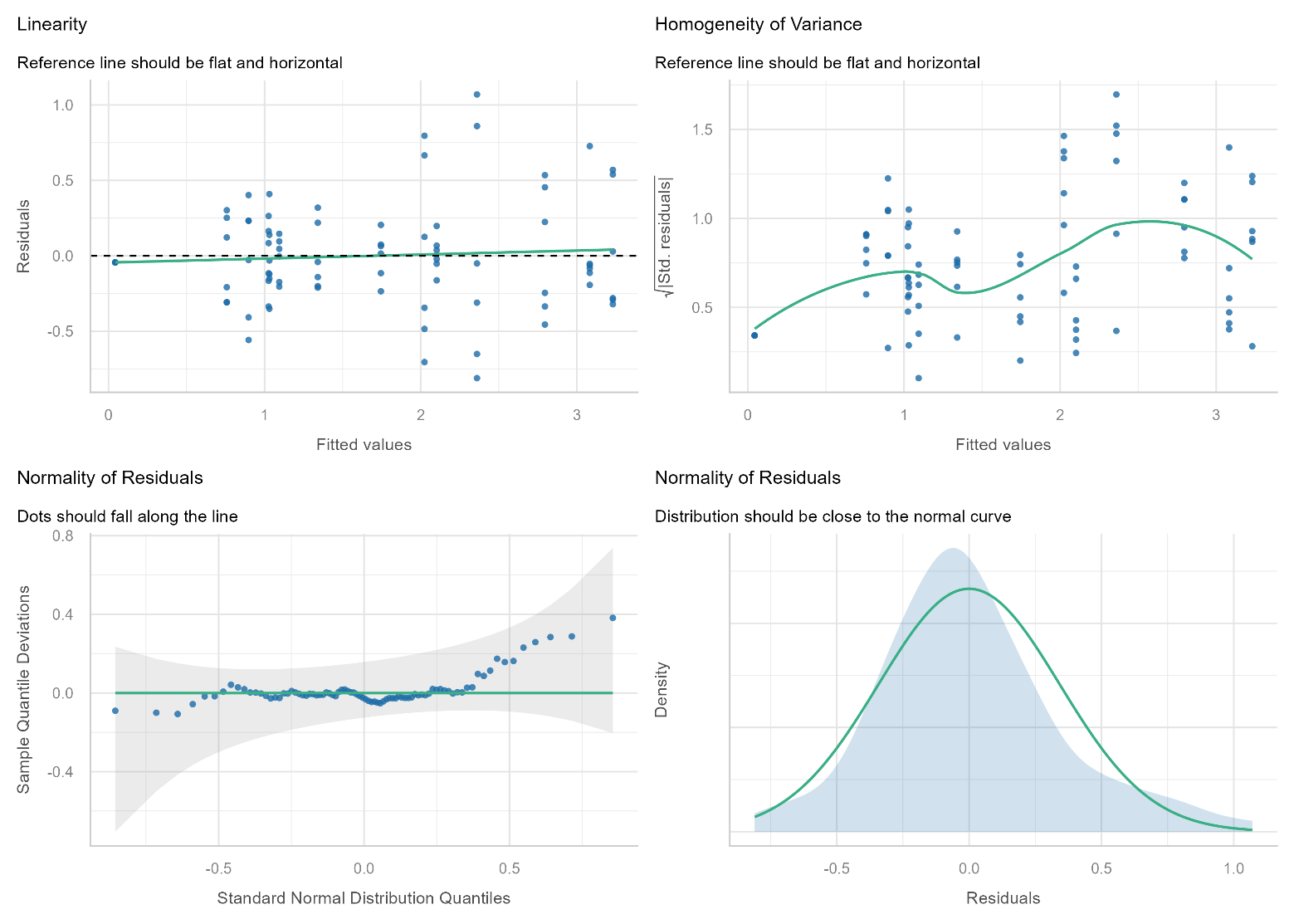


# Supplementary Figure 3

Residual diagnostic plots for Integration Score. Model: Score ~ 1 + (1|Mesh) + (1|Pig)


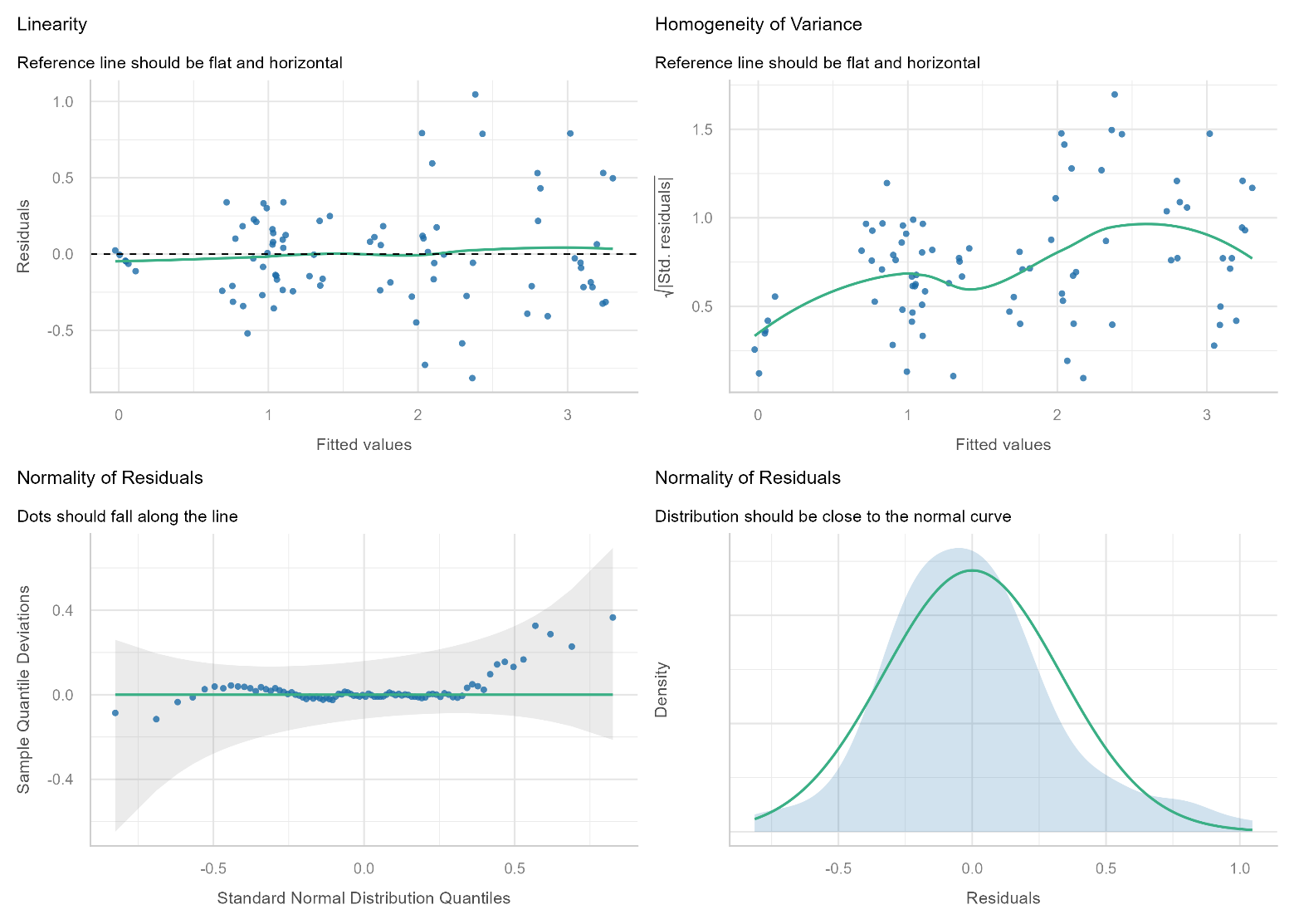


# Supplementary Figure 4

Residual diagnostic plots for Integration Score. Model: Score ~ Time + (1|Mesh) + (1|Pig)


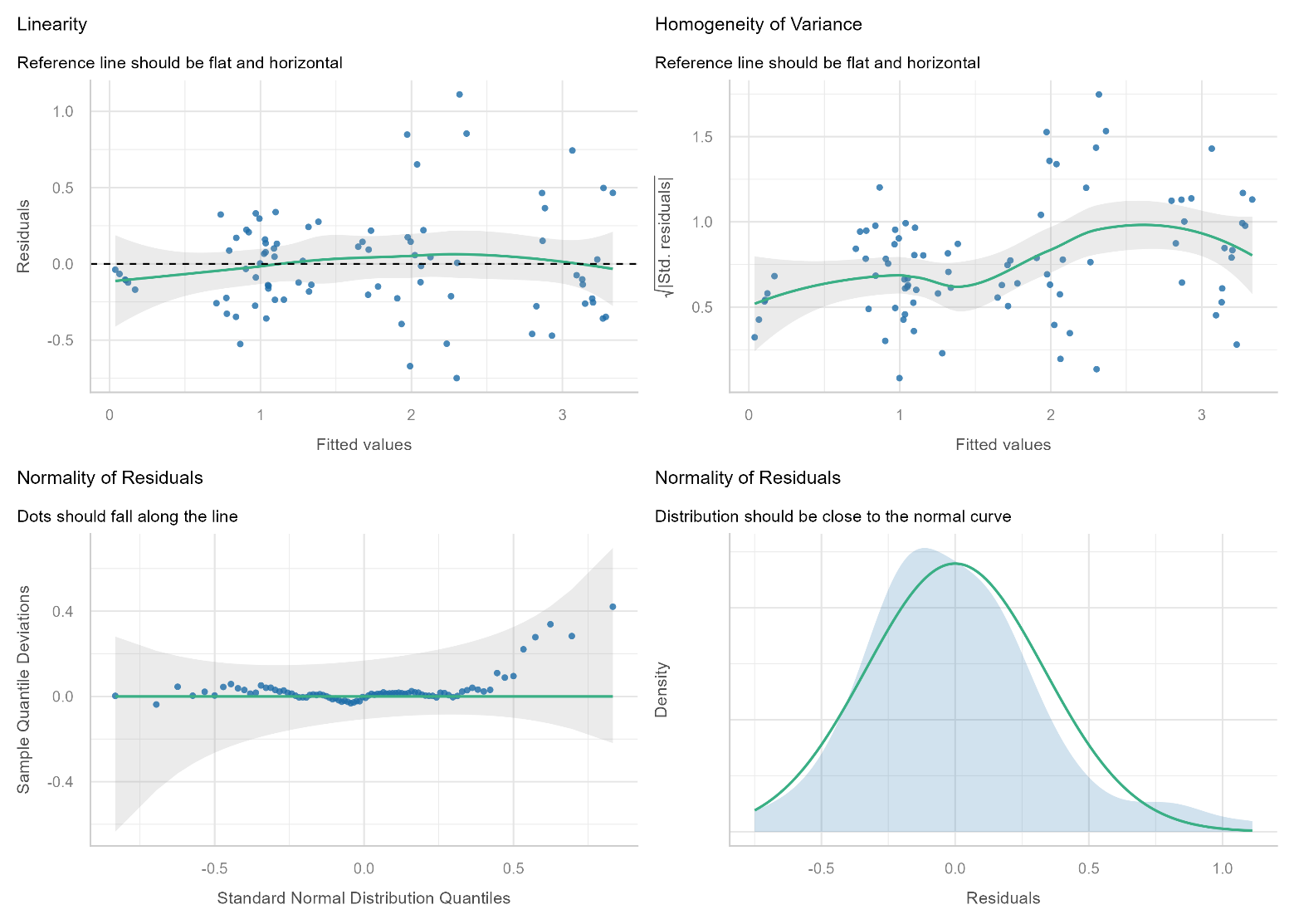


# Supplementary Figure 5

Residual diagnostic plots for Integration Score. Model: Score ~ poly(Time, 2) + (1|Mesh) + (1|Pig)


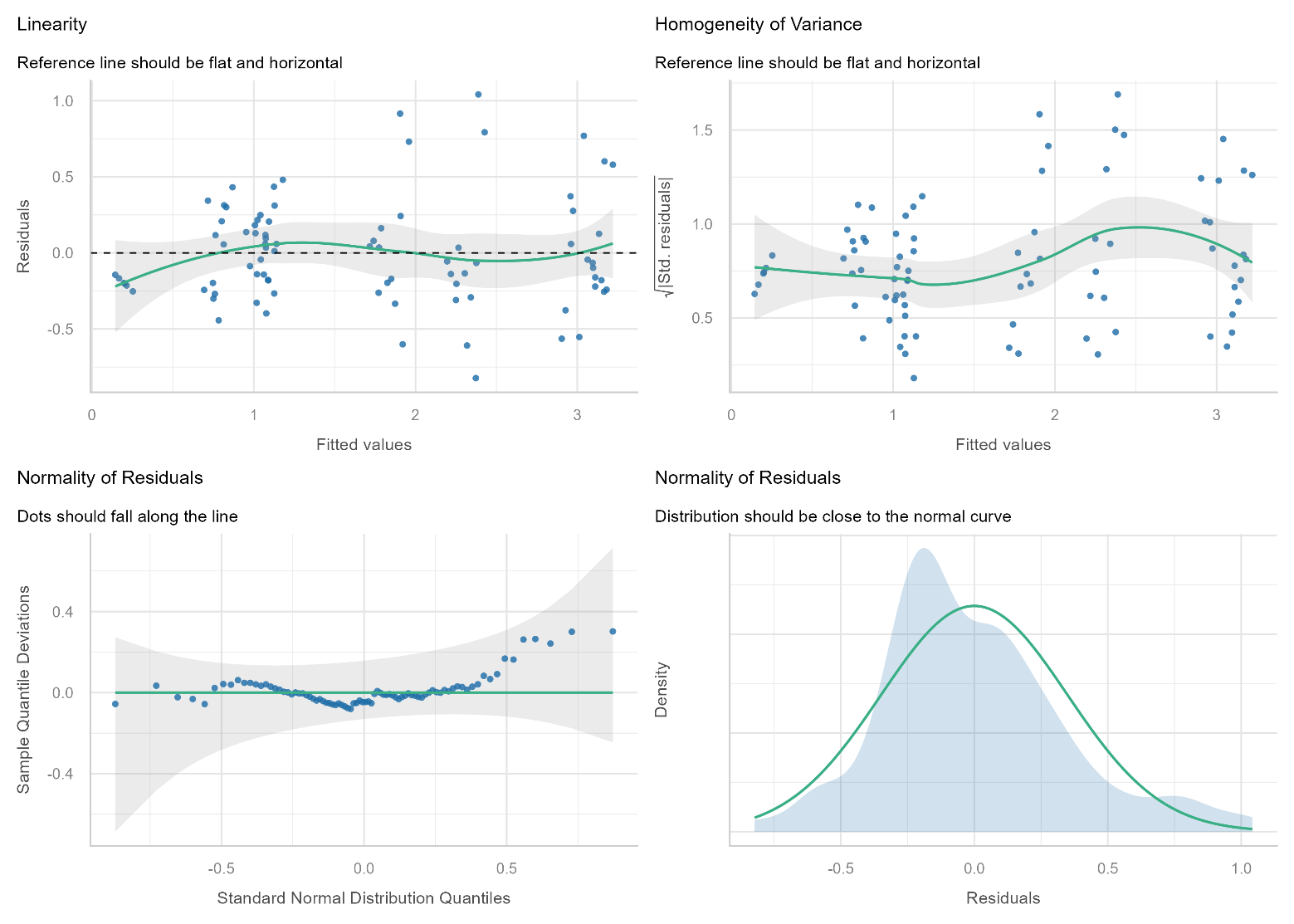


# Supplementary Figure 6

Residual diagnostic plots for Integration Score. Model: Score ~ poly(Time, 3) + (1|Mesh) + (1|Pig)


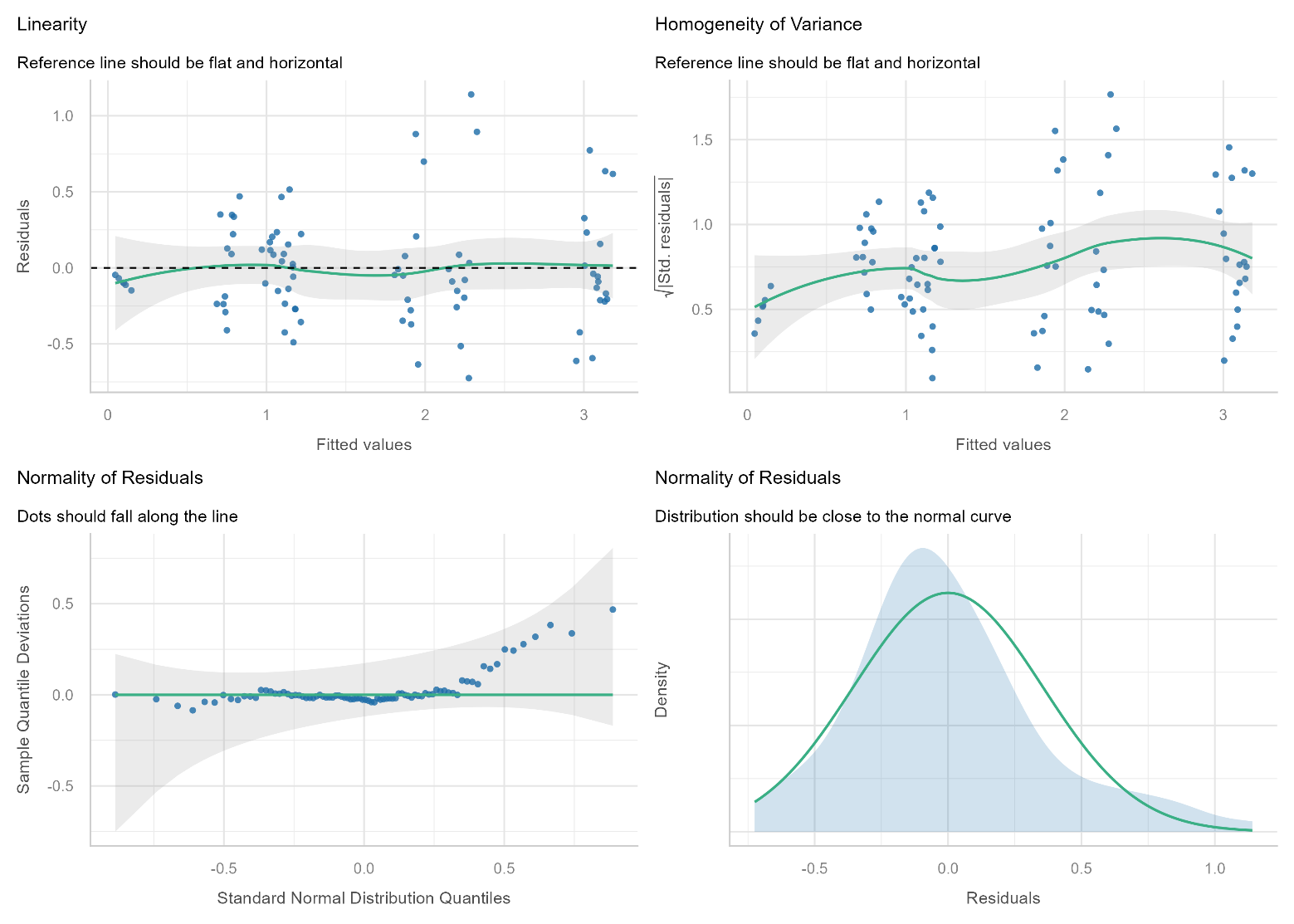


# Supplementary Figure 7

Residual diagnostic plots for Integration Score. Model: Score ~ poly(Time, 4) + (1|Mesh) + (1|Pig)


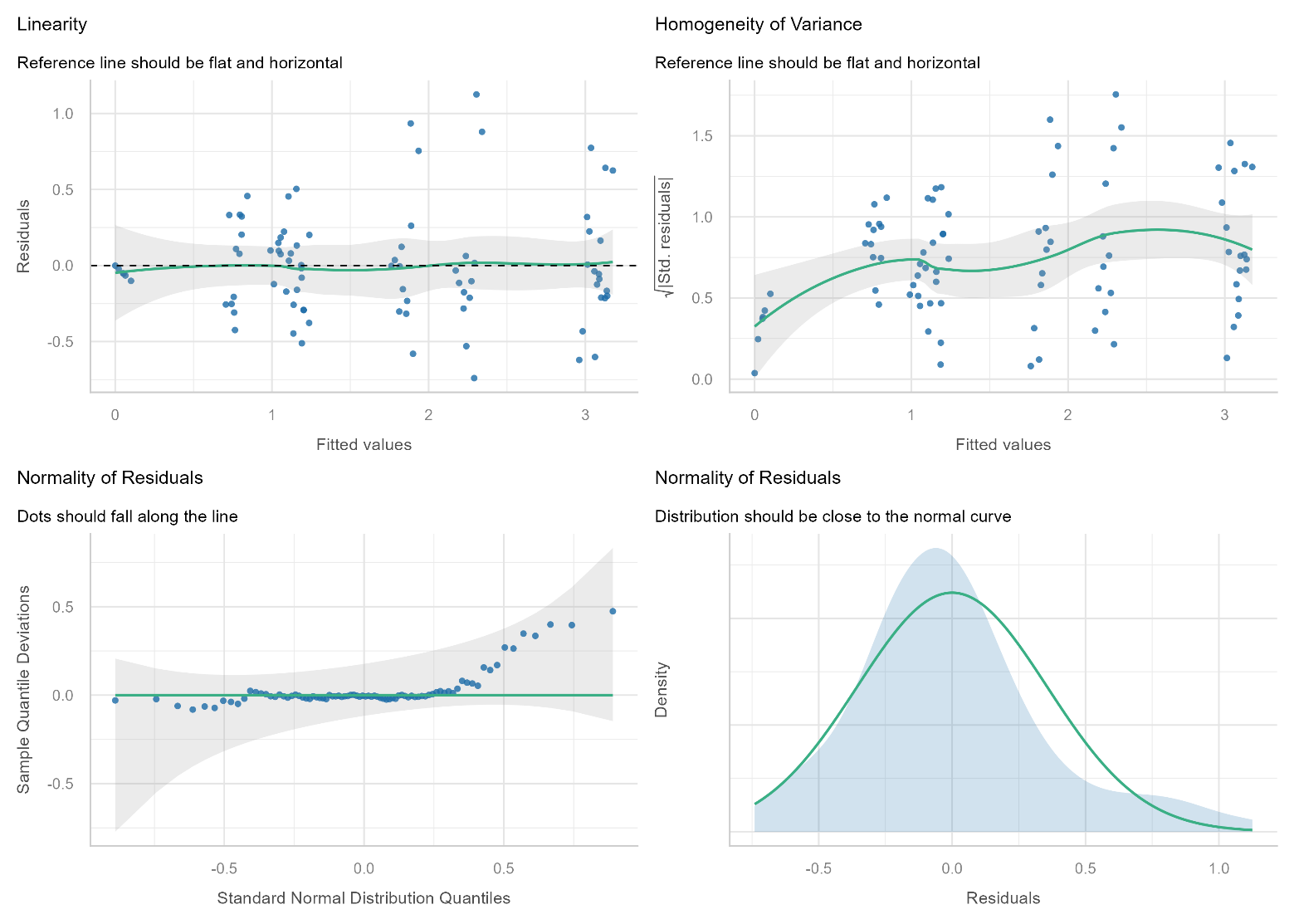


# Supplementary Figure 8

Residual diagnostic plots for Integration Score. Model: Score ~ SSlogis(Weeks, Asym, xmid, scal) ~ (Asym|Mesh) + (xmid|Pig)


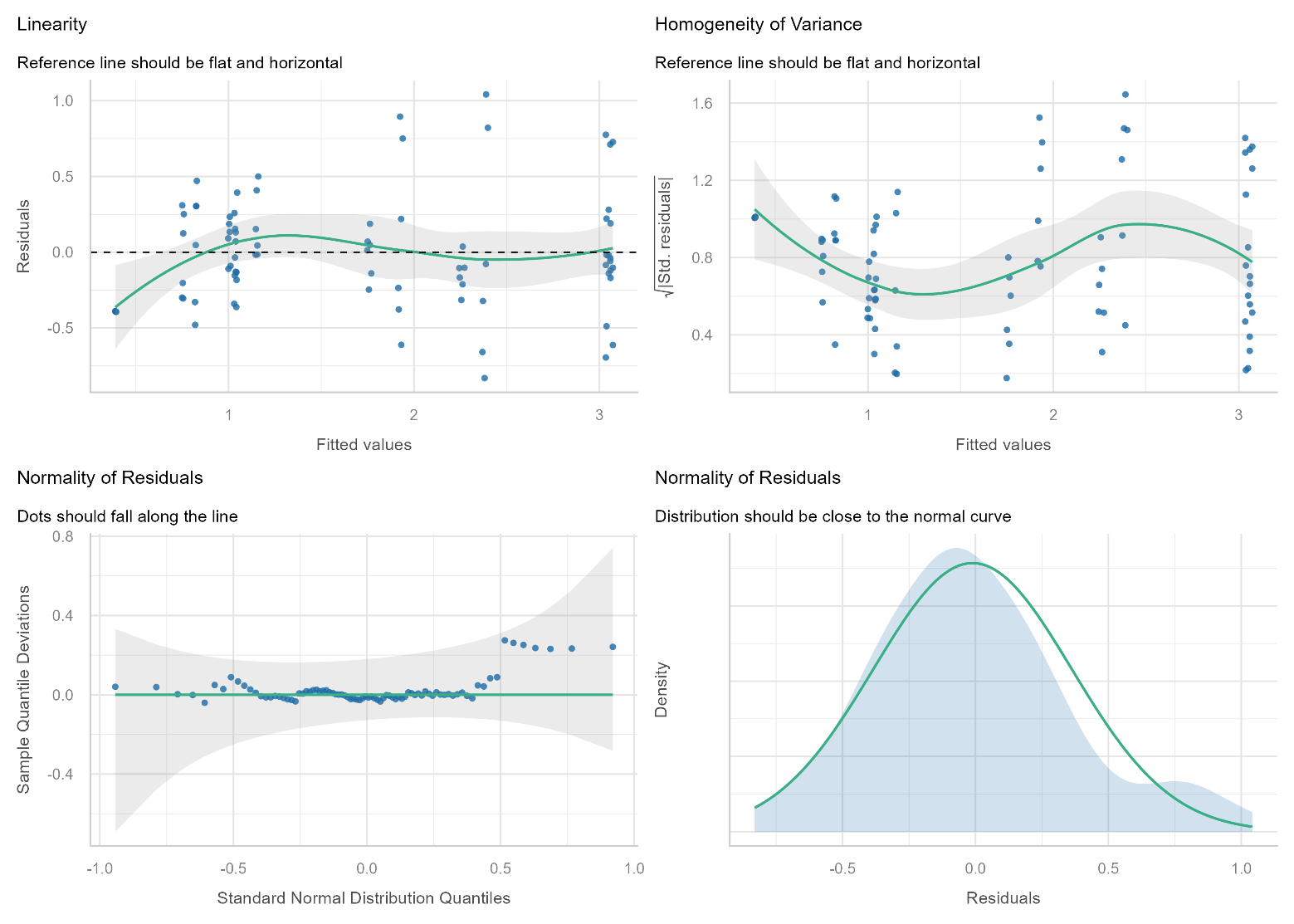


# Supplementary Figure 9

Residual diagnostic plots for Integration Score. Model: Score ~ SSasymp(Weeks, Asym, R0, lrc) ~ (Asym|Mesh) + (R0|Pig)


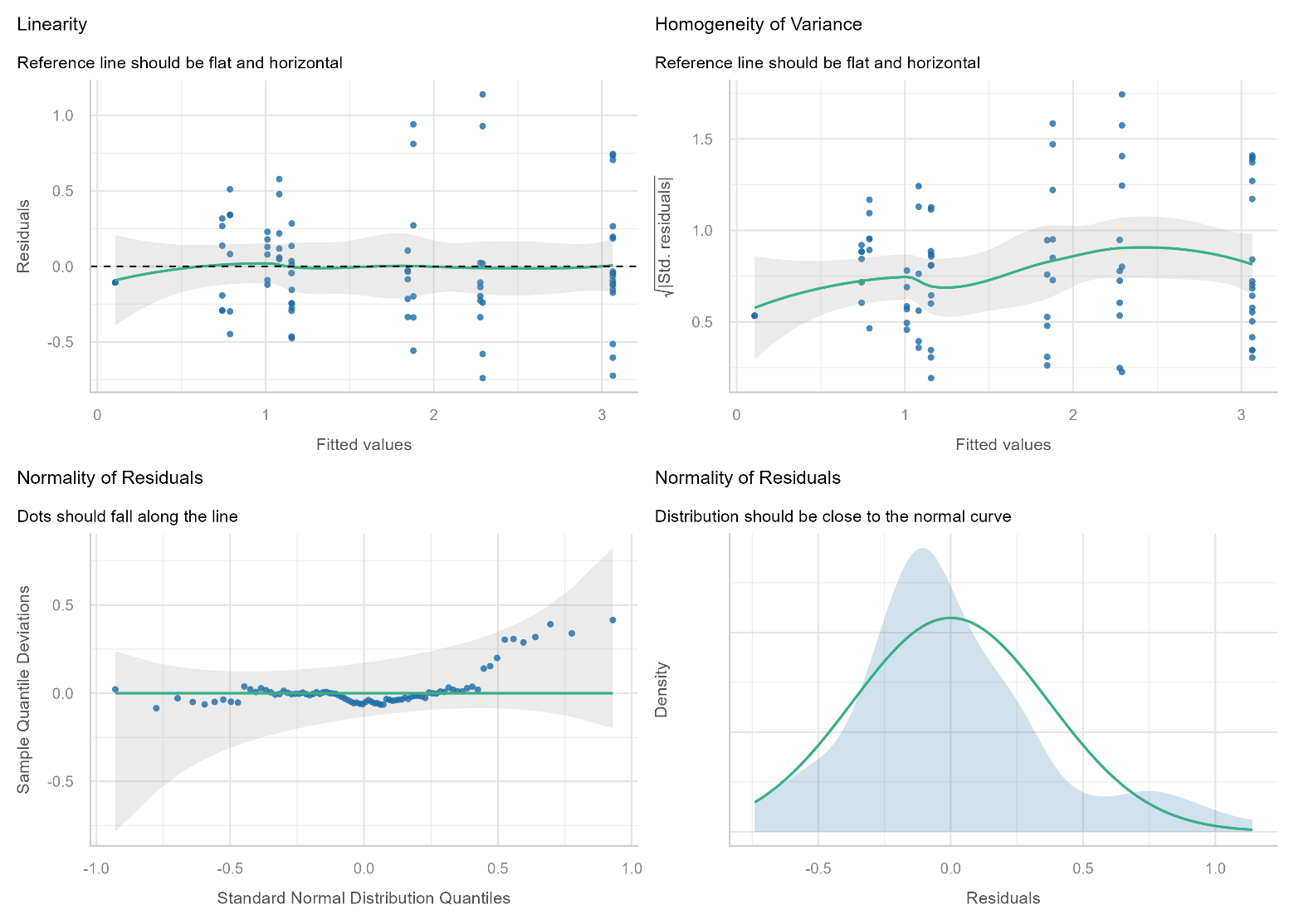


# Supplementary Figure 10

Residual diagnostic plots for Fibrosis Score. Model: Score ~ 1 + (1|Pig)


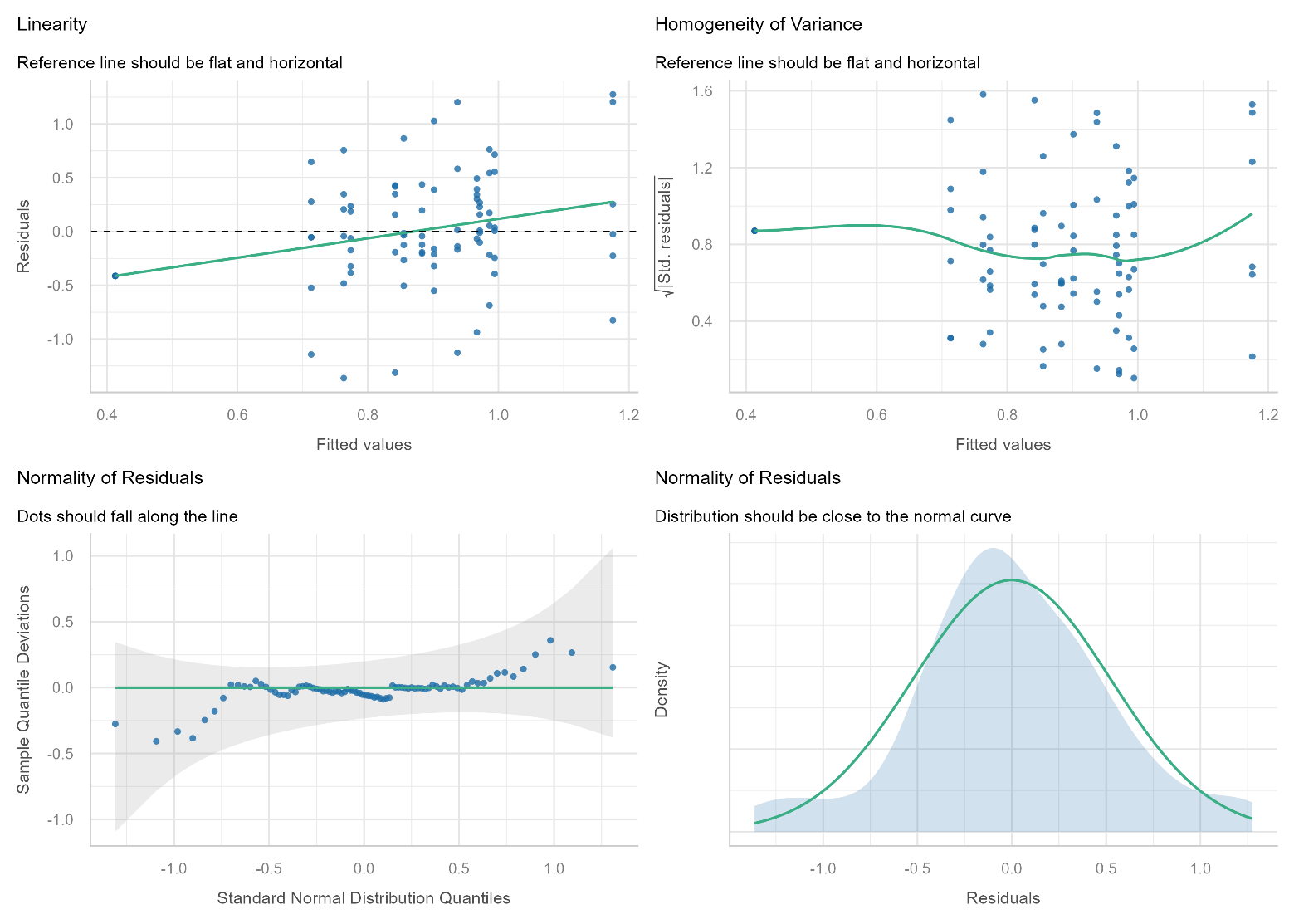


# Supplementary Figure 11

Residual diagnostic plots for Fibrosis Score. Model: Score ~ 1 + (1|Mesh) + (1|Pig)


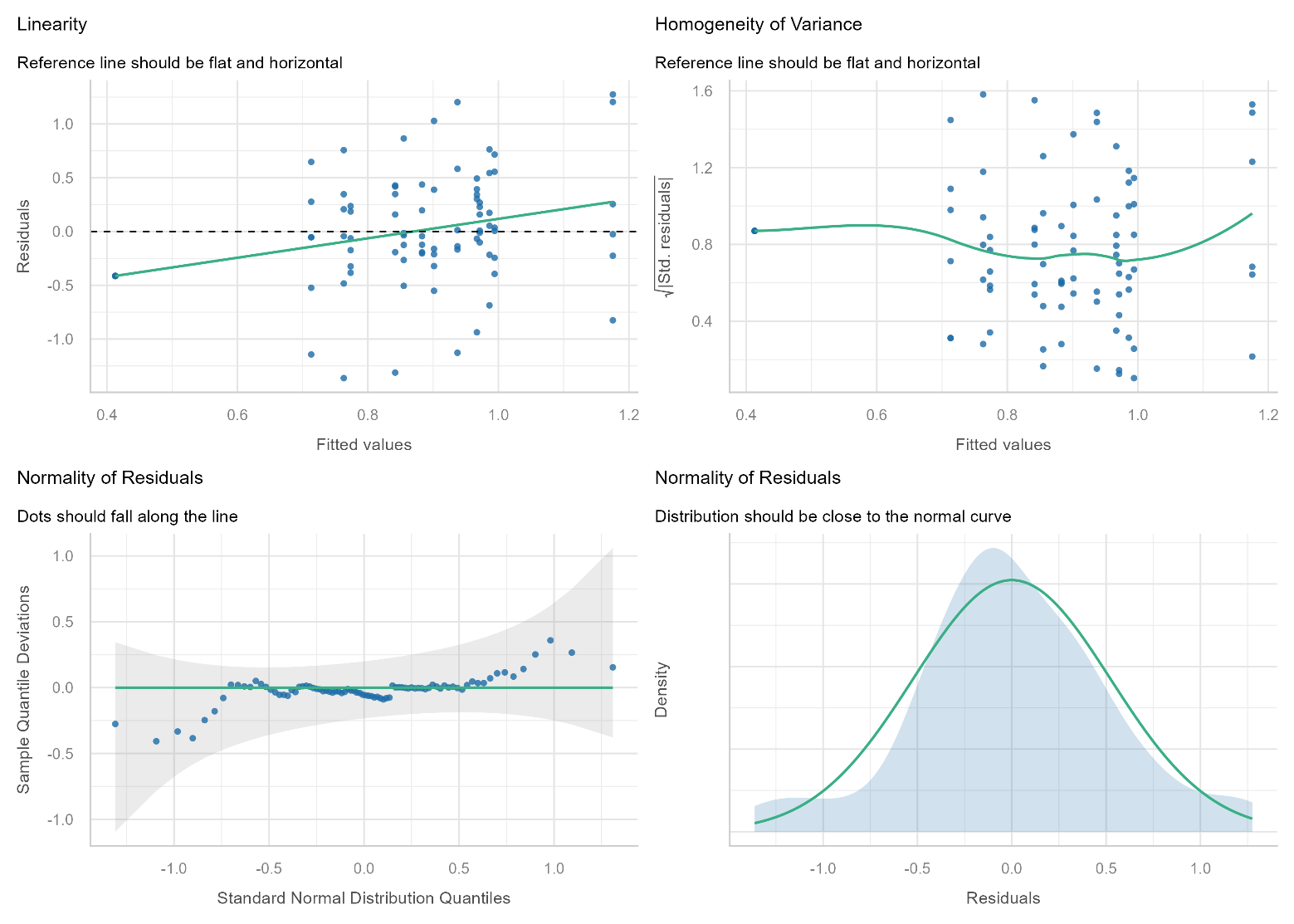


# Supplementary Figure 12

Residual diagnostic plots for Fibrosis Score. Model: Score ~ Time + (1|Mesh) + (1|Pig)


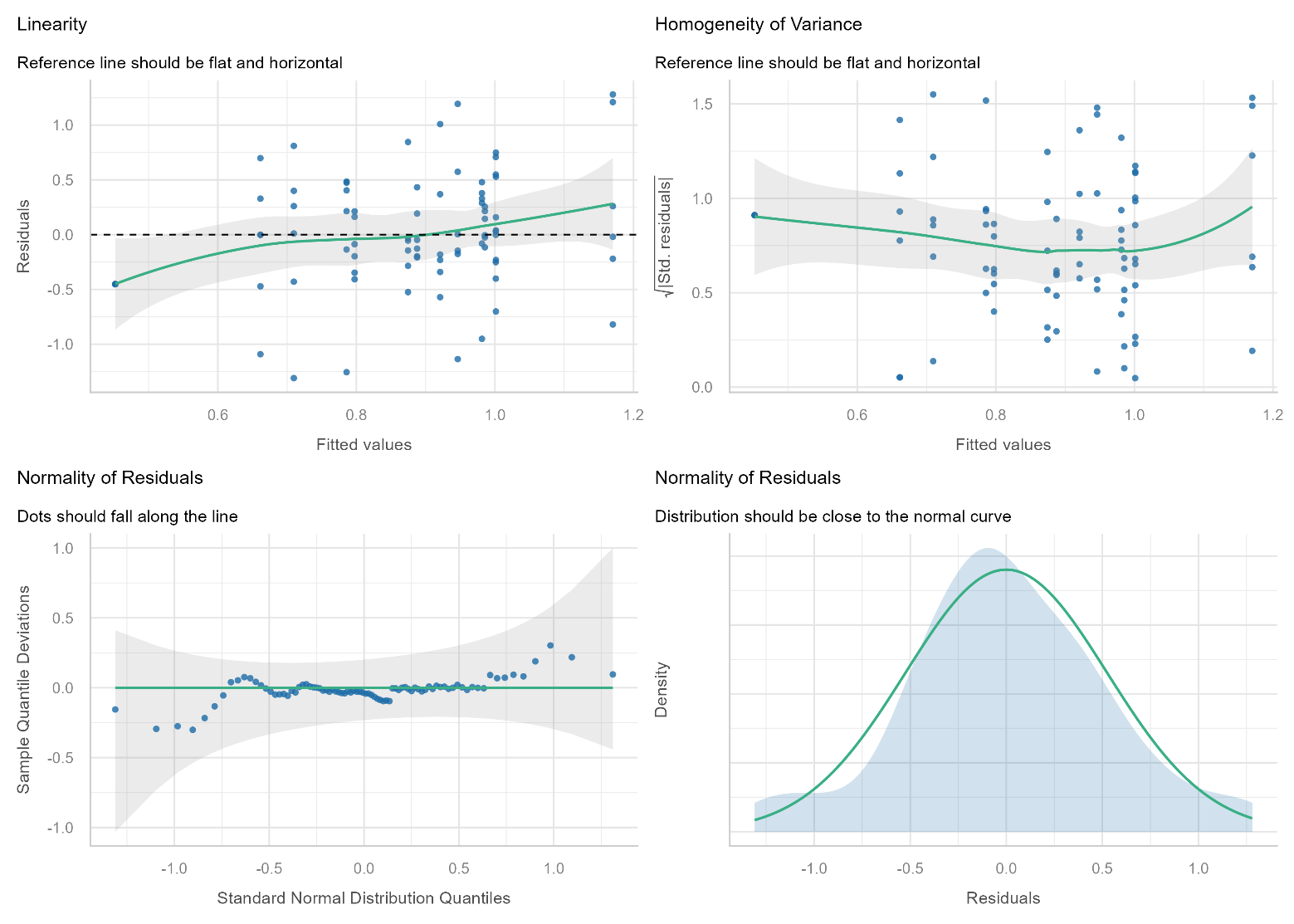


# Supplementary Figure 13

Residual diagnostic plots for Fibrosis Score. Model: Score ~ poly(Time, 2) + (1|Mesh) + (1|Pig)


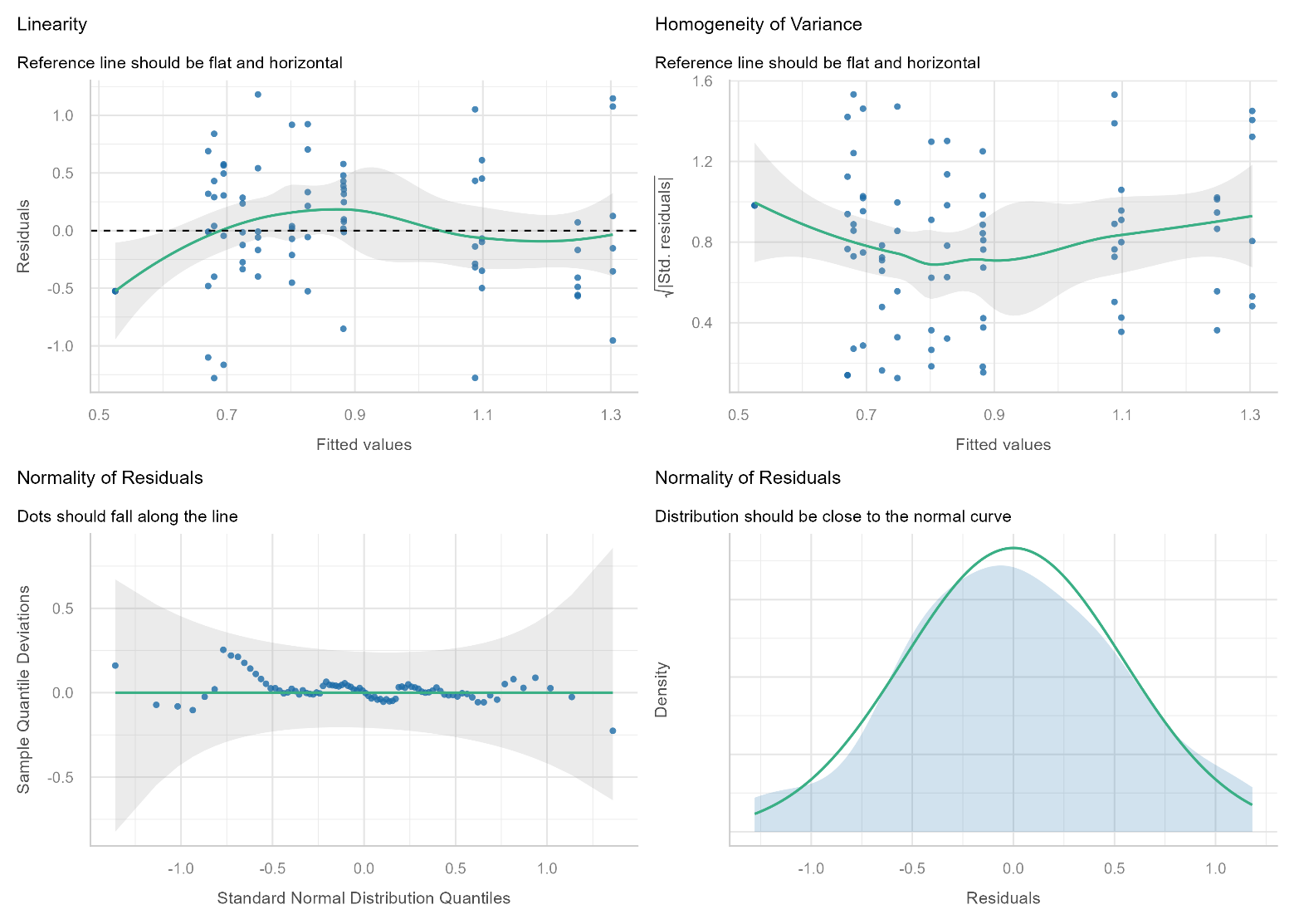


# Supplementary Figure 14

Residual diagnostic plots for Fibrosis Score. Model: Score ~ poly(Time, 3) + (1|Mesh) + (1|Pig)


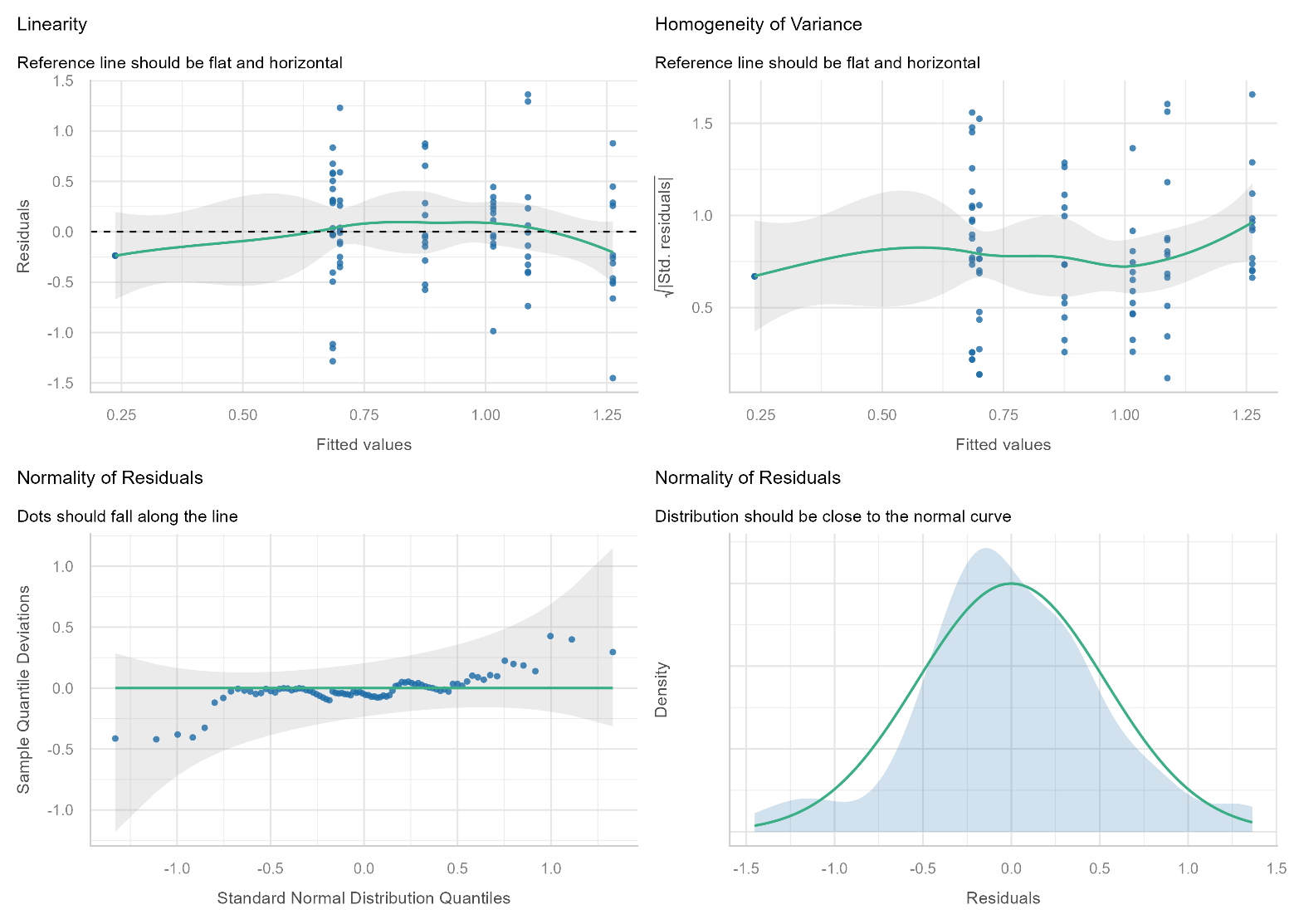


# Supplementary Figure 15

Residual diagnostic plots for Fibrosis Score. Model: Score ~ poly(Time, 4) + (1|Mesh) + (1|Pig)


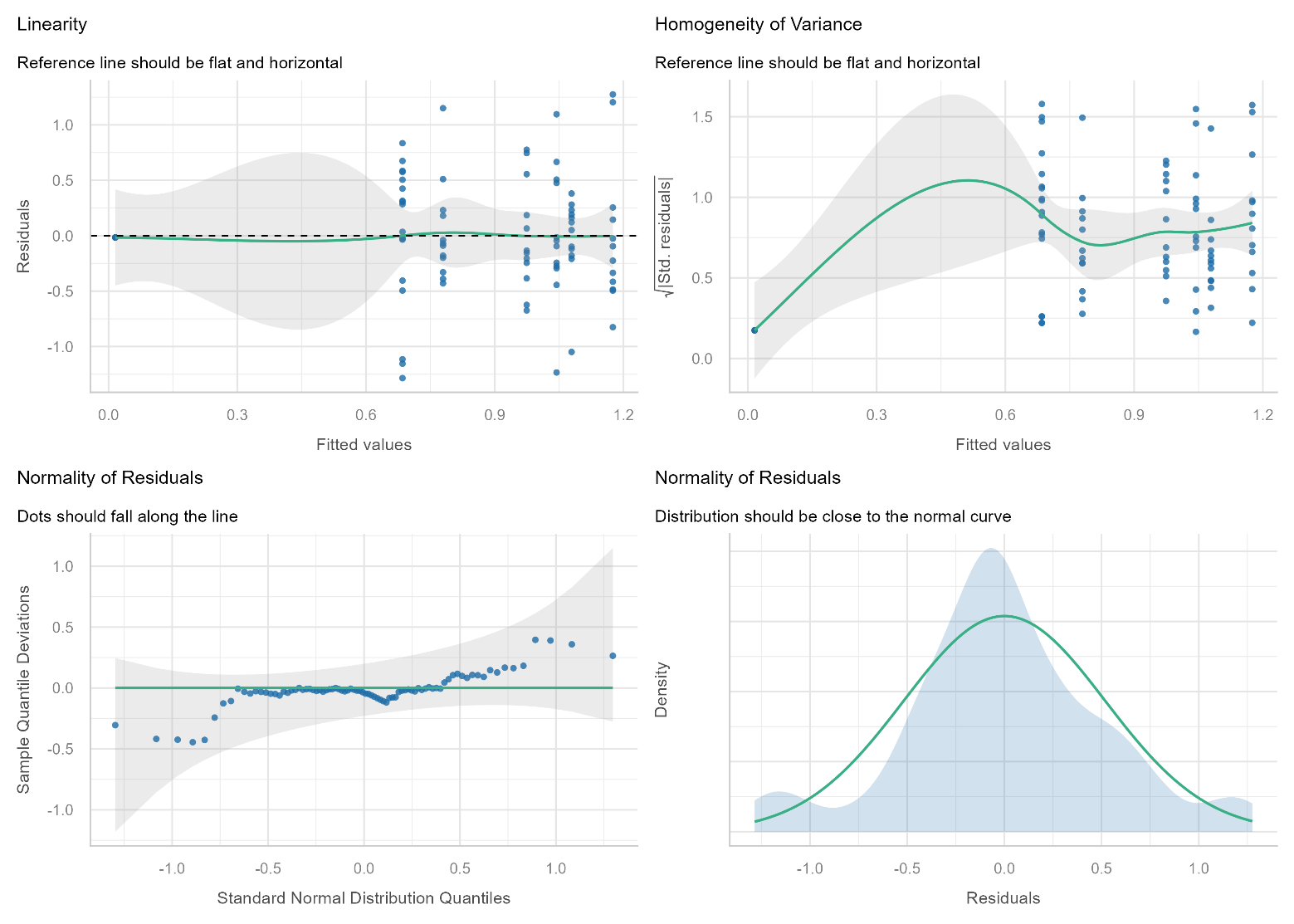


# Supplementary Figure 16

Residual diagnostic plots for Fibrosis Score. Model: Score ~ SSasymp(Weeks, Asym, R0, lrc) ~ (Asym|Mesh) + (R0|Pig)


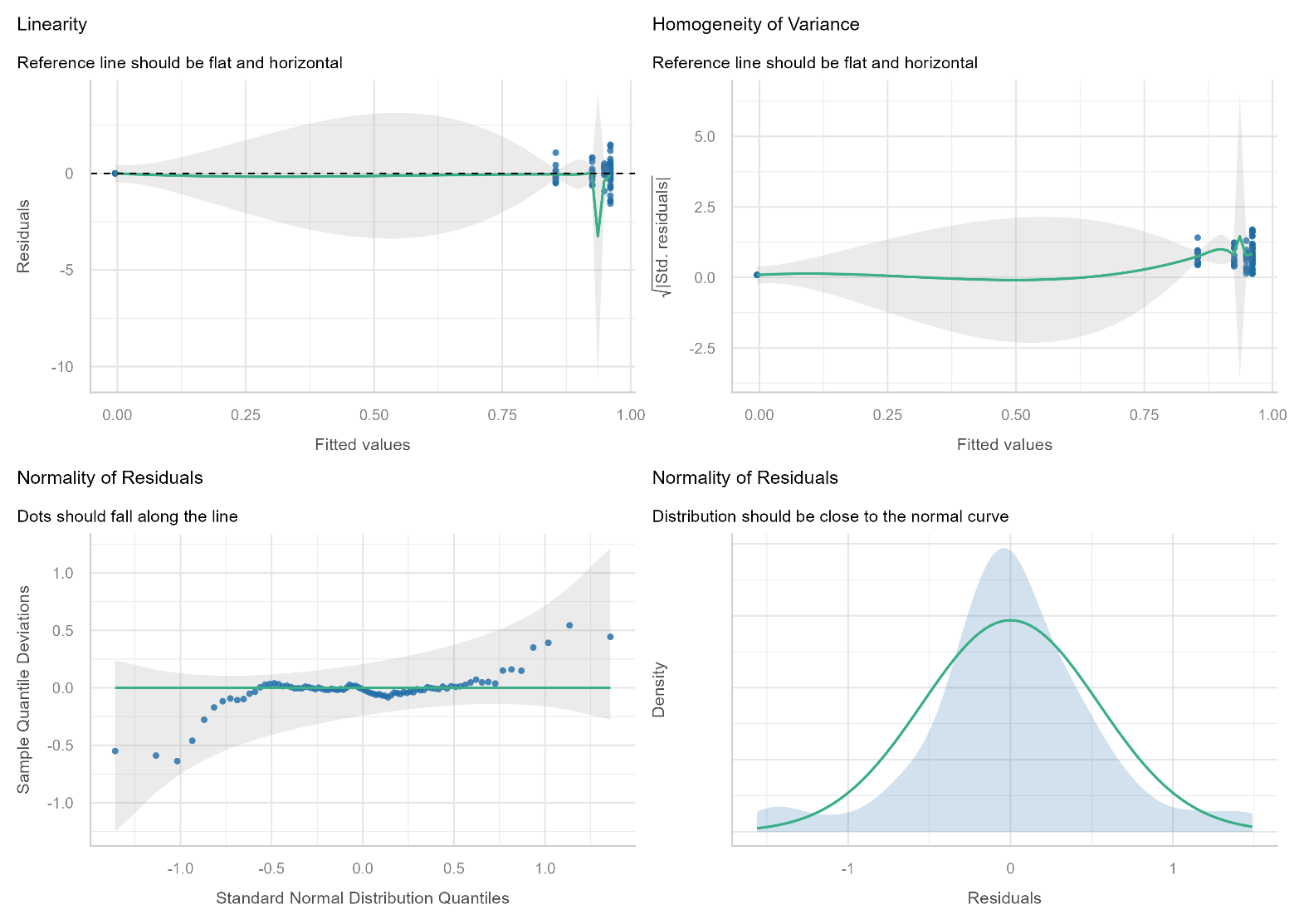


# Supplementary Figure 17

Residual diagnostic plots for Fibrosis Score. Model: Score ~ SSbiexp(Time, A1, lrc1, A2, lrc2) ~ (A1|Mesh) + (A2|Pig)


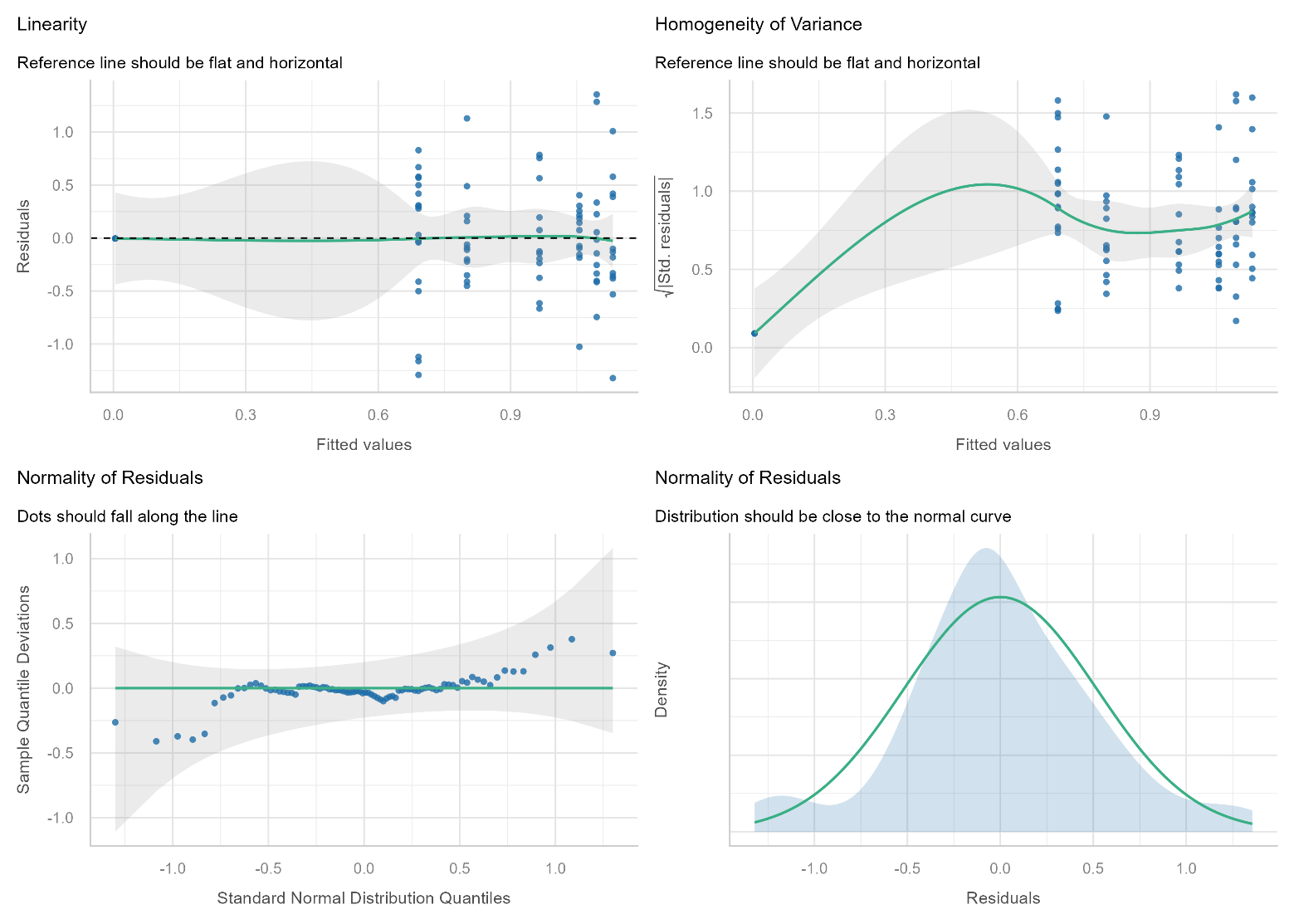


# Supplementary Figure 18

Residual diagnostic plots for Degradation Score. Model: Score ~ 1 + (1|Pig)


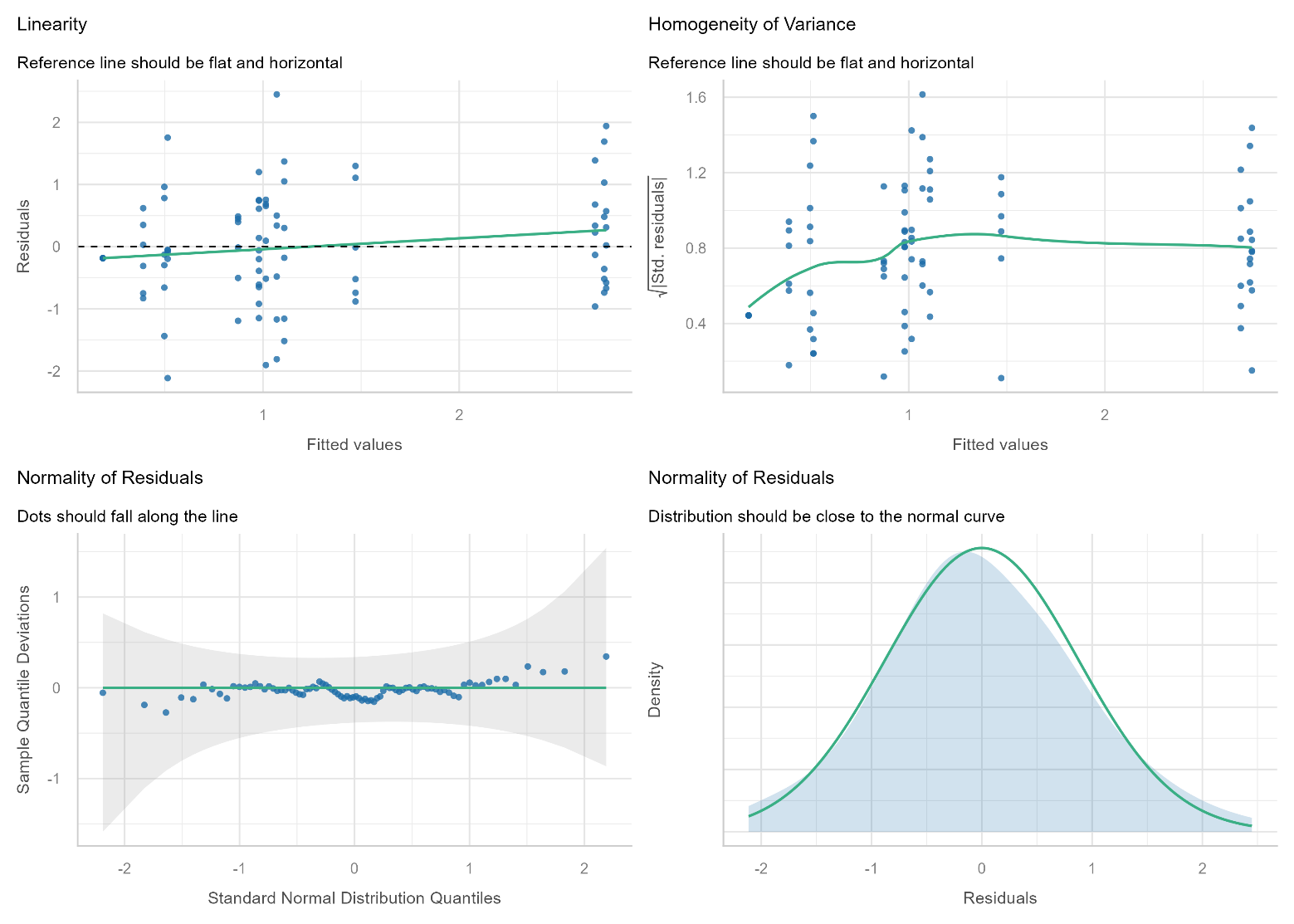


# Supplementary Figure 19

Residual diagnostic plots for Degradation Score. Model: Score ~ 1 + (1|Mesh) + (1|Pig)


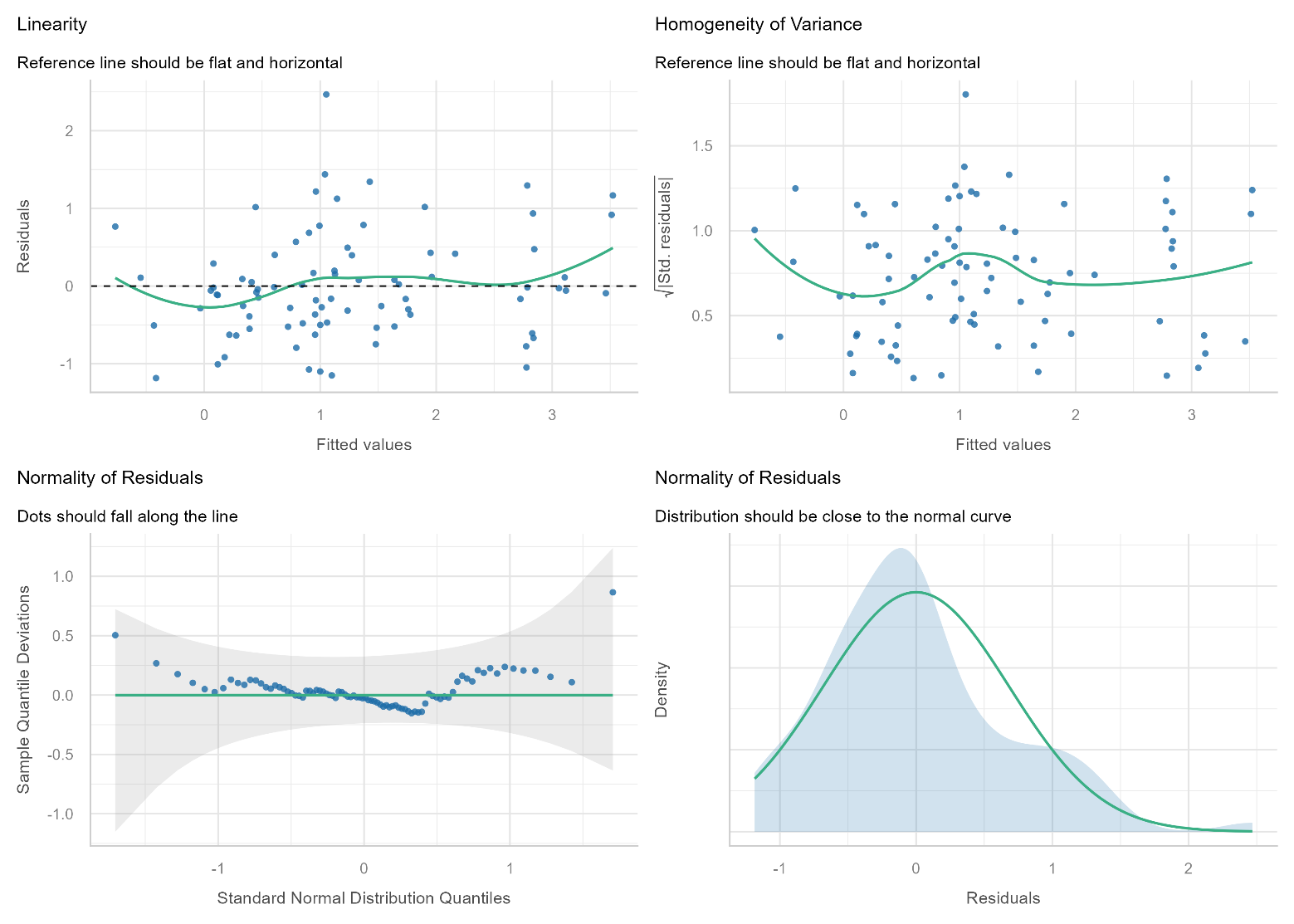


# Supplementary Figure 20

Residual diagnostic plots for Degradation Score. Model: Score ~ Time + (1|Mesh) + (1|Pig)


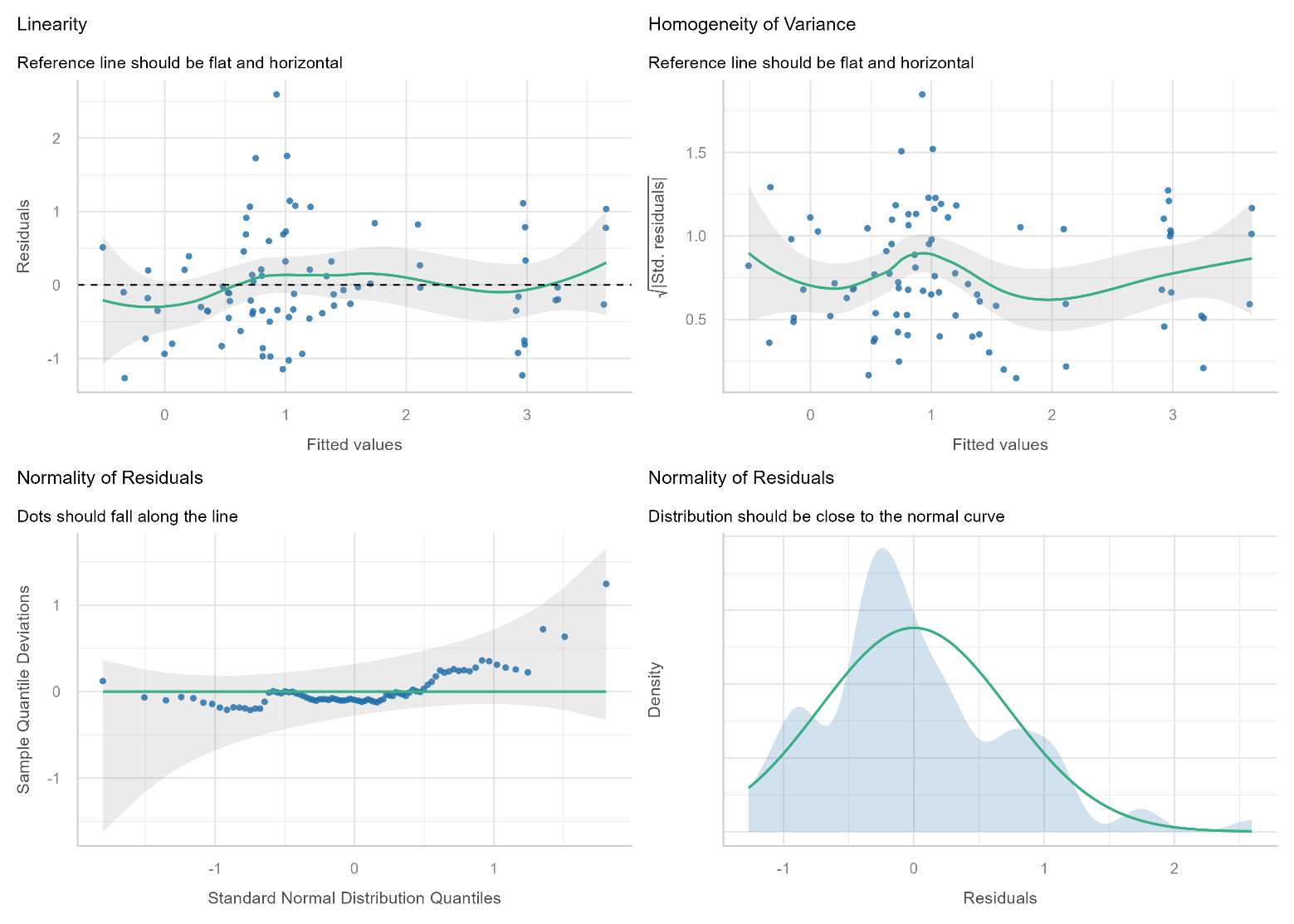


# Supplementary Figure 21

Residual diagnostic plots for Degradation Score. Model: Score ~ poly(Time, 2) + (1|Mesh) + (1|Pig)


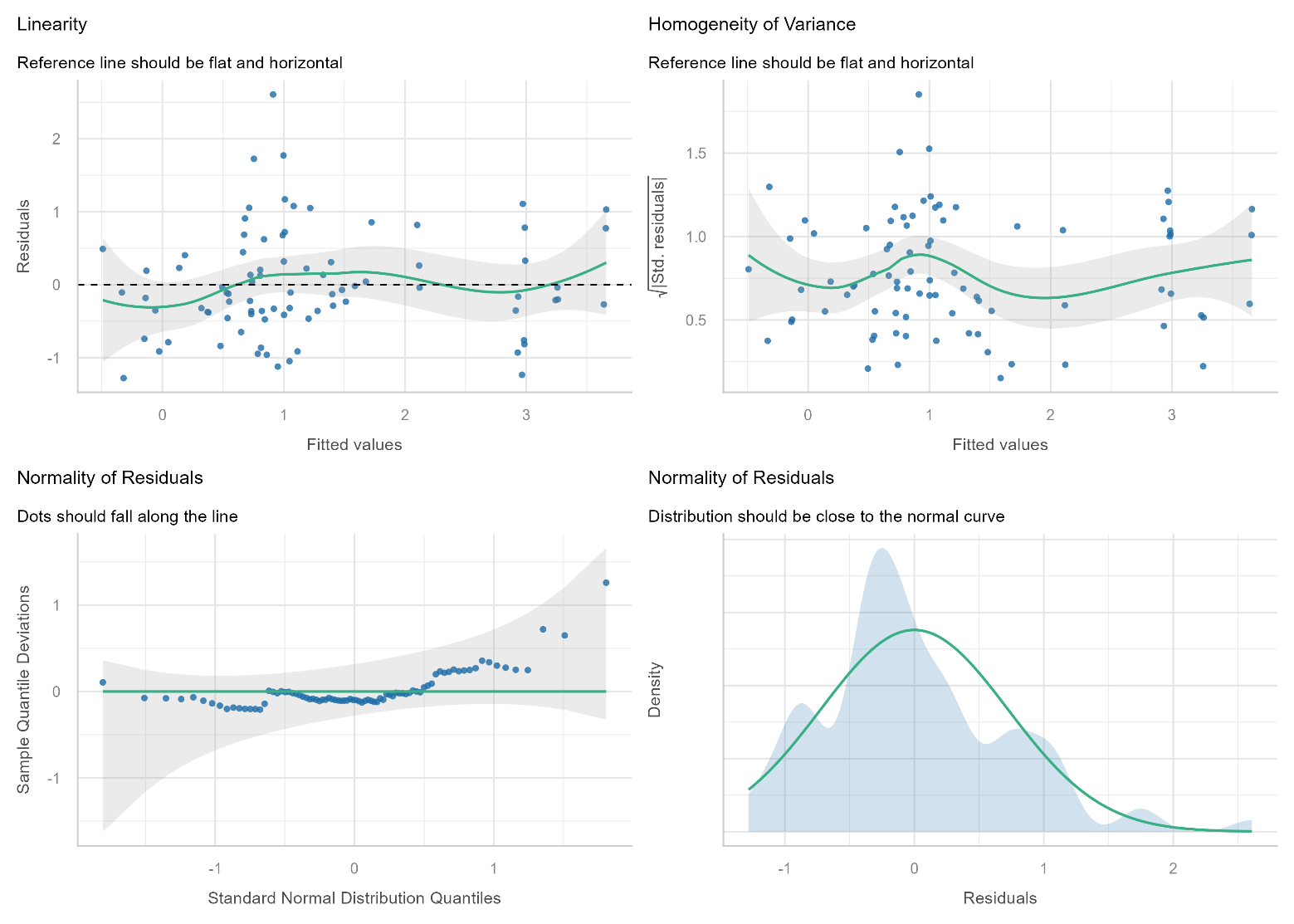


# Supplementary Figure 22

Residual diagnostic plots for Degradation Score. Model: Score ~ poly(Time, 3) + (1|Mesh) + (1|Pig)


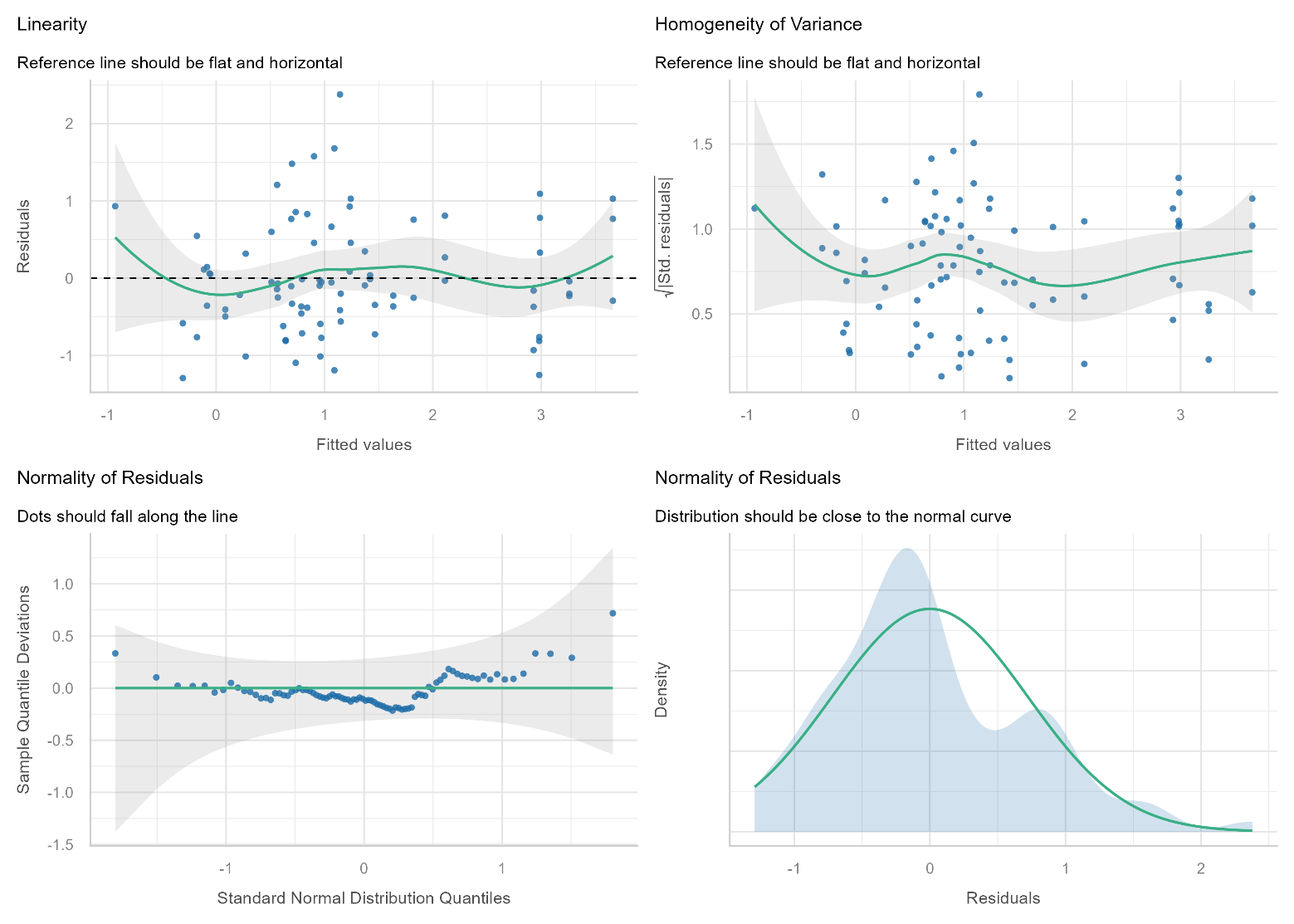


# Supplementary Figure 23

Residual diagnostic plots for Degradation Score. Model: Score ~ poly(Time, 4) + (1|Mesh) + (1|Pig)


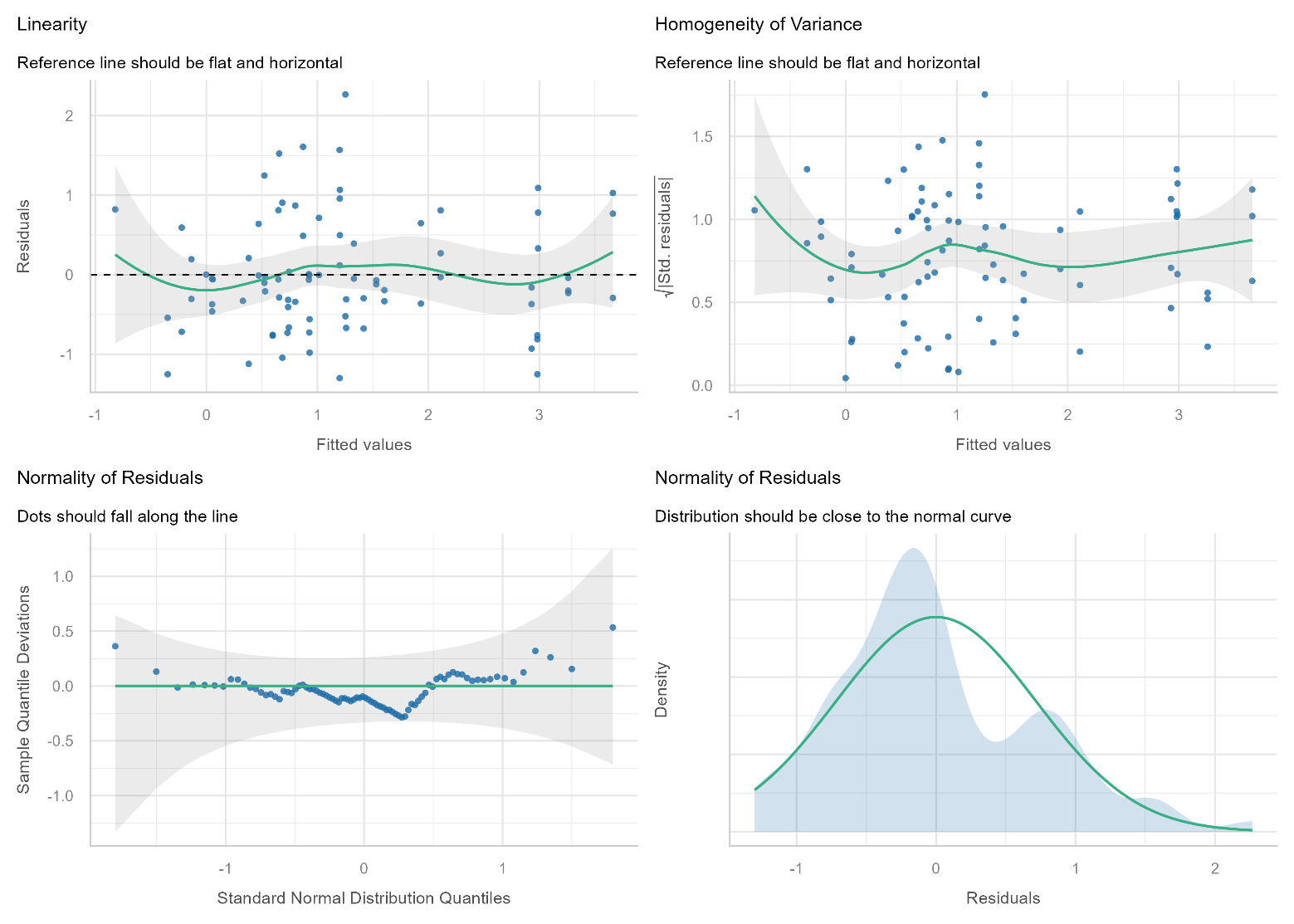


# Supplementary Figure 24

Residual diagnostic plots for Degradation Score. Model: Score ~ SSlogis(Weeks, Asym, xmid, scal) ~ (Asym|Mesh) + (xmid|Pig)


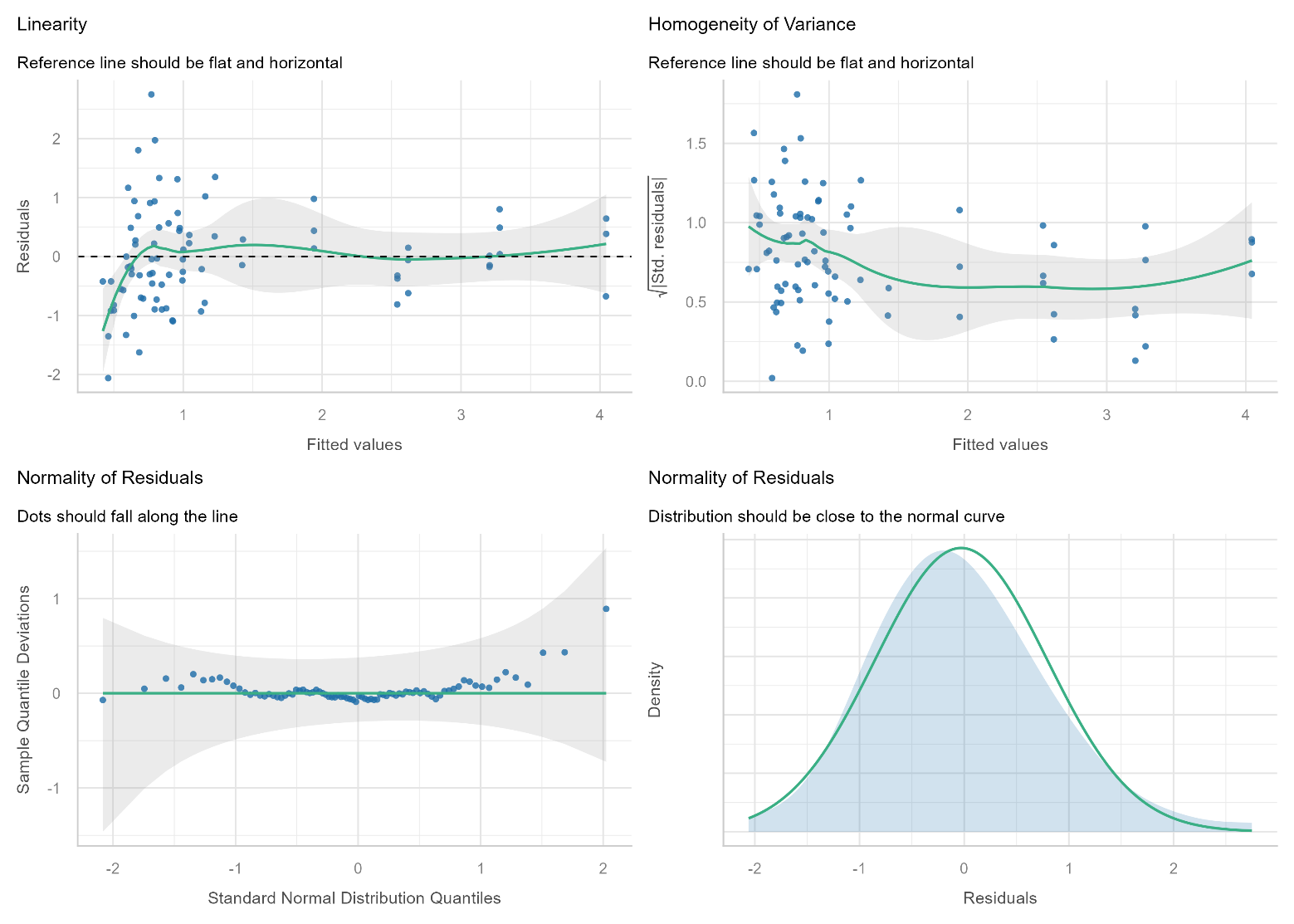


# Supplementary Figure 25

Residual diagnostic plots for Degradation Score. Model: Score ~ SSasymp(Weeks, Asym, R0, lrc) ~ (Asym|Mesh) + (R0|Pig)


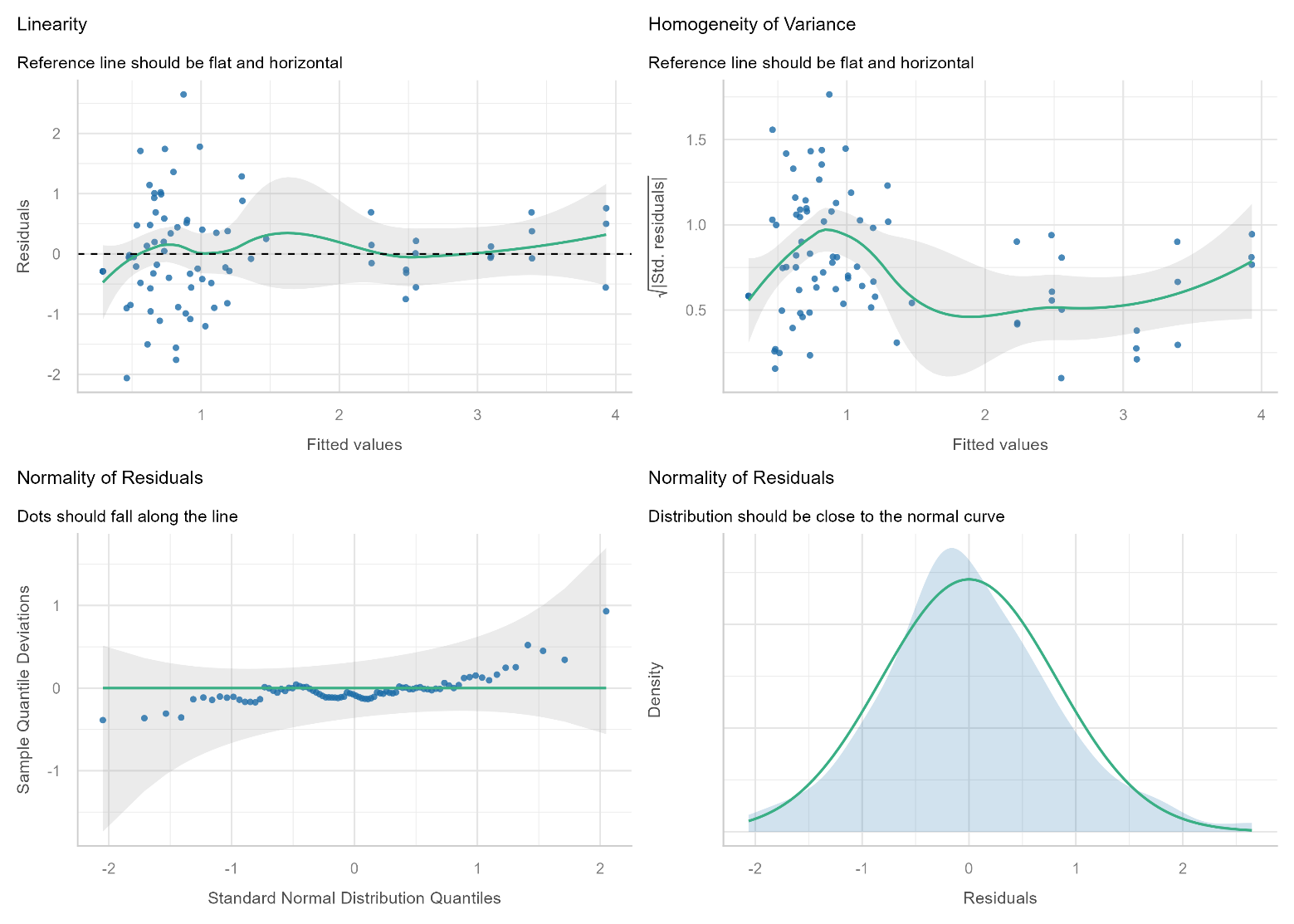


# Supplementary Figure 26

Hypothetical integration scores if biomechanical shear stress values are adjusted to account for double-sided tissue contact. Note the upshift of plots from original positions (Red Circles), to the adjusted values (Green Triangles)


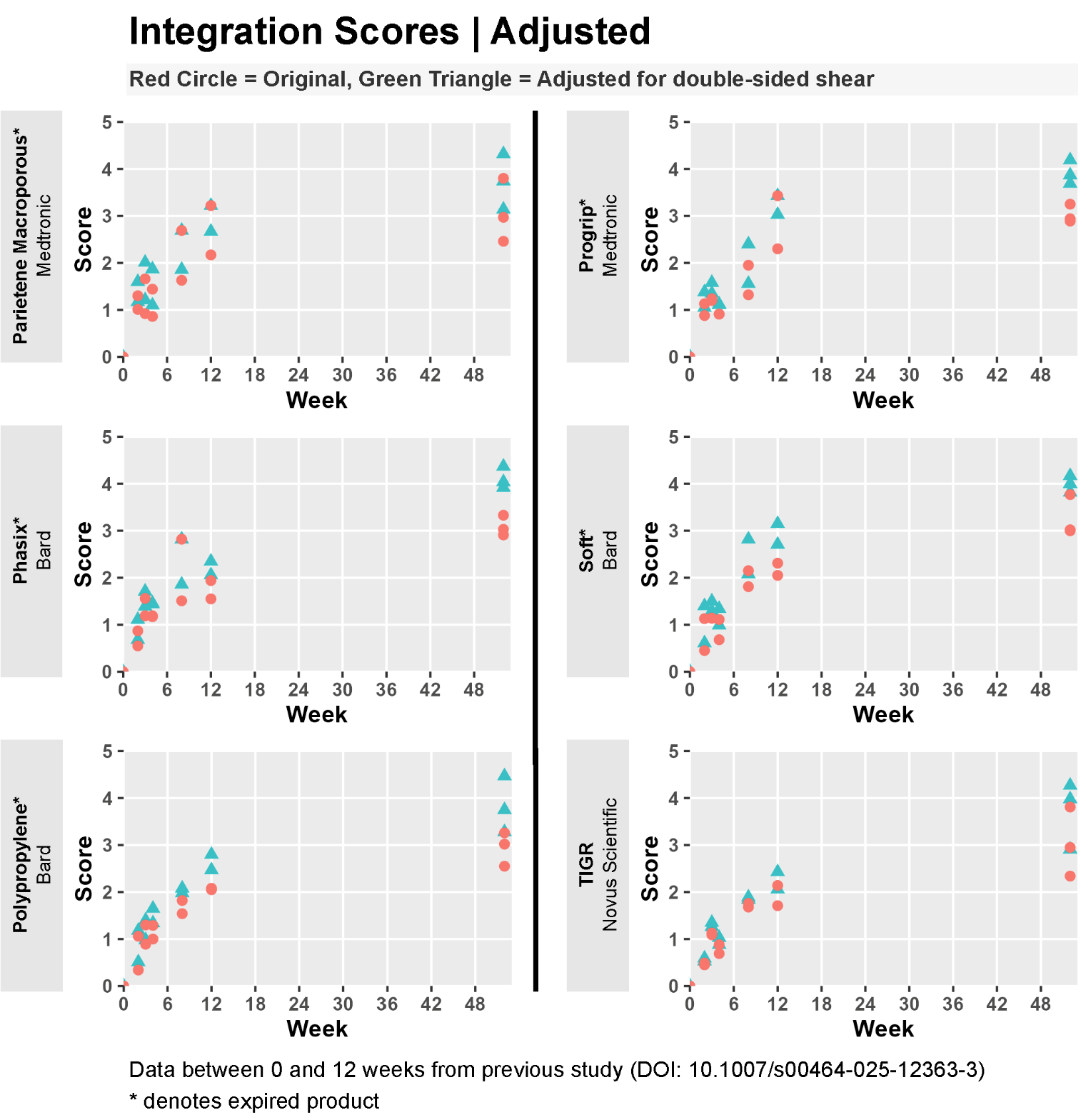

Supplement: Supplementary file 2 — Supplementary file2 (DOCX 8586 KB) [file 464_2026_12835_MOESM2_ESM.docx]
