# Supplementary material for: Extended validation of the mesh integration (MINT) index: a 1-year porcine study
Source: Surg Endosc. 2026 May 4;40(7):5917–32. doi: 10.1007/s00464-026-12835-0 (PMC13369702; doi:10.1007/s00464-026-12835-0)
Supplement: Supplementary file 3 — Supplementary file3 (PDF 60 KB) [file 464_2026_12835_MOESM3_ESM.pdf]

Preparation

Acquire pigs

- Landrace x Large White, 11-12 weeks, 30-35 kg
- Three in total
- Designated piggery per SAHMRI
- Standard pig chow per SAHMRI
- Water ad libitum
- Toys + enrichment
- All veterinarian care as per SAHMRI
- Engage animal welfare officer

Acquire consumables

- Standard stock as per SAHMRI + sterility
- Special stock to be pre-ordered

Acquire mesh

- Reuse mesh from same lot and batch from previous study (DOI 10.1007/s00464-025-12363-3)
- Need 6 pieces retrorectus

Prepare mesh

- Generate alphanumeric labels
- If sterile, in date and sufficiently small
  - Remove external packaging, leaving only the internal non-labelled sterile packet intact
  - Place packet in sterilisation bag without viewing window
  - Label bags
  - Use RNG to pick random packets for controls (Original)
  - Store until usage
- If non-sterile, expired or large
  - Cut to approx. 5x5 cm, using surgical scissors and ruler, under laminar air flow
  - Photograph all pieces during cutting with ruler + individual labels
  - Place each mesh into sterilisation bags
  - Label bags
  - Use RNG to pick random packets for controls (Original)
  - Sterilise via EO
  - Retrieve and air dry
  - Use RNG to pick random packets for controls (Sterilised)
  - Store until usage

Allocate mesh

- Remove all mesh assigned to Controls from RNG pool
- Use RNG to pick one packet for each mesh product
- Without duplicating meshes, every six packets form a set
- For each set, use RNG to assign mesh to location within indicated layer. Print diagram out.
- Seal each set with assigned diagram
- Label sets with number
- Store sets together until usage

Perioperative

Preop Day 7

- Acclimatise pigs to facility
- Administer any vaccines / medicines required by SAHMRI
- Visit pigs regularly
- Check facility availability
- Confirm operative time + expected personnel (need 3+)

Preop Day 3

- Veterinarian checks
- Use RNG to pick pig/s for operation, move to perioperative area

Preop Day 1 / 24 hours

- Naturally fast pigs after afternoon meal
- Apply analgesia patch

Day of operation

- Exchange analgesia patch for fresh one
- Weigh pig
- Assign pig code
- Pre-operative preparation / induction / anaesthesia as per SAHMRI
- Note time of induction / doses
- Supine, secured to operating table
- Shave, remove hair, disinfect
- Set up sterile field, open kits, open consumables
- Use RNG to pick mesh set to use, assign pig code to this set
- Display mesh allocation
- Scrub, drape, set up surgical field
- Note time of operation
- Midline entry, with careful penetration of layers
- When sufficient depth, dissect off midline into rectus
- Pass thorough rectus muscles to enter retrorectus space
- Develop space, elongate entry as necessary
- Repeat for contralateral side
- Open mesh packet, and retrieve mesh in sterile fashion (use assistant)
- Photograph mesh with ruler + label
- Place mesh to assigned spot, apply sutures at 9 points
- Repeat for all 6 retrorectus mesh
- Exit and close as per standard laparotomy
- Opsite spray to skin
- Note time of extubation
- Document operative note + any deviations
- Take copy of anaesthetic chart
- Repeat for next pig

Post-op care

- Extubate as per SAHMRI
- Return to recovery area + observations as per CRS chart (DOI 10.1007/s00464-025-12363-3)
- Abx, analgesia as per protocol
- Daily ward round
- Return to general stay area once CRS scores back to baseline + nil other concerns
- Standard care and regular visits until post-mortem

Post-mortem

- Book post-mortem dates
- Confirm equipment
- RNG not required as same end point for all pigs
- Euthanasia via terminal anaesthesia by SAHMRI
- Death certification by SAHMRI
- Move to theatre
- Position supine, secure to table
- Shave, remove hair, disinfect
- Set up sterile field, open kits, open consumables
- Display mesh allocation on board
- Lower pelvic incision, with identification of layers
- Tunnel along linea semilunaris and locate far lateral edges of all 6 meshes
- Separate posterior rectus sheath from each mesh
- Photograph mesh with ruler and label
- Take control histology samples from retrorectus layers
- Isolate each mesh and remove en bloc
- Cut to size per protocol (DOI 10.1007/s00464-025-12363-3)
    - Centre piece / histology in 10% formalin
    - Remaining tissue in 0.9% phosphate buffered saline, submerged in 4°C ice bath
- Transport
    - Fresh tissue to biomedical labs (priority)
    - Formalin samples to histology labs

Post-mortem Day 0

Biomechanical Assessment

- Ensure tensiometer is calibrated
- Ensure correct load cell is installed
- Setup up working space
- Perform shear testing, as per DOI: 10.1007/s00464-025-12098-1
- Record
    - sample contact length
    - sample contact width
    - stress/strain graph

Protease Processing

- Place pieces intended for SEM / FTIR or control mesh into individual test tubes + labels
- Fill tests tubes with 40 ml 0.1M $NaHCO_3$ + 10 ml Alcalase®
- Incubate at 58°C for 24 hours

Post-mortem Day 1-2

Histology Assessment

- Embed in paraffin
- Section to 5 μm
- Stain with H&E, Masson's trichrome

Protease Processing

- At 24 hours, remove samples
- Rinse samples with DW thoroughly
- Air dry
- Use ATR-FTIR, to check absence of amide peak at 1630-1700 cm^{-1} (see below)
- Store in clean test tube when not in use

Molecular Assessment

- Setup ATR-FTIR with diamond prism
- Follow prompt, and record background spectra
- Take spectra from three random spots on each sample
- Record and process spectra in compatible software
- Measure
    - Area under curve for all spectra samples

Biomechanical Assessment

- Setup up working space

- Perform tensile testing of mesh, as per previous (DOI: 10.1007/s00464-025-12098-1)
- Record
  - sample width
  - stress/strain graph

Post-mortem Day 3-7
Histology Assessment

- Send slides to blinded pathologist and grade using score sheet

Visual Assessment

- Retrieve all photos and analyse in FIJI software
  - Calibrate image size
  - Measure three times and take average area for
    - Mesh size
    - Area of integration
- Examine mesh pieces
  - Remove from test tube
  - Assess signs of degradation, grade accordingly (2+ members, independently)
    - Visual
      - As it is
    - Light microscopy
      - Standard glass slide and cover slip, suitable magnification
    - SEM
      - Attached to carbon tabs, and coat with platinum as per microscopy services
      - Examine under high vacuum, 5.0kV voltage, 10mm working distance
      - Inspect entire sample at 1000x magnification
      - Record representative images

Post-mortem Day 8+

- Collate all information
- Deidentify all labels to corresponding mesh pieces
- Organise data into legible tables for each mesh
- Analyse data using predefined statistical tools and methods
