# Supplementary material for: Extended validation of the mesh integration (MINT) index: a 1-year porcine study
Source: Surg Endosc. 2026 May 4;40(7):5917–32. doi: 10.1007/s00464-026-12835-0 (PMC13369702; doi:10.1007/s00464-026-12835-0)
Supplement: Supplementary file 4 — Supplementary file4 (PDF 114 KB) [file 464_2026_12835_MOESM4_ESM.pdf]

Long-term validation of degradation sub-score of the mesh-tissue integration index, using an established porcine model

Edward Young
The University of Adelaide, North Terrace -, 5005 Adelaide, Australia
The Queen Elizabeth Hospital Basil Hetzel Institute, Woodville Road 28, 5011 Woodville South, Australia

Summary

Incisional hernias are a common post-operative complication in belly surgeries, with significant long-term disability. Achieving a satisfactory
mesh-tissue integration is key to long lasting, durable and functional hernia repairs. To provide tailored hernia care to patients, surgeons need
a tool to objectively compare the performance of mesh products in living tissue, and facilitate optimal mesh selection based on patient
comorbidities and clinical situation.

An objective numerical mesh tissue integration index was established in project SAM-23-085, which examined the mesh-tissue interface in
pigs over a 3-months period. Obtained data provided validation to the integration, fibrosis and adhesion sub-scores of the index. Data was
expectedly less conclusive for the degradation sub-score, due to the short study period.

The aim of this study is to assess mesh degradation at 1 year, using the porcine model and study protocol established in SAM-23-085.

Registration details	
Status of the study	Registered
Date of registration	2024-11-25
Date of publication	Not provided
DOI	10.17590/asr.0000371
Planned start of the study	2024-11-11
Planned end of the study	2025-12-31
License	https://creativecommons.org/licenses/by/4.0/legalcode

1. General Information

Keywords

hernia, mesh tissue integration, index, pig

Funding sources

The Queen Elizabeth Hospital Basil Hetzel Institute Research Grant The University of Adelaide / Australian Government Research and Training Program Scholarship The Hospital Research Foundation Higher Degree Research Top Up Scholarship

International code of classification

Not applicable

2. Study design

Introduction

Incisional hernia is a common complication following belly surgeries with significant long term disability and cumulative cost to the healthcare system. [1, 2] The current accepted standard of care in incisional hernia repair is to push the hernia sac back into the belly cavity with reinforcement of the belly wall via the insertion of a manufactured mesh. [3, 4] This method is superior compared to previous attempts of tension-free repair using only sutures. [5, 6] Despite advances in material sciences and surgical techniques over the past 30 years, recurrence rates of incisional hernia repairs continue to remain unacceptably high over time, with almost 50% of hernia mesh repairs failing at five years post-repair. [5]

Collective surgical experience have observed that hernia repairs fail less when the hernia opening is covered by a sufficiently large mesh, and anchored to the surrounding tissue using sufficiently strong methods. [7, 8] In recent years, these observations have been better quantified using the GRIP and CRIP concepts pioneered by Kallinowski et al. in porcine models, providing quantitative evidence that hernia repairs, at a bare minimum, need to have the ability to withstand typical forces experienced in the belly muscle wall, such as those generated during coughing in the immediate post-surgery period. [9-16] By following Kallinowski's biomechanical principles, there can be assurance that for the initial period of mesh implantation, technical failure should no longer play a major role in mesh repair failure. What is unclear at this stage is the long-term behaviour of the implanted mesh in the abdominal wall, and the changes that occur at the interface between the mesh and the local tissue.

The importance of healthy tissue infiltration into mesh, i.e. mesh tissue integration, have been recognised from past clinical experience, and microscopic examination of explanted mesh samples. Patients who experience significant complications after hernia repairs often have poorly integration hernia mesh. [17, 18] It is unknown whether these findings are due to initial variation in mesh fixation techniques, mismatch of mesh product with abdominal wall forces for a certain hernia size, or a variation in mesh tissue integration between mesh products. What is known, as demonstrated by Klinge and Klosterhalfen's work, [19] is that mesh tissue integration is closely associated with effective porosity of mesh. Too little porosity, or opening between mesh fibre strands, prevents ingrowth of normal tissue, leads to formation of dense fibrosis and results in eventual mesh shrinkage, seroma formation and chronic pain. [20-23] Based on these observations, there has been a shift in clinical practice from heavyweight microporous mesh towards lightweight macroporous, however outcomes

have not necessarily improved significantly, [24] with emerging reports of premature lightweight macroporous mesh failure, possibly due to mechanical and biological degradation. [25]

There is an urgent need to objectively quantify the biological responses and behaviours of mesh products in the belly wall. Despite over 150 hernia mesh products listed on the United States Food and Drug Administration (FDA) database and over 40 products approved for local use by the Australian Therapeutic Goods Administration (TGA), technical information on mesh products remains difficult to access on a cursory search. [26, 27] Where information is available, only compositional safety data may be freely viewed, with little information on the actual biological response of mesh products. Performance of mesh products is thus inferred from its physical properties by the clinician without clear evidence or guidance, nor is there any objective method of comparing mesh products to facilitate optimal selection and usage in the clinical setting.

Previously in project SAM-23-085, an objective numerical mesh tissue integration index was established, and validated using data obtained from a standardised porcine model over a 3-months period. A wide selection of mesh products, were inserted into clinically relevant belly muscle layers, and then explanted at pre-defined time points. Samples were analysed macroscopically, microscopically and biomechanically, using previously validated methods of assessments. [28-34]

The mesh tissue integration index is a numerical scale, which runs continuously from 0 to 5, with 0 being the worst, and 5 being the best in terms of performance. The index contains four categories, namely integration, adhesion, fibrosis and degradation. The index was designed to remain relevant to clinical practice, to remain intuitive to users, and to be structurally modular so that it may be updated when new methods or evidence arise.

The data obtained from SAM-23-085 has provided validation to the integration, fibrosis and adhesion sub-scores of index, by demonstrating variable, but steady observable changes in mesh-tissue integration over time among the investigated mesh products. Some products have a rapid onset of integration, but achieves full integration at a slower pace. Others have a relatively delayed onset, but rapidly achieves integration in a short period of time.

Data was less conclusive for degradation sub-score, though this was within design parameters. Past studies have shown degradation of polymer mesh materials require at least 12 months exposure in living tissue before changes may be observed. [28, 29] The ISO 10993-6 Biological evaluation of medical devices - Part 6: Tests for local effects after implantation guidelines recommends minimal observation time points of 3, 6, 12, 18 and 24 months in order to assess long-term stability of biomaterials implanted in living tissue. Laboratory studies often use accelerated oxidative methods, such as exposure to vapourised hydrogen peroxide (i.e. bleach), to study material degradation effects within a short time period. [35] Degradation data was obtained from SAM-23-085 to record control data, but more importantly, to reduce the overall number of experimental animals required for the mesh tissue integration index project, in line with Good Laboratory Practice and the 3R principles. [36]

The aim of this study is to assess mesh degradation at 1 year, using the porcine model and study protocol established in SAM-23-085.

Reference

1. Stabilini C, Garcia-Urena MA, Berrevoet F, Cuccurullo D, Capoccia Giovannini S, Dajko M, et al. An evidence map and synthesis review with meta-analysis on the risk of incisional hernia in colorectal surgery with standard closure. Hernia. 2022;26(2):411-36.

2. Rhemtulla IA, Hsu JY, Broach RB, Mauch JT, Serletti JM, DeMatteo RP, Fischer JP. The incisional hernia epidemic: evaluation of outcomes, recurrence, and expenses using the healthcare cost and utilization project (HCUP) datasets. Hernia. 2021;25(6):1667-75.
3. Rives J, Flament J, Delattre J, Palot J. La chirurgie moderne des hernies de l'aine. Cha Med. 1982;7:13.
4. Stoppa RE. The treatment of complicated groin and incisional hernias. World J Surg. 1989;13(5):545-54.
5. Bhardwaj P, Huayllani MT, Olson MA, Janis JE. Year-Over-Year Ventral Hernia Recurrence Rates and Risk Factors. JAMA Surg. 2024.
6. Wilson RB, Farooque Y. Risks and Prevention of Surgical Site Infection After Hernia Mesh Repair and the Predictive Utility of ACS-NSQIP. J Gastrointest Surg. 2022;26(4):950-64.
7. Kozan R, Anadol AZ, Sare M. A new criterion to predict recurrence after laparoscopic ventral hernia repair: mesh/defect area ratio. Pol Przegl Chir. 2021;93(6):40-6.
8. Tulloh B, de Beaux A. Defects and donuts: the importance of the mesh:defect area ratio. Hernia. 2016;20(6):893-5.
9. Kallinowski F, Baumann E, Harder F, Siassi M, Mahn A, Vollmer M, Morlock MM. Dynamic intermittent strain can rapidly impair ventral hernia repair. J Biomech. 2015;48(15):4026-36.
10. Kallinowski F, Fortelny RH, Köckerling F, Mayer F, Morales-Conde S, Sandblom G. Editorial: Mesh Complications in Hernia Surgery. Front Surg. 2022;9:841672.
11. Kallinowski F, Gutjahr D, Harder F, Sabagh M, Ludwig Y, Lozanovski VJ, et al. The Grip Concept of Incisional Hernia Repair-Dynamic Bench Test, CT Abdomen With Valsalva and 1-Year Clinical Results. Front Surg. 2021;8:602181.
12. Kallinowski F, Gutjahr D, Vollmer M, Harder F, Nessel R. Increasing hernia size requires higher GRIP values for a biomechanically stable ventral hernia repair. Ann Med Surg (Lond). 2019;42:1-6.
13. Kallinowski F, Harder F, Gutjahr D, Raschidi R, Silva TG, Vollmer M, Nessel R. Assessing the GRIP of Ventral Hernia Repair: How to Securely Fasten DIS Classified Meshes. Front Surg. 2017;4:78.
14. Kallinowski F, Ludwig Y, Gutjahr D, Gerhard C, Schulte-Hörmann H, Krimmel L, et al. Biomechanical Influences on Mesh-Related Complications in Incisional Hernia Repair. Front Surg. 2021;8:763957.
15. Kallinowski F, Ludwig Y, Löffler T, Vollmer M, Lösel PD, Voß S, et al. Biomechanics applied to incisional hernia repair - Considering the critical and the gained resistance towards impacts related to pressure. Clin Biomech (Bristol, Avon). 2021;82:105253.
16. Siassi M, Mahn A, Baumann E, Vollmer M, Huber G, Morlock M, Kallinowski F. Development of a dynamic model for ventral hernia mesh repair. Langenbecks Arch Surg. 2014;399(7):857-62.
17. Costello CR, Bachman SL, Grant SA, Cleveland DS, Loy TS, Ramshaw BJ. Characterization of heavyweight and lightweight polypropylene prosthetic mesh explants from a single patient. Surg Innov. 2007;14(3):168-76.
18. Morch A, Pouseele B, Doucède G, Witz JF, Lesaffre F, Lecomte-Grosbras P, et al. Experimental study of the mechanical behavior of an explanted mesh: The influence of healing. J Mech Behav Biomed Mater. 2017;65:190-9.
19. Klinge U, Klosterhalfen B. Modified classification of surgical meshes for hernia repair based on the analyses of 1,000 explanted meshes. Hernia. 2012;16(3):251-8.
20. Klinge U, Klosterhalfen B, Birkenhauer V, Junge K, Conze J, Schumpelick V. Impact of polymer pore size on the interface scar formation in a rat model. J Surg Res. 2002;103(2):208-14.
21. Klinge U, Klosterhalfen B, Conze J, Limberg W, Obolenski B, Ottinger AP, Schumpelick V. Modified mesh for hernia repair that is adapted to the physiology of the abdominal wall. Eur J Surg. 1998;164(12):951-60.
22. Klinge U, Klosterhalfen B, Müller M, Schumpelick V. Foreign body reaction to meshes used for the repair of abdominal wall hernias. Eur J Surg. 1999;165(7):665-73.

23. Karatassas A, Anthony A, Reid J, Leopardi L, Hewett P, Ibrahim N, Maddern G. Developing a mesh-tissue integration index and mesh registry database: the next step in the evolution of hernia repair. ANZ J Surg. 2018;88(6):528-9.
24. Cobb WS, Kercher KW, Heniford BT. The argument for lightweight polypropylene mesh in hernia repair. Surg Innov. 2005;12(1):63-9.
25. Petro CC, Nahabet EH, Criss CN, Orenstein SB, von Recum HA, Novitsky YW, Rosen MJ. Central failures of lightweight monofilament polyester mesh causing hernia recurrence: a cautionary note. Hernia. 2015;19(1):155-9.
26. United States Food and Drug Administration. Devices@FDA: United States Food and Drug Administration; 2024 [Available from: https://www.accessdata.fda.gov/scripts/cdrh/devicesatfda/index.cfm.
27. Australian Government Department of Health and Aged Care Therapeutic Goods Administration. Australian Register of Therapeutic Goods (ARTG) 2024 [Available from: https://www.tga.gov.au/resources/artg.
28. Lu X, Harman M, Todd Heniford B, Augenstein V, McIver B, Bridges W. Analyzing material changes consistent with degradation of explanted polymeric hernia mesh related to clinical characteristics. Surg Endosc. 2022;36(7):5121-35.
29. Wang H, Klosterhalfen B, Müllen A, Otto T, Dievernich A, Jockenhövel S. Degradation resistance of PVDF mesh in vivo in comparison to PP mesh. J Mech Behav Biomed Mater. 2021;119:104490.
30. Lacombe R. Adhesion Measurement Methods - Theory and Practice. 1st ed. United States of America: CRC Press; 2005.
31. Zhu LM, Schuster P, Klinge U. Mesh implants: An overview of crucial mesh parameters. World J Gastrointest Surg. 2015;7(10):226-36.
32. International Organization for Standardization. ISO 10993-6 Biological evaluation of medical devices - Part 6: Tests for local effects after implantation: International Organization for Standardization; 2016.
33. American Association of Tissue Banks. Standards for Tissue Banking. 14th ed. United States of America: American Association of Tissue Banks,; 2017. 189 p.
34. American Society for Testing and Materials International. ASTM F2255 Standard Test Method for Strength Properties of Tissue Adhesives in Lap-Shear by Tension Loading. United States of America: American Society for Testing and Materials International,; 2015.
35. Jain T, Tantisuwanno C, Paul A, Takmakov P, Joy A, Isayeva I, Simon DD. Accelerated in vitro oxidative degradation testing of polypropylene surgical mesh. J Biomed Mater Res B Appl Biomater. 2023;111(12):2064-76.
36. Russell W, Burch R. The Principles of Humane Experimental Technique. London: Methuen & Co. Limited; 1960.
37. Sullivan TP, Eaglstein WH, Davis SC, Mertz P. THE PIG AS A MODEL FOR HUMAN WOUND HEALING. Wound Repair Regen. 2001;9(2):66-76.
38. Gudmundsson FF, Viste A, Gislason H, Svanes K. Comparison of different methods for measuring intra-abdominal pressure. Intensive Care Med. 2002;28(4):509-14.
39. Burger JWA, Halm JA, Wijsmuller AR, Raa ST, Jeekel J. Evaluation of new prosthetic meshes for ventral hernia repair. Surgical Endoscopy and Other Interventional Techniques. 2006;20(8):1320-5.
40. Ditzel M, Deerenberg EB, Grotenhuis N, Harlaar JJ, Monkhorst K, Bastiaansen-Jenniskens YM, et al. Biologic meshes are not superior to synthetic meshes in ventral hernia repair: an experimental study with long-term follow-up evaluation. Surg Endosc. 2013;27(10):3654-62.
41. Gulmez M, Aktekin A, Aker F, Sanko V, Sezer S. Evaluation of In Vivo Adhesion Properties of New Generation Polyglactin, Oxidized Regenerated Cellulose and Chitosan-Based Meshes for Hernia Surgery. Cureus. 2021;13(10):e18755.
42. Kaufmann R, Jairam AP, Mulder IM, Wu Z, Verhelst J, Vennix S, et al. Non-Cross-Linked Collagen Mesh Performs Best in a Physiologic, Noncontaminated Rat Model. Surg Innov. 2019;26(3):302-11.

DOI 10.17590/asr.0000371

43. Schreinemacher MH, Emans PJ, Gijbels MJ, Greve JW, Beets GL, Bouvy ND. Degradation of mesh coatings and intraperitoneal adhesion formation in an experimental model. The British journal of surgery. 2009;96(3):305-13.
44. Schreinemacher MHF, Van Barneveld KWY, Dikmans REG, Gijbels MJJ, Greve JWM, Bouvy ND. Coated meshes for hernia repair provide comparable intraperitoneal adhesion prevention. Surg Endosc. 2013;27(11):4202-9.

Type of research

Exploratory

Hypothesis of your study

To assess mesh degradation at 1 year, using the porcine model and study protocol established in SAM-23-085 To assess mesh-tissue integration at 1 year, using the above samples

Study design

The study will be conducted at SAHMRI PIRL, with 3 Landrace x Large White pigs sourced by SAHMRI from a SAHMRI PIRL-approved piggery. Transportation will be arranged by SAHMRI. All animals on arrival at PIRL facility, will be given a Respisure vaccination against mycoplasma, in the form of 2mls intramuscular injection. If any pig shows signs of coughing, they will remain on antibiotics at the discretion of the duty veterinarian and local SAHMRI protocols.

Following acquisition, pigs will be acclimatised to the SAHMRI PIRL facility over a 7-day period, and longer if necessary, at the discretion of SAHMRI PIRL team. They will be given standard pig chow and water as per local SAHMRI PIRL protocols. Their environment will be enriched with stimuli, such as toys, music and human conversations with the SAHMRI PIRL animal technicians several times throughout the day. The pigs will be allowed to socialise and play with each other. Designated members of the research team will also visit the pigs prior to day of operation, to familiarise with each other.

About 1 week prior to the day of operation, the pigs will be relocated to the pre-operative area, where they will undergo a general health check by SAHMRI PIRL animal technicians to ensure they are fit for surgery. The duty veterinarian will be contacted and engaged if there are any concerns at this point. Replacement pigs may be sourced if any pig is deemed to be not suitable for the study. Pre-operative medications such as fentanyl pain relief patches, will be applied to the back of the ear at 24 hours prior to operation.

The pigs will be naturally fasted in late afternoon on the day prior to operation, as evening meal is typically given around 3pm. Thus no food restriction is required, and water is ad libitum. Pigs will undergo a final check in pre-anaesthesia area by SAHMRI PIRL animal technicians, and then be sedated, anaesthetised and intubated.

Pigs will be then be moved to the designated operating theatre, be placed supine, with belly facing towards the sky, on a pressure-relieving bedding, and secured to the operating table. A warming blanket will be provided to ward against hypothermia. Appropriate anaesthesia and ventilation settings will be applied by SAHMRI PIRL animal technicians, with constant monitoring of vital signs throughout the perioperative period. Pigs will then undergo operation to insert mesh pieces between muscle layers, as described in the Procedural section. Pigs will remain on the ventilator throughout the operation.

Operations will be performed over 1-week time. Based on the estimated time of surgery, it is likely the operations for 3 pigs will occur over 1 to 2 days. The extended window of operating is to act as

a buffer, in the event of operating theatre non-availability, equipment failure or other unforeseeable event. Operating pigs at different times will not affect overall study duration or results, due to the long study duration.

Following completion of the operations, pigs will be returned to the recovery area. Very close monitoring will occur in the immediate post-operative phase by SAHMRI PIRL animal technicians, until pigs are awake and can tolerate a light diet and water. General observations and vital signs will be recorded in the clinical record sheets at designated intervals, as illustrated and listed on the TickLab animal management system. Monitoring be downgraded in a step-wise fashion as pigs improve. Specifics of monitoring is described in the Animal Monitoring section. Antibiotics, pain relief and aperients will be given by SAHMRI PIRL staff the situation dictates. These will be weaned as pigs recover from the surgery.

Designated members of the research team will also perform ‘ward rounds’ on the pigs to ensure that their progress is in keeping with what has been observed previous in SAM-23-085 and separate clinical entries will be logged as well. If any changes in pig behaviour are observed, interventions will be taken accordingly by SAHMRI PIRL animal technicians, and if necessary, the duty veterinarian, will be consulted to make a more detailed assessment.

Once pigs have been deemed to recover from the surgery by SAHMRI PIRL veterinarian, which generally includes happy appearance, playful, tolerating a full diet and having regular large bowel motions, they will be discharged to the recovery area and moved to long-term group housing. All 3 pigs will be housed until 1 year, with standard monitoring in between, treatment of any ailments that occurs, and will be fed regular diet as per local protocol. Standard monitoring is non-invasive opportunistic general observation by SAHMRI PIRL staff of pig appearance, behaviour, food intake and bowel motions while they are inside their area of housing. Such observations made during opportunistic moments, such as at time of feeding, when their pens are cleaned, or when research team is visiting. This is aimed at maximising comfort to pigs, and minimise any stress that may be caused during the monitoring process. If any pig exhibits signs of concerns, such as sad demeaner, decreased activity or low food intake, observations will increase accordingly, and if necessary, the duty veterinarian will be alerted for review. It is expected the pigs will likely gain 2-5kg per week on a standard diet, which equals about 100-200kg weigh gain at the end of one year. It is like pig weight will plateau prior to this, and hold steady.

All 3 pigs will be humanely killed at the 1 year mark following mesh implantation. Humane killing will be performed under a terminal anaesthesia with an overdose of pentobarbitone. Death of pigs will be confirmed by SAHMRI PIRL trained staff via cessation of respiration, lack of visible or palpable heartbeat, loss of mucous membrane colour and loss of corneal reflex. Following confirmation of death, the pigs will be moved to the designated post-mortem area. Mesh tissue samples will be explanted, placed in designated specimen pots and sent for further processing and analysis.

Any premature deaths of pigs will lead to the notification of the onsite veterinarian and the animal welfare officer, along with a scheduled post-mortem with SAHMRI PIRL staff to determine cause of death. Despite the earlier time of death, where possible, mesh-tissue samples will be harvested, and analysed.

After the conclusion of post-mortems, approved research teams may scavenge tissue as needed. Pig tissue and waste will be safely sealed inside body bags and disposed of inside medical waste bins, as according to SAHMRI PIRL waste management protocols.

Method of blinding

Hernia mesh blinded to primary surgeons. Histology samples blinded to pathologist.

DOI 10.17590/asr.0000371

Method of randomization

Computer assisted randomisation, using https://www.randomizer.org/

3. Methods

3.1. Porcine Model for Hernia Mesh Insertion

Description of the method

Previously described in Patinoitt et al. 2020, and to be published data from study SAM-23-085.

The study will be conducted at SAHMRI PIRL, with 3 Landrace x Large White pigs sourced by SAHMRI from a SAHMRI PIRL-approved piggery. Transportation will be arranged by SAHMRI. All animals on arrival at PIRL facility, will be given a Respisure vaccination against mycoplasma, in the form of 2mls intramuscular injection. If any pig shows signs of coughing, they will remain on antibiotics at the discretion of the duty veterinarian and local SAHMRI protocols.

Following acquisition, pigs will be acclimatised to the SAHMRI PIRL facility over a 7-day period, and longer if necessary, at the discretion of SAHMRI PIRL team. They will be given standard pig chow and water as per local SAHMRI PIRL protocols. Their environment will be enriched with stimuli, such as toys, music and human conversations with the SAHMRI PIRL animal technicians several times throughout the day. The pigs will be allowed to socialise and play with each other. Designated members of the research team will also visit the pigs prior to day of operation, to familiarise with each other.

About 1 week prior to the day of operation, the pigs will be relocated to the pre-operative area, where they will undergo a general health check by SAHMRI PIRL animal technicians to ensure they are fit for surgery. The duty veterinarian will be contacted and engaged if there are any concerns at this point. Replacement pigs may be sourced if any pig is deemed to be not suitable for the study. Pre-operative medications such as fentanyl pain relief patches, will be applied to the back of the ear at 24 hours prior to operation.

The pigs will be naturally fasted in late afternoon on the day prior to operation, as evening meal is typically given around 3pm. Thus no food restriction is required, and water is ad libitum. Pigs will undergo a final check in pre-anaesthesia area by SAHMRI PIRL animal technicians, and then be sedated, anaesthetised and intubated.

Pigs will be then be moved to the designated operating theatre, be placed supine, with belly facing towards the sky, on a pressure-relieving bedding, and secured to the operating table. A warming blanket will be provided to ward against hypothermia. Appropriate anaesthesia and ventilation settings will be applied by SAHMRI PIRL animal technicians, with constant monitoring of vital signs throughout the perioperative period. Pigs will then undergo operation to insert mesh pieces between muscle layers, as described in the Procedural section. Pigs will remain on the ventilator throughout the operation.

Operations will be performed over 1-week time. Based on the estimated time of surgery, it is likely the operations for 3 pigs will occur over 1 to 2 days. The extended window of operating is to act as a buffer, in the event of operating theatre non-availability, equipment failure or other unforeseeable event. Operating pigs at different times will not affect overall study duration or results, due to the long study duration.

Following completion of the operations, pigs will be returned to the recovery area. Very close monitoring will occur in the immediate post-operative phase by SAHMRI PIRL animal technicians, until pigs are awake and can tolerate a light diet and water. General observations and vital signs will be recorded in the clinical record sheets at designated intervals, as illustrated and listed on the TickLab animal management system. Monitoring be downgraded in a step-wise fashion as pigs improve. Specifics of monitoring is described in the Animal Monitoring section. Antibiotics, pain relief and aperients will be given by SAHMRI PIRL staff the situation dictates. These will be weaned as pigs recover from the surgery.

Designated members of the research team will also perform ‘ward rounds’ on the pigs to ensure that their progress is in keeping with what has been observed previous in SAM-23-085 and separate clinical entries will be logged as well. If any changes in pig behaviour are observed, interventions will be taken accordingly by SAHMRI PIRL animal technicians, and if necessary, the duty veterinarian, will be consulted to make a more detailed assessment.

Once pigs have been deemed to recover from the surgery by SAHMRI PIRL veterinarian, which generally includes happy appearance, playful, tolerating a full diet and having regular large bowel motions, they will be discharged to the recovery area and moved to long-term group housing. All 3 pigs will be housed until 1 year, with standard monitoring in between, treatment of any ailments that occurs, and will be fed regular diet as per local protocol. Standard monitoring is non-invasive opportunistic general observation by SAHMRI PIRL staff of pig appearance, behaviour, food intake and bowel motions while they are inside their area of housing. Such observations made during opportunistic moments, such as at time of feeding, when their pens are cleaned, or when research team is visiting. This is aimed at maximising comfort to pigs, and minimise any stress that may be caused during the monitoring process. If any pig exhibits signs of concerns, such as sad demeaner, decreased activity or low food intake, observations will increase accordingly, and if necessary, the duty veterinarian will be alerted for review. It is expected the pigs will likely gain 2-5kg per week on a standard diet, which equals about 100-200kg weigh gain at the end of one year. It is like pig weight will plateau prior to this, and hold steady.

All 3 pigs will be humanely killed at the 1 year mark following mesh implantation. Humane killing will be performed under a terminal anaesthesia with an overdose of pentobarbitone. Death of pigs will be confirmed by SAHMRI PIRL trained staff via cessation of respiration, lack of visible or palpable heartbeat, loss of mucous membrane colour and loss of corneal reflex. Following confirmation of death, the pigs will be moved to the designated post-mortem area. Mesh tissue samples will be explanted, placed in designated specimen pots and sent for further processing and analysis.

Any premature deaths of pigs will lead to the notification of the onsite veterinarian and the animal welfare officer, along with a scheduled post-mortem with SAHMRI PIRL staff to determine cause of death. Despite the earlier time of death, where possible, mesh-tissue samples will be harvested, and analysed.

After the conclusion of post-mortems, approved research teams may scavenge tissue as needed. Pig tissue and waste will be safely sealed inside body bags and disposed of inside medical waste bins, as according to SAHMRI PIRL waste management protocols.

Narcotic/analgesic treatment

Not applicable

Drugs/substances

Not applicable

Antibodies

Not applicable

Cell lines, viruses, DNA or RNA constructs and bacteria

Not applicable

4. Statistics

4.1. Mean, median, standard deviation

Assigned method(s)

Porcine Model for Hernia Mesh Insertion

Main endpoints

Mean and 95% confidence interval for results of each component in each item of testing, with particular focus on mesh degradation and integration scores.

Secondary endpoints

Not applicable

Sample size calculation

Not applicable

Primary statistical analysis

Mean, median and standard deviation of results for each time point, as multiple samples are taken.

Exclusion criteria

Not applicable.

4.2. Regression analysis, line of best fit

Assigned method(s)

Porcine Model for Hernia Mesh Insertion

Main endpoints

Test results, combined into total composite score over time for each type of mesh.

Secondary endpoints

Not applicable

Sample size calculation

Not applicable

DOI 10.17590/asr.0000371

Primary statistical analysis

line of best fit, linear/logistic regression analysis

Exclusion criteria

Not applicable

5. Animals

5.1. Pigs (Sus scrofa domesticus)

Animal strain/breed

Landrace/Large White pigs

Genetically modified

No

Sex

Female
Male

Further characteristics of the animals (e.g. age, body weight, size)

30-35kg, about 11-12 weeks old

Housing conditions

Study was conducted at the South Australian Health and Medical Research Institute (SAHMRI) Preclinical Imaging and Research Laboratories (PIRL), located at Gilles Plains, South Australia, Australia. This facility covers a 35-acre grasslands and is the only purpose-built large and small animal National Association of Testing Authorities (NATA)-accredited research facility (accreditation no. 19120) in Australia where surgical theatres and imaging facility are adjacent to quality indoor and outdoor animal holding facilities. 43 This facility is notable for five large surgical theatres; dedicated pre-operative and post-operative recovery areas; logical room progression from indoor pen holding area, to undercover pens, to paddocks; designated post-mortem room and sterilisation amenities to support material preparations. 43

Refinement

Following purchase of pigs from the designated piggery, the pigs were transported to SAHMRI PIRL facility in accordance to state and national guidelines. Upon arrival, pigs were given a 2ml intramuscular inactivated Glaesserella parasuis vaccination injection as a precaution against mycoplasma. If any pigs showed signs of coughing, antibiotics were given for the duration of the study as per local SAHMRI protocol.

For the initial 10 days, pigs were given protected time, and was allowed to socialise and acclimatise with each other. No study procedures or interventions were carried out during this time. SAHMRI animal technicians were in the holding area multiple times throughout the day and talked to the pigs to familiarise the animals to humans. The living area were made comfortable with rubber mats. The pigs were given access to ad libitum water, nutrition and appropriate stimuli for enrichment, such as balls and toys.

DOI 10.17590/asr.0000371
