# Supplementary material for: Extended validation of the mesh integration (MINT) index: a 1-year porcine study
Source: Surg Endosc. 2026 May 4;40(7):5917–32. doi: 10.1007/s00464-026-12835-0 (PMC13369702; doi:10.1007/s00464-026-12835-0)
Supplement: Supplementary file 5 — Supplementary file5 (DOCX 52 KB) [file 464_2026_12835_MOESM5_ESM.docx]

# Supplementary Table Legend

**Supplementary Table 1** – Summary of speed of pig recovery and weight change during study

**Supplementary Table 2** – Assessment scores and overall score for Integration domain

**Supplementary Table 3** – Assessment scores and overall score for Fibrosis domain

**Supplementary Table 4** – Assessment scores and overall score for Degradation domain

**Supplementary Table 5** – Correlation assessment between factors and Index scores, using Kendall’s tau, assuming non-parametric small data set with independence between factors.

**Supplementary Table 6** – Multilevel linear and nonlinear modelling of Index scores, controlling for variations between pigs.

# Supplementary Table 1

Summary of speed of pig recovery and weight change during study

| Pig | CRS return to baseline post-operative (days) | Weight, pre-operation (kg) | Weight, post-mortem (kg) | Average growth per week (kg/week) |
| --- | --- | --- | --- | --- |
| 1 | 4 | 33 | 184 | 2.9 |
| 2 | 2 | 32.5 | 168 | 2.6 |
| 3 | 1 | 34 | 200 | 3.2 |

CRS: Clinical Record Sheet

# Supplementary Table 2

Assessment scores and overall score for Integration domain

| Integration |  | V. | Histological | | | | | | | | | | Biomechanical | | | |  |
| --- | --- | --- | --- | --- | --- | --- | --- | --- | --- | --- | --- | --- | --- | --- | --- | --- | --- |
| Mesh Name | Time (Weeks) | % visual integration | Polymorphonuclear leukocytes | Lymphocytes | Plasma cells | Macrophages | Giant cells | Necrosis | Cellular infiltration | Neovascularisation | Connective tissue deposition | % integration histology | $\tau_{elastic limit}$ (N/cm^2^) | % shear ratio, elastic | $\tau_{ultimate limit}$ (N/cm^2^) | % shear ratio, ultimate | **TOTAL SCORE** |
| Parietene Macroporous*  Medtronic | 52 | 1 | 0 | 0 | 0 | 1 | 1 | 0 | 3 | 3 | 4 | 0.75 | 12.50 | 0.26 | 17.39 | 0.36 | **2.97** |
|  | 52 | 1 | 0 | 1 | 0 | 2 | 2 | 0 | 3 | 2 | 3 | 0.46 | 33.18 | 0.69 | 42.56 | 0.89 | **3.80** |
|  | 52 | 1 | 0 | 0 | 0 | 0 | 0 | 0 | 3 | 2 | 0 | 0.42 | 9.85 | 0.21 | 16.44 | 0.34 | **2.46** |
| Phasix*  Bard | 52 | 1 | 0 | 1 | 0 | 2 | 2 | 0 | 3 | 4 | 4 | 0.71 | 17.10 | 0.36 | 17.25 | 0.36 | **3.03** |
|  | 52 | 1 | 0 | 0 | 0 | 0 | 0 | 0 | 3 | 2 | 0 | 0.42 | 21.59 | 0.45 | 21.97 | 0.46 | **2.91** |
|  | 52 | 1 | 0 | 0 | 0 | 1 | 1 | 0 | 3 | 4 | 4 | 0.83 | 19.16 | 0.40 | 20.68 | 0.43 | **3.33** |
| Polypropylene*  Bard | 52 | 1 | 0 | 0 | 0 | 1 | 1 | 0 | 3 | 4 | 4 | 0.83 | 10.95 | 0.23 | 16.98 | 0.35 | **3.02** |
|  | 52 | 1 | 0 | 1 | 0 | 2 | 2 | 0 | 3 | 4 | 3 | 0.63 | 22.75 | 0.47 | 24.29 | 0.51 | **3.26** |
|  | 52 | 1 | 0 | 1 | 0 | 2 | 2 | 0 | 3 | 2 | 3 | 0.46 | 11.80 | 0.25 | 16.18 | 0.34 | **2.55** |
| Progrip*  Medtronic | 52 | 1 | 0 | 0 | 0 | 1 | 1 | 0 | 3 | 4 | 2 | 0.67 | 14.44 | 0.30 | 16.46 | 0.34 | **2.89** |
|  | 52 | 1 | 0 | 1 | 0 | 2 | 2 | 0 | 2 | 2 | 3 | 0.38 | 17.38 | 0.36 | 29.40 | 0.61 | **2.94** |
|  | 52 | 1 | 0 | 1 | 0 | 2 | 2 | 0 | 3 | 4 | 4 | 0.71 | 15.36 | 0.32 | 27.36 | 0.57 | **3.25** |
| Soft* Bard | 52 | 1 | 0 | 0 | 0 | 1 | 1 | 0 | 3 | 3 | 4 | 0.75 | 13.38 | 0.28 | 17.98 | 0.37 | **3.00** |
|  | 52 | 1 | 0 | 0 | 0 | 0 | 0 | 0 | 3 | 1 | 0 | 0.33 | 33.00 | 0.69 | 47.88 | 1.00 | **3.77** |
|  | 52 | 1 | 0 | 1 | 0 | 2 | 2 | 0 | 3 | 4 | 3 | 0.63 | 18.90 | 0.39 | 18.90 | 0.39 | **3.02** |
| TIGR  Novus Scientific | 52 | 1 | 0 | 0 | 0 | 0 | 0 | 0 | 3 | 2 | 0 | 0.42 | 38.40 | 0.80 | 39.74 | 0.83 | **3.81** |
|  | 52 | 1 | 0 | 2 | 0 | 3 | 2 | 0 | 3 | 4 | 3 | 0.54 | 19.09 | 0.40 | 20.28 | 0.42 | **2.95** |
|  | 52 | 1 | 0 | 1 | 0 | 2 | 3 | 0 | 3 | 3 | 2 | 0.42 | 10.82 | 0.23 | 10.99 | 0.23 | **2.34** |

V.: visual

*: denotes expired mesh

# Supplementary Table 3

Assessment scores and overall score for Fibrosis domain

| Fibrosis |  | V. | Histology | | | | |  |
| --- | --- | --- | --- | --- | --- | --- | --- | --- |
| Mesh Name | Time (Weeks) | % visual shrinkage | Fibrosis | Fatty infiltration | Fibrous encapsulation | Mineralisation | % fibrosis histology | **TOTAL SCORE** |
| Parietene Macroporous*  Medtronic | 52 | -0.24 | 3 | 0 | 4 | 3 | 0.63 | **0.97** |
|  | 52 | -0.10 | 2 | 0 | 3 | 3 | 0.50 | **0.99** |
|  | 52 | -0.19 | 0 | 0 | 0 | 0 | 0 | **-0.47** |
| Phasix*  Bard | 52 | -0.33 | 3 | 0 | 4 | 0 | 0.44 | **0.28** |
|  | 52 | -0.17 | 0 | 0 | 0 | 0 | 0 | **-0.43** |
|  | 52 | -0.10 | 4 | 0 | 4 | 0 | 0.50 | **1.00** |
| Polypropylene*  Bard | 52 | -0.28 | 3 | 0 | 4 | 2 | 0.56 | **0.72** |
|  | 52 | -0.23 | 3 | 0 | 3 | 2 | 0.50 | **0.66** |
|  | 52 | -0.03 | 3 | 0 | 3 | 2 | 0.50 | **1.19** |
| Progrip*  Medtronic | 52 | 0.30 | 2 | 0 | 3 | 0 | 0.31 | **1.52** |
|  | 52 | 0.04 | 3 | 0 | 4 | 1 | 0.50 | **1.36** |
|  | 52 | 0.07 | 3 | 0 | 4 | 0 | 0.44 | **1.26** |
| Soft* Bard | 52 | -0.12 | 3 | 0 | 4 | 2 | 0.56 | **1.11** |
|  | 52 | 0.08 | 0 | 0 | 0 | 0 | 0 | **0.19** |
|  | 52 | -0.05 | 3 | 0 | 4 | 2 | 0.56 | **1.27** |
| TIGR  Novus Scientific | 52 | -0.24 | 0 | 0 | 0 | 0 | 0 | **-0.60** |
|  | 52 | -0.05 | 2 | 0 | 3 | 0 | 0.31 | **0.66** |
|  | 52 | -0.05 | 2 | 0 | 3 | 0 | 0.31 | **0.65** |

V.: visual

*: denotes expired mesh

-: data not available

# Supplementary Table 4

Assessment scores and overall score for Degradation domain

| Degradation |  | V. | Histo. | | Biomech. | | Molecular | |  |
| --- | --- | --- | --- | --- | --- | --- | --- | --- | --- |
| Mesh Name | Time (Weeks) | % visual degradation | Implant degradation | % degradation histology | Mesh tensile strength, ultimate (N/cm) | %$\Delta$mesh tensile strength | %$\Delta$Carboxyl Index | % MAD∆absorption | **TOTAL SCORE** |
| Parietene Macroporous*  Medtronic | 52 | 0.25 | 3 | 0.75 | 28.23 | -0.11 | 0.78 | 0.51 | **2.17** |
|  | 52 | 0.63 | 2 | 0.5 | 35.07 | -0.38 | 0.63 | 0.35 | **1.73** |
|  | 52 | 0.50 | 3 | 0.75 | 29.44 | -0.16 | 0.70 | 0.43 | **2.22** |
| Phasix*  Bard | 52 | 1 | 2 | 0.5 | 0 | 1 | - | 0.16 | **3.32** |
|  | 52 | 1 | 4 | 1 | 0 | 1 | - | 0.27 | **4.08** |
|  | 52 | 1 | 3 | 0.75 | 0 | 1 | - | 0.27 | **3.77** |
| Polypropylene*  Bard | 52 | 0.25 | 3 | 0.75 | 37.94 | 0.17 | 0.59 | 1 | **2.77** |
|  | 52 | 0.50 | 2 | 0.5 | 42.04 | 0.09 | 0.48 | 1 | **2.56** |
|  | 52 | 0.50 | 1 | 0.25 | 54.57 | -0.19 | 0.43 | 1 | **2.00** |
| Progrip*  Medtronic | 52 | 0.13 | 1 | 0.25 | 6.12 | 0.74 | -0.04 | 1 | **2.08** |
|  | 52 | 0.38 | 1 | 0.25 | 13.31 | 0.43 | 1 | 0.86 | **2.92** |
|  | 52 | 0.13 | 1 | 0.25 | 9.38 | 0.60 | 1 | 0.41 | **2.38** |
| Soft* Bard | 52 | 0 | 3 | 0.75 | 23.38 | 0.56 | 0.75 | 1 | **3.06** |
|  | 52 | 0.25 | 3 | 0.75 | 41.98 | 0.21 | 0.82 | 1 | **3.03** |
|  | 52 | 0.50 | 1 | 0.25 | 9.44 | 0.82 | 0.65 | 1 | **3.22** |
| TIGR  Novus Scientific | 52 | 1 | 4 | 1 | 0 | 1 | - | 0.75 | **4.69** |
|  | 52 | 1 | 2 | 0.5 | 0 | 1 | - | 0.19 | **3.37** |
|  | 52 | 1 | 4 | 1 | 0 | 1 | - | 0.55 | **4.43** |

V.: visual

Histo.: histological

Biomech.: biomechanical
% MAD∆absorption: mean absolute deviation of percentage change in spectra peaks’ absorption values

*: denotes expired mesh

-: data not available

# Supplementary Table 5

Correlation assessment between factors and Index scores, using Kendall’s tau, assuming non-parametric small data set with independence between factors.

| Correlation | Score | Kendall’s tau | p-value |
| --- | --- | --- | --- |
| Time vs. Score | Integration | 0.771 | <0.001 |
|  | Fibrosis | 0.137 | 0.086 |
|  | Degradation | 0.424 | <0.001 |
| Mesh vs. Score | Integration | -0.071 | 0.377 |
|  | Fibrosis | -0.073 | 0.359 |
|  | Degradation | 0.087 | 0.275 |

# Supplementary Table 6

Multilevel linear and nonlinear modelling of Index scores, controlling for variations between pigs. P-value signifies whether there is any statistical significant difference between a model and the previous model in the line above.

| Multilevel Modelling | AIC | BIC | -2LL | R squared | RMSE | ICC | Chi Sq. | df. | p-value, for Chi Sq. |
| --- | --- | --- | --- | --- | --- | --- | --- | --- | --- |
| Integration Score |  |  |  |  |  |  |  |  |  |
| Score ~ 1 + (1\|Pig) | 129.30 | 136.60 | 123.30 | 0 | 0.340 | 0.863 | - | - | - |
| Score ~ 1 + (1\|Mesh) + (1\|Pig) | 130.68 | 140.41 | 122.68 | 0 | 0.328 | 0.869 | 0.62 | 1 | 0.431 |
| Score ~ Time + (1\|Mesh) + (1\|Pig) | 113.56 | 125.71 | 103.56 | 0.662 | 0.331 | 0.614 | 19.13 | 1 | <0.001 |
| Score ~ poly(Time, 2) + (1\|Mesh) + (1\|Pig) | 91.76 | 106.35 | 79.76 | 0.846 | 0.347 | 0.149 | 23.79 | 1 | <0.001 |
| Score ~ poly(Time, 3) + (1\|Mesh) + (1\|Pig) | 89.85 | 106.87 | 75.85 | 0.856 | 0.353 | 0.087 | 3.91 | 1 | 0.048 |
| Score ~ poly(Time, 4) + (1\|Mesh) + (1\|Pig) | 91.22 | 110.67 | 75.22 | 0.858 | 0.354 | 0.078 | 0.63 | 1 | 0.428 |
| Score ~ SSlogis(Weeks, Asym, xmid, scal) ~ (Asym\|Mesh) + (xmid\|Pig) | 98.30 | 112.89 | 86.30 | 0.617 | 0.370 | NA | 11.08 | 0 | NA |
| Score ~ SSasymp(Weeks, Asym, R0, lrc) ~ (Asym\|Mesh) + (R0\|Pig) | 88.46 | 103.05 | 76.462 | 0.971 | 0.370 | NA | 9.84 | 0 | NA |
| Score ~ SSbiexp(Time, A1, lrc1, A2, lrc2) ~ (A1\|Mesh) + (A2\|Pig) | - | - | - | - | - | - | - | - | - |
| Fibrosis Score |  |  |  |  |  |  |  |  |  |
| Score ~ 1 + (1\|Pig) | 152.85 | 160.15 | 146.85 | 0 | 0.521 | 0.156 | - | - | - |
| Score ~ 1 + (1\|Mesh) + (1\|Pig) | 154.85 | 164.58 | 146.85 | 0 | 0.521 | NA | 0 | 1 | 1 |
| Score ~ Time + (1\|Mesh) + (1\|Pig) | 156.34 | 168.49 | 146.34 | 0.013 | 0.521 | NA | 0.51 | 1 | 0.473 |
| Score ~ poly(Time, 2) + (1\|Mesh) + (1\|Pig) | 149.87 | 164.46 | 137.87 | 0.144 | 0.541 | NA | 8.46 | 1 | 0.004 |
| Score ~ poly(Time, 3) + (1\|Mesh) + (1\|Pig) | 145.47 | 162.48 | 131.47 | 0.206 | 0.529 | NA | 3.45 | 1 | 0.063 |
| Score ~ poly(Time, 4) + (1\|Mesh) + (1\|Pig) | 143.07 | 162.52 | 127.07 | 0.247 | 0.516 | NA | 0.50 | 1 | 0.481 |
| Score ~ SSlogis(Weeks, Asym, xmid, scal) ~ (Asym\|Mesh) + (xmid\|Pig) | - | - | - | - | - | - | - | - | - |
| Score ~ SSasymp(Weeks, Asym, R0, lrc) ~ (Asym\|Mesh) + (R0\|Pig) | 146.92 | 161.51 | 134.92 | 0.392 | 0.540 | NA | 2.95 | 0 | NA |
| Score ~ SSbiexp(Time, A1, lrc1, A2, lrc2) ~ (A1\|Mesh) + (A2\|Pig) | 141.57 | 158.59 | 127.57 | 0.941 | 0.517 | NA | 3.90 | 0 | NA |
| Degradation Score |  |  |  |  |  |  |  |  |  |
| Score ~ 1 + (1\|Pig) | 260.50 | 267.80 | 254.5 | 0 | 0.870 | 0.487 | - | - | - |
| Score ~ 1 + (1\|Mesh) + (1\|Pig) | 243.87 | 253.59 | 235.87 | 0 | 0.678 | 0.674 | 18.63 | 1 | <0.001 |
| Score ~ Time + (1\|Mesh) + (1\|Pig) | 218.60 | 230.76 | 254.50 | 0.499 | 0.718 | 0.335 | 27.26 | 1 | <0.001 |
| Score ~ poly(Time, 2) + (1\|Mesh) + (1\|Pig) | 220.57 | 235.15 | 235.87 | 0.499 | 0.718 | 0.335 | 0.04 | 1 | 0.850 |
| Score ~ poly(Time, 3) + (1\|Mesh) + (1\|Pig) | 213.83 | 230.85 | 208.60 | 0.630 | 0.717 | NA | 8.73 | 1 | 0.003 |
| Score ~ poly(Time, 4) + (1\|Mesh) + (1\|Pig) | 215.28 | 234.72 | 208.57 | 0.632 | 0.714 | NA | 0.55 | 1 | 0.456 |
| Score ~ SSlogis(Weeks, Asym, xmid, scal) ~ (Asym\|Mesh) + (xmid\|Pig) | 229.91 | 244.50 | 199.83 | 0.984 | 0.817 | NA | 0 | 0 | NA |
| Score ~ SSasymp(Weeks, Asym, R0, lrc) ~ (Asym\|Mesh) + (R0\|Pig) | 233.31 | 247.89 | 199.28 | 0.855 | 0.815 | NA | 0 | 0 | NA |
| Score ~ SSbiexp(Time, A1, lrc1, A2, lrc2) ~ (A1\|Mesh) + (A2\|Pig) | - | - | - | - | - | - | - | - | - |

AIC: Akaike information criterion

BIC: Bayesian information criterion

-2LL: -2 log-likelihoods, also known as deviance

RMSE: root mean square error

Chi Sq.: Chi Square

df.: degrees of freedom

SSasymp: self start asymptotic regression model in R

Asym: asymptote

A1: asymptote

A2: asymptote

Xmid: inflection point at x-distance from origin

Scal: angular coefficient of the slope at point of inflection

R0: starting point

Lrc: natural logarithm of rate of constant

Poly: polynomial regression model in R

NA: not available
